# Balancing Reactivity, Regioselectivity, and Product Stability in Ir-Catalyzed Ortho-C–H Borylations of Anilines by Modulating the Diboron Partner

**DOI:** 10.1021/acs.orglett.4c01495

**Published:** 2024-06-26

**Authors:** Jose R. Montero Bastidas, Anshu Yadav, Seokjoo Lee, Behnaz Ghaffari, Milton R. Smith, Robert E. Maleczka

**Affiliations:** Department of Chemistry, Michigan State University, 578 South Shaw Lane, East Lansing, Michigan 48824, United States

## Abstract

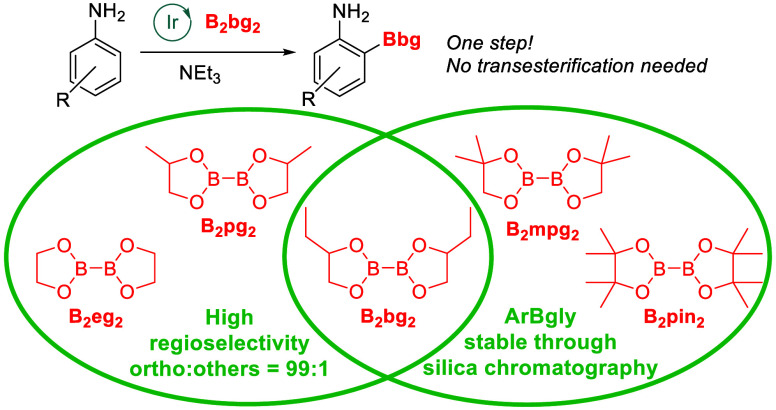

Ir-catalyzed arene C–H borylations (CHB) of anilines can be highly ortho selective by using a small B_2_eg_2_ (eg = ethane-1,2-diol) as the borylating reagent. Unfortunately, the products are prone to decomposition, and transesterification with pinacol is required prior to isolation. This work offers a solution by adjusting the size of the diboron reagent. Based on our evaluation, we conclude that B_2_bg_2_ (bg = butane-1,2-diol) achieves an optimal balance between CHB regioselectivity and stability for the borylated products.

Selective replacement of ubiquitous C–H bonds with Bpin opens the door to diverse compound modifications. For example, Suzuki–Miyaura cross-couplings enable the substitution of Bpin with alkyl and aryl groups, while Chan–Lam couplings facilitate the exchange of C–Bpin groups with C–O or C–N functionalities.^1^ Iridium-catalyzed C–H borylation (CHB) stands as a reliable and established method for introducing Bpin groups, with regioselectivity highly governed by steric factors.^2−5^ CHB of 1,3-disubstituted arenes exhibits remarkable selectivity for the C5-borylated products, regardless of the electronic nature of the substituents present. However, it is possible to switch the CHB regioselectivity to the less sterically accessible ortho position by modifying the ligand or employing different directing groups.^6^ Accordingly, the employment of strategically designed ligands enable ortho CHB of anilines bearing acyl, silyl or methylthiomethyl directing groups.^7−9^ Despite these successes, methods that bypass the need for preinstalled directing groups would be highly advantageous.

Previously, in collaboration with the Singleton group, we discovered the preference of *N*-(Boc)-anilines to yield the ortho-borylated product under standard CHB conditions.^10^ The unexpected selectivity was attributed to an N–H···O hydrogen bonding interaction between the hydrogen of the aniline and one of the Bpin ligands on the iridium catalyst. One year later, we reported a method for the ortho-borylation of anilines without a preinstallation of a directing group by using HBpin as the boron partner (Figure 1, Method A).^11^ The proposed mechanism suggests the initial formation of ArNH–Bpin, succeeded by ortho-C–H borylation controlled by a hydrogen bond interaction (N–H···O) akin to that suggested for N-(Boc)-anilines. Unfortunately, this method worked well exclusively on substrates with substituents para to the NH_2_ group, and selectivities were considerably diminished without that substitution.

**Figure 1:**
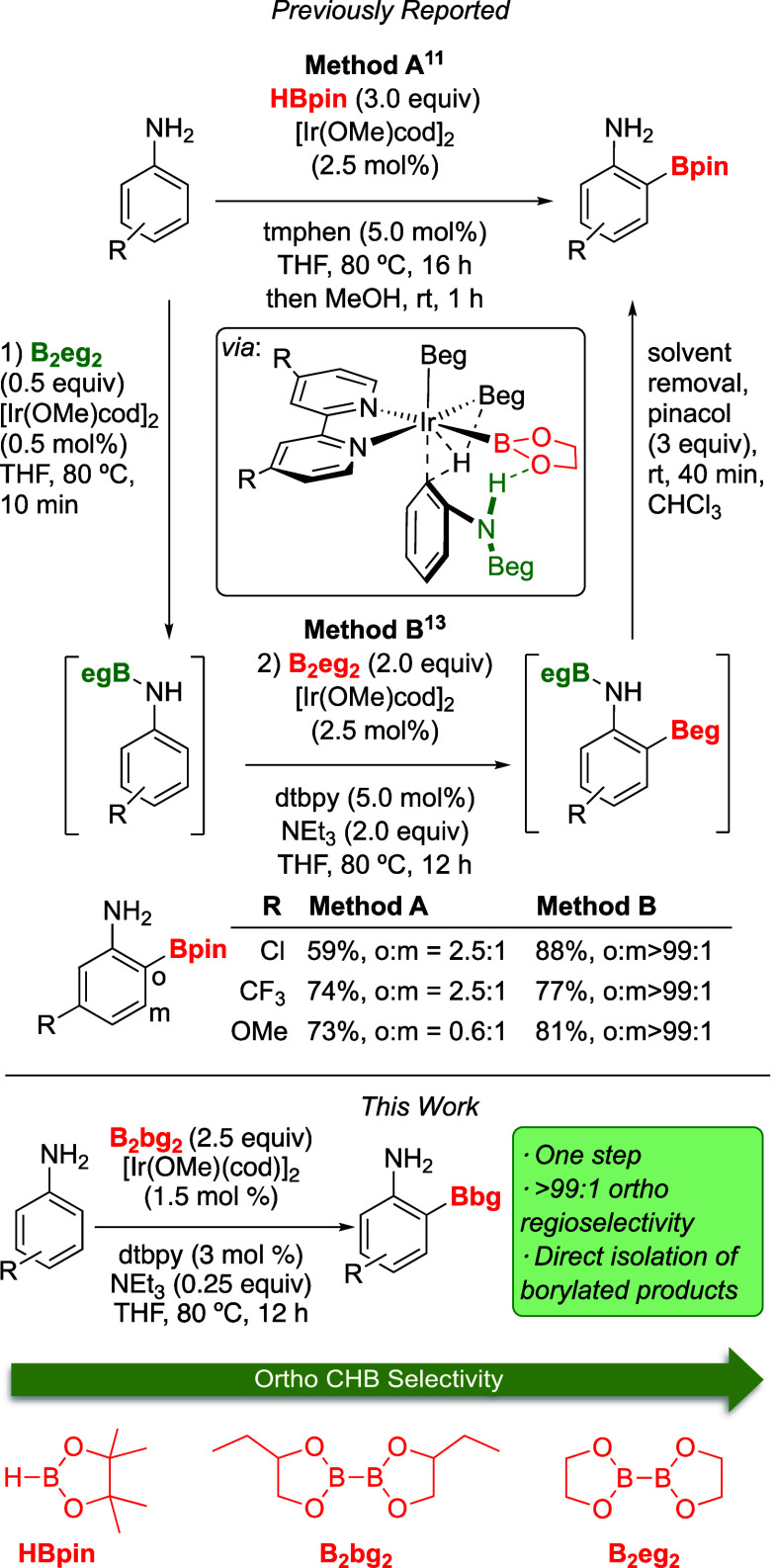
Ortho-CHB of anilines.

In 2017, our research revealed that phenols have a propensity to form ortho-borylated products, albeit with low selectivity in the presence of other sterically available C–H bonds. The formation of the ortho-borylated product was attributed to an electrostatic interaction between the partial negatively charged OBpin group formed in situ and the partial positively charged bipyridine ligand. Remarkably, the utilization of a small diboron partner B_2_eg_2_ led to high regioselectivity for the ortho-borylation without detectable formation of the meta and para borylated products.^12^ Inspired by this work, we and the Chattopadhyay group showed that using B_2_eg_2_ as the boron reagent during the borylation of anilines also gives high ortho regioselectivity without the necessity of a para substituent (Figure 1, Method B).^13^ This enhanced selectivity is a result of the reduced steric hindrance of the Beg group, which provides stability to the transition state. However, the (2-Beg)ArNH_2_ product must be converted to the more stable (2-Bpin)ArNH_2_, by treatment with pinacol prior to purification. Eliminating the need for the final transesterification step while retaining the regioselectivity of the reaction mixture would be highly desirable and advantageous. Recently, the Yan group discovered that employing mesoionic carbene-Ir catalysts enables high regioselectivities in the ortho CHB of anilines with B_2_pin_2_.^14^ Traditionally, the focus in achieving high selectivities in CHB reactions has centered on extensive ligand screening, while the influence of the diboron reagent employed has been a less-explored facet.^12,13,15,16^

We proposed that using boronic partners with larger substituents than B_2_eg_2_ could lead to more stable ortho-borylated anilines, possibly enabling direct isolation of the product.^17^ However, there is a risk of reducing the regioselectivity of ortho CHB in the process. Thus, our objective was to strike a balance between maintaining high regioselectivity and achieving increased stability of the borylated product by careful optimization of the diboron partner.

A diversity of diboron partners can be imagined with a range of sizes between those of B_2_pin_2_ and B_2_eg_2_. We began this investigation with the most straightforward choice, B_2_pg_2_ (pg = propane-1,2-diol), which presents a single methyl group in each glycolate group of B_2_eg_2_ and for which there are examples where arenes bearing a Bpg group afford higher yields than the corresponding Bpin bearing substrates in Suzuki–Miyaura cross-couplings.^18^ We were pleased when the initial CHBs (Scheme 1) on aniline with B_2_pg_2_ afforded the ortho product, albeit with slightly lower conversions when compared to reactions with B_2_eg_2_.^13^

**Scheme 1:**
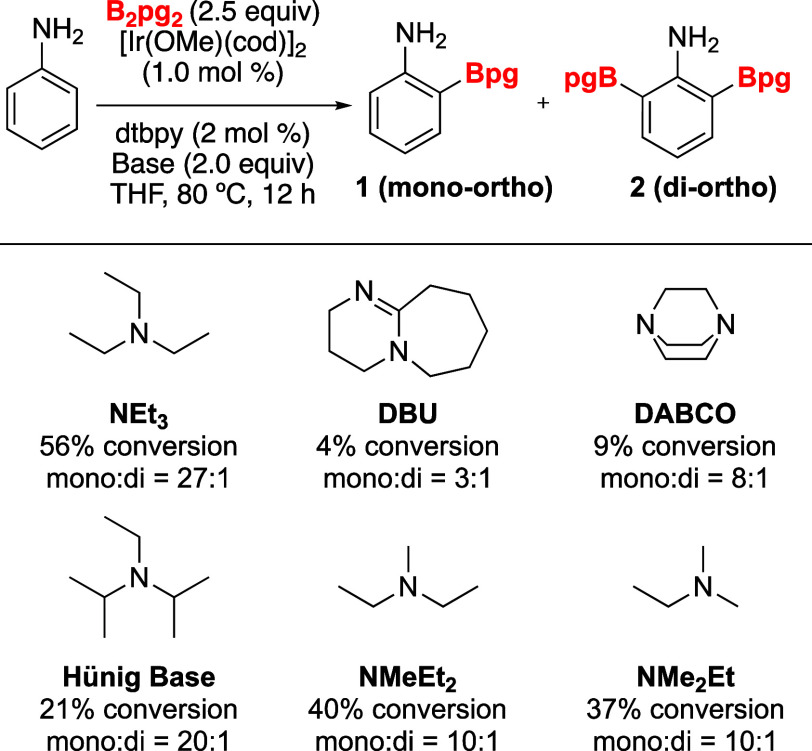
Base Effect on the Ortho-CHB of Aniline Using B_2_pg_2_ and the Boron Source

To improve this result, we first decided to explore different amine additives. In earlier studies,^12,13^ the presence of triethyl amine improved the ortho selectivity in the CHB of phenols and anilines with B_2_eg_2_. It was proposed that the H–Beg side product formed a stabilizing complex with the amine,^19,20^ which in turn retarded undesirable reactions. Thus, to assess the effect of the amine, diisopropylethylamine (Hünig base), DBU, and DABCO were tested as potential alternatives to triethylamine using B_2_pg_2_ as the boron source on the ortho-CHB of aniline. As seen in Scheme 1, a lower reactivity was observed with these amines. Thinking that counter to our original idea the poor results might be due to the HBpg·amine complex being destabilized by sterics, we proceeded to test diethylmethyl amine and ethyldimethyl amine. Despite the improved conversions, triethylamine remained to be the most effective, yielding the highest conversion and selectively at producing the ortho-monoborylated product.

The impact of triethylamine stoichiometry on the ortho-borylation of anilines was evaluated next, and the results are depicted in Figure 2. Conversions leading to the ortho monoborylated aniline are represented by the blue bars, while the orange bars illustrate the diborylated product. Unexpectedly, lowering the amount of Et_3_N improved conversions with 0.25 equiv being optimal. Though the reasons for improved conversions are unclear, perhaps it is the excess equivalents of base break the N–B bond of the intermediate PhN(H)Bpg causing loss of reactivity. Using less than 0.25 equiv of Et_3_N was met with negative results, but we note that the CHB proceeded when no amine was present.

**Figure 2:**
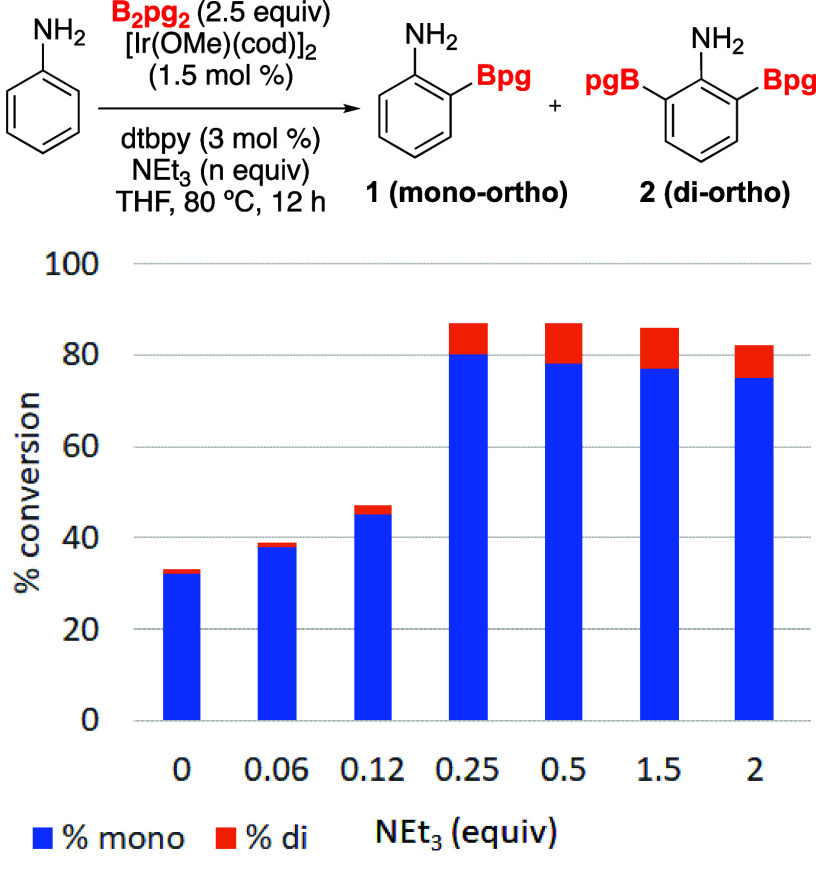
Screening of triethylamine equivalents on ortho-CHB of aniline.

After the optimized equivalents of the amine were determined, our focus shifted to evaluating various diboron partners to identify the optimal balance between ortho regioselectivity and stability of the borylated product for the CHB of anilines.

Before moving beyond B_2_pg_2_, we conducted experiments to compare the selectivity induced by each stereoisomer of B_2_pg_2_ with that of the mixture of stereoisomers (Scheme 2). Pure (*S,S*)-B_2_pg_2_ was synthesized from (*S*)-propylene glycol and subjected to CHB conditions. Interestingly, the reactivity and regioselectivity with (*S*,*S*)-B_2_pg_2_ were comparable to those seen with the stereoisomer mixture. This suggests that all diastereomers present in the B_2_pg_2_ mixture react similarly during ortho-CHB of aniline. Unfortunately, the use of B_2_pg_2_ did not solve the stability issues seen with B_2_eg_2_ as deborylation occurred during purification of the crude reaction products.

**Scheme 2:**
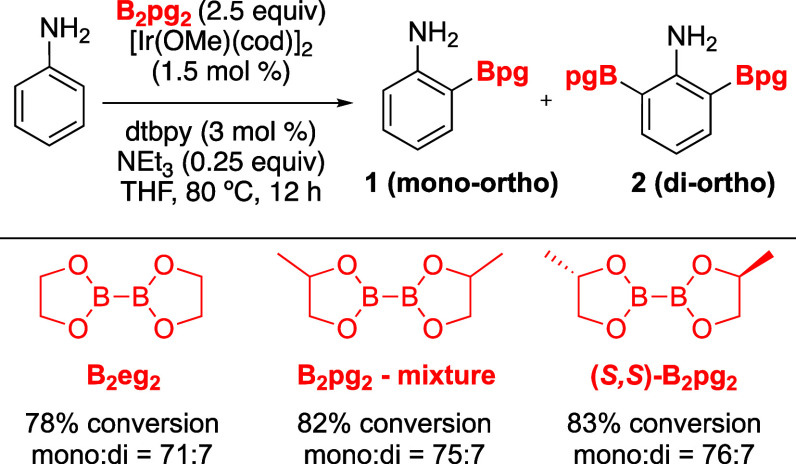
CHB Regioselectivity of Aniline with B_2_eg_2_, Racemic B_2_pg_2_, and (*S*)-B_2_pg_2_

The trouble encountered during the isolation of ArBpg products heightened the desire to test additional boron partners. Therefore, three larger diboron partners, B_2_bg_2_ with its ethyl pendant group, the gem-dimethyl bearing B_2_mpg_2_, and B_2_((2*R*,3*R*)bg)_2_, were synthesized from the corresponding glycol and B_2_(OH)_4_. These diboron reagents were used in the CHB of unsubstituted aniline with 0.0, 0.25, and 2.0 equiv of triethylamine (Figure 3). As observed with B_2_pg_2_, the highest reactivity in each case was achieved with 0.25 equiv of the base. The high regioselectivity for ortho-CHB of aniline remained with the diboron partners having only one pendant alkyl group (B_2_pg_2_ and B_2_bg_2_), but it was reduced when additional methyls were introduced (B_2_mpg_2_ and B_2_((2*R*,3*R*)bg)_2_).

**Figure 3:**
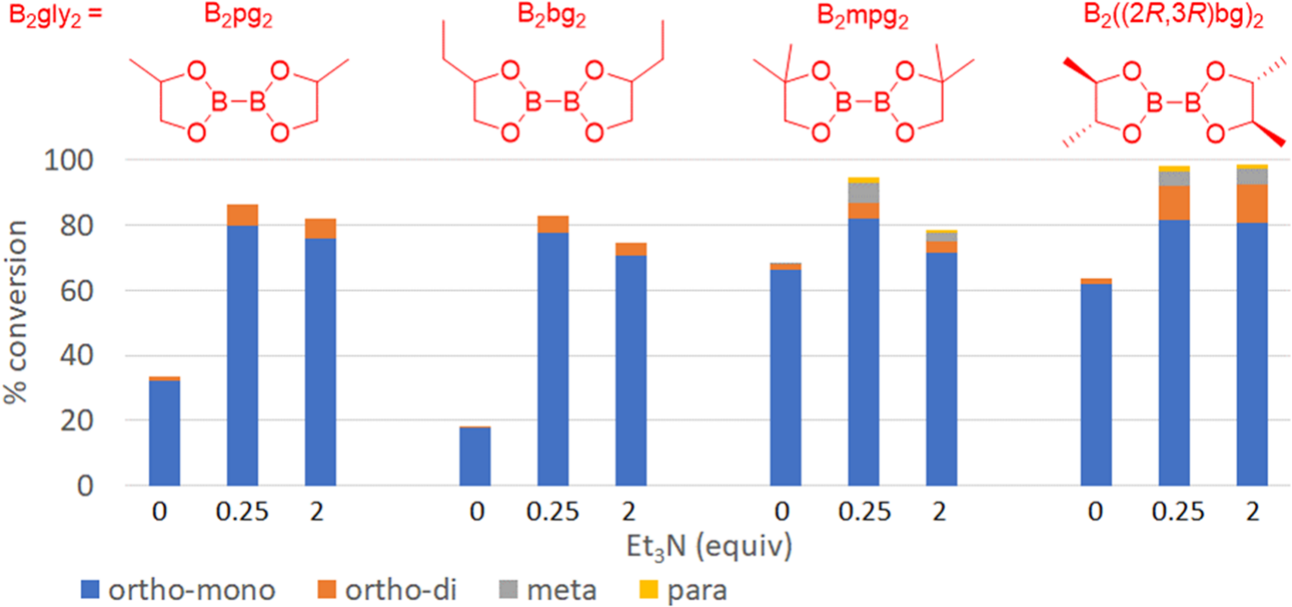
Effect of diboron partner on ortho-CHB of aniline

Whereas ArBeg and ArBpg decomposed, ArBbg and ArBmpg survived product purification by silica gel chromatography. As stated previously, B_2_bg_2_ exhibited higher regioselectivity for the ortho-borylated product compared to B_2_mpg_2_. As a result, B_2_bg_2_ exhibits the best balance of regioselectivity, stability, and reactivity.

Gratifyingly, the high selectivity induced by B_2_bg_2_ and the stability conferred by the Bbg group could be extended to other substituted anilines (Scheme 3). Halide (Cl, Br, I) substituents at the para position were tolerated to make anilines **4**–**6** with good conversions, albeit moderate yields were obtained after isolation. Notably, minor amounts of the 2,6-diborylated byproducts were observed during the synthesis of **4**–**6**. Anilines containing EWG and EDG groups like trifluoromethyl, chloro, methyl, and methoxy at the meta position had no significant adverse effect and yielded **7**–**10** successfully. In the case of 3-fluoroaniline, CHB was observed on both ortho positions, obtaining a mixture of the 2- and 6-borylated aniline **11** in an equal ratio. Small quantities of 2,6-diborylated-3-fluoroaniline were detected, as well during the synthesis of **11**. CHB next to a small substituent as fluorine is not surprising and is commonly observed in iridium-catalyzed borylations.^21,22^ Notably, perfect ortho-CHB regioselectivity was observed for compound **12** in the presence of sterically available C(sp^2^)–H bonds on the phenyl substituent at the meta position. Unfortunately, the reactivity was attenuated in the presence of ortho substituents on the aniline, leading to the isolation of **13** in a low yield. Yan’s group reported a similar limitation in their approach where ortho-substituted anilines did not yield to any product.^14^ Furthermore, attempts to borylate 2,5-substituted anilines did not afford 6-borylated products in any appreciable yield even under forcing conditions (see Supporting Information for details). Notably, neutral alumina was used for the isolation of compoundss **10** and **11**, as deborylation occurred when attempting isolation through silica column chromatography. Overall, this method eliminates the previously required transesterification step with pinacol and is applicable to various substituted anilines.

**Scheme 3:**
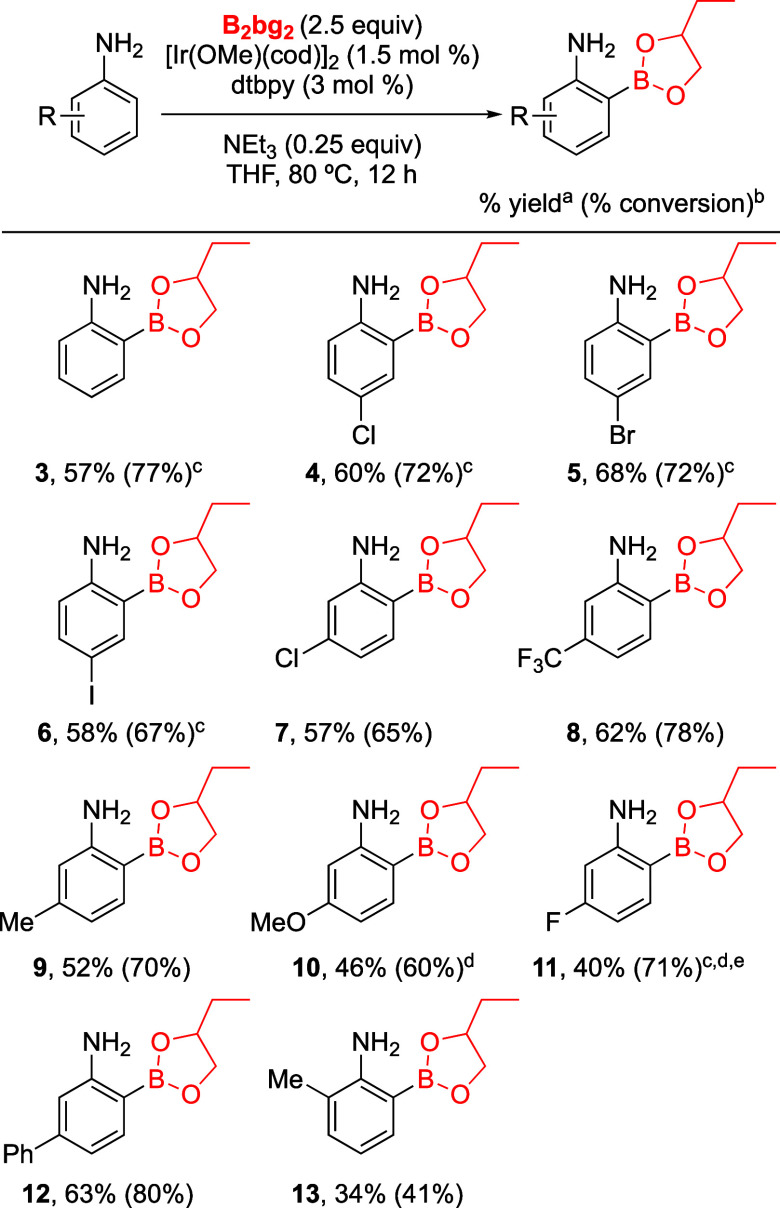
Substrate Scope of Ortho-CHB Aniline Isolated yields reported. Conversions were measured by ^1^H NMR with respect to the remaining starting material. Diborylated product was observed during the reaction. Isolation using neutral alumina column chromatography. Both 2-borylated and 6-borylated aniline products were formed in 1 to 1 ratio.

Upon reconsideration of B_2_pg_2_, we were intrigued by a previous study that demonstrated the slower hydrolysis of ortho-borylated acetamide containing a Beg group compared to the meta or para borylated acetamides.^23^ We asked if the acetamide of **2.1** would impart a similar stability on the Bpg group by blocking any potential side reaction at boron. To test this hypothesis, we synthesized amide **2.3** (Scheme 4), which was found to be a stable crystalline solid even after 1 year, as evidenced by ^1^H NMR analysis. The observed ^11^B NMR chemical shift of 10.3 ppm for the C–Bgly group illustrates notable shielding compared to the typical range of 25–30 ppm for this type of boron. We interpret these data to suggest coordination of the boron atom with the Lewis basic acetamide.

**Scheme 4:**
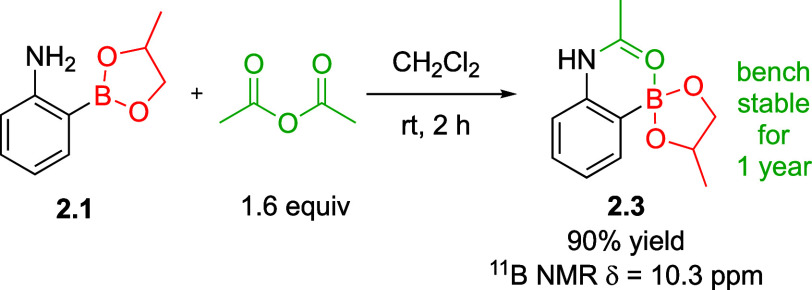
Synthesis of a Stable Ortho Borylated Aniline via Intramolecular Interaction

To summarize, this report shows the high selectivities and other practical benefits that can be achieved by employing diboron reagents not commonly screened during the development of CHB reactions. The use of B_2_bg_2_ as the diboron partner enables ortho-CHB of anilines to proceed with the high regioselectivity observed with B_2_eg_2_ and where the products can be isolated without the need for a transesterification step. Diboron partners that possess a single pendant alkyl group in the glycolate backbone, such as B_2_pg_2_ and B_2_bg_2_, demonstrate excellent regioselectivity in ortho-CHB reactions of aniline. However, the bulkier boron partners, such as B_2_mpg_2_ and B_2_(2*R*,3*R*)bg_2_, negatively impact the regioselectivity. Independent of the boron source, reactions run with 0.25 equiv of triethyl amine gave the best conversions. Deviating from this amount, by using either higher or lower amounts of base, proved to have a negative impact on the formation of ortho-borylated aniline. When subjected to silica gel chromatography ArBeg and ArBpg decompose, but ArBbg and ArBmpg survive. It is possible for undesired reactions to take place during the chromatography, where nucleophilic attack on the boron can occur. Specifically, for the product arising from the ortho-borylation of acetamide with Bpg, a putative intramolecular Lewis acid–base interaction aids in stabilizing the molecule.

## Data Availability

The data underlying this study are available in the published article and its Supporting Information.

## Supporting Information Available

Experimental procedures, including preparation of starting materials, and compound characterization data (PDF)
