# Supplementary material for: Balancing Reactivity, Regioselectivity, and Product Stability in Ir-Catalyzed Ortho-C–H Borylations of Anilines by Modulating the Diboron Partner
Source: Org Lett. 2024 Jun 26;26(26):5420–4. doi: 10.1021/acs.orglett.4c01495 (PMC11232100; doi:10.1021/acs.orglett.4c01495)

Supporting Information

Balancing reactivity, regioselectivity and product stability in Ir-catalyzed ortho C–H borylations of anilines by modulating the diboron partner

Jose R. Montero Bastidas,‡ Anshu Yadav,‡ Seokjoo Lee, Behnaz Ghaffari, Milton R. Smith, III,* and Robert E. Maleczka, Jr.*

Department of Chemistry, Michigan State University, 578 South Shaw Lane, East Lansing, Michigan 48824-1322 USA

Email: maleczka@chemistry.msu.edu

Email: smithmil@msu.edu

Table of Contents

General Information 3

Synthesis of Diboron Partners 4
Synthesis of racemic B_2pg_2 4
Synthesis of (*S,S*)-B_2pg_2 4
Synthesis of B_2bg_2 5
Synthesis of B_2mpg_2 6
Synthesis of $B_2((2R,3R)bg)_2$ 6

Synthesis of a stable ortho borylated aniline with a Bpg group 7
First Step (2.1) 7
Second Step (2.3) 8

CHB of anilines with B_2bg_2 as diboron partner 8
Borylation of aniline with B_2bg_2 (3) 8
Borylation of 4-chloroaniline with B_2bg_2 (4) 9
Borylation of 4-bromoaniline with B_2bg_2 (5) 10
Borylation of 4-iodoaniline with B_2bg_2 (6) 11
Borylation of 3-chloroaniline with B_2bg_2 (7) 12
Borylation of 3-(trifluoromethyl)aniline with B_2bg_2 (8) 12
Borylation of 3-methylaniline with B_2bg_2 (9) 13
Borylation of 3-methoxyaniline with B_2bg_2 (10) 14
Borylation of 3-fluoroaniline with B_2bg_2 (11) 15
Borylation of 3-aminobiphenyl with B_2bg_2 (12) 16
Borylation of 2-methylaniline with B_2bg_2 (13) 17
Borylation of 2-methoxy-5-methylaniline with B_2bg_2 18

REFERENCES: 18

NMR Table of Contents 19

General Information

All commercially available chemicals were used as received unless otherwise indicated. Bis(η^4-1,5-cyclooctadiene)-di-μ-methoxy-diiridium(I) $[Ir(OMe)COD]_2$ was prepared from a well reported procedure in the literature.[1] Tetrahydrofuran (THF) were refluxed over sodium/benzophenone ketyl, distilled and degassed twice before borylation. Column chromatography was performed on flash silica gel (ACME). Thin layer chromatography was performed on 0.25 mm thick aluminum-backed silica gel plates purchased from Merck and visualized with ultraviolet light (λ = 254 nm).

^1H, ^{13}C, and ^{11}B NMR spectra were recorded on Agilent DirectDrive2 (500 MHz for ^1H, 126 MHz for ^{13}C and 160 MHz for ^{11}B). All coupling constants are apparent *J* values measured at the indicated field strengths in Hertz (s = singlet, d = doublet, t = triplet, q = quartet, dd = doublet of doublets, bs = broad singlet, dt = doublet of triplet, td = triplet of doublet, ttt = triplet of triplet of triplet). Spectra taken in $CDCl_3$ were referenced to 7.26 ppm in ^1H NMR and 77.2 ppm in ^{13}C NMR. ^{11}B NMR spectra were referenced to neat $BF_3{\bullet}Et_2O$ as the external standard. ^{13}C NMR resonances for the boron-bearing carbon atom were not observed due to quadrupolar relaxation. High-resolution mass spectra were acquired at the MSU Molecular Metabolism and Disease Mass Spectrometry Core facility using a Waters QTOF Ultima mass spectrometer (ESI) and Leco GC-ToF spectrometer.

Synthesis of Diboron Partners

Diboron partners were synthesized following a reported procedure.[2]

Synthesis of racemic B_2pg_2

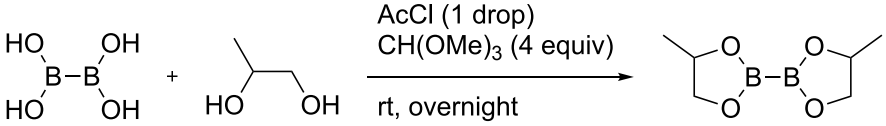

1 equiv 2 equiv B_2pg_2 62% yield

$B_2(OH)_4$ (3.59 g, 40 mmol, 1 equiv) and $CH(OMe)_3$ (16.98 g, 160 mmol, 4 equiv) were stirred in a Schlenk flask and the suspension was degassed with N_2 for 15 minutes. One drop of acetyl chloride, AcCl, was added and the mixture became a homogenous solution. Propylene glycol (pg, 6.09 g, 80 mmol, 2 equiv) was added and the reaction was stirred at room temperature overnight. The mixture was concentrated and then distilled at 74 ºC at 0.1 torr pressure using a short path distillation apparatus to yield 4.20 g of B_2pg_2 as a colorless oil (62% yield) that matched previously reported spectra.[2]

^{1}H NMR (500 MHz, $CDCl_3$) δ 4.62 – 4.51 (m, 2H), 4.26 (dd, J = 9.0, 7.9 Hz, 2H), 3.71 (ddd, J = 9.0, 7.4, 1.0 Hz, 2H), 1.33 (dd, J = 6.2, 0.5 Hz, 6H). ^{13}C NMR{^{1}H} (126 MHz, $CDCl_3$) δ 73.6, 72.1, 21.8. ^{11}B NMR (160 MHz, $CDCl_3$) δ 30.7. HRMS (GC/ToF) *m/z* calc for $C_6H_{12}B_2O_4$ $[M]^+$ 170.0922, found: 170.0911

Synthesis of (*S,S*)-B_2pg_2

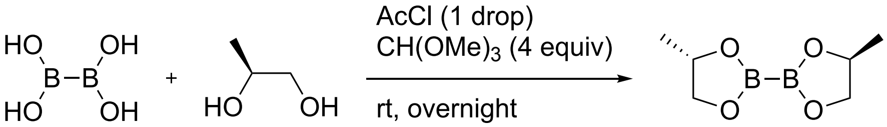

1 equiv 2 equiv (*S,S*)-B_2pg_2 49% yield

$B_2(OH)_4$ (896 mg, 10 mmol, 1 equiv) and $CH(OMe)_3$ (4.24 g, 40 mmol, 4 equiv) were stirred in a Schlenk flask and the suspension was degassed with N_2 for 15 minutes. One drop of acetyl chloride, AcCl, was added and the mixture became a homogenous solution. (*S*)-Propylene glycol ((*S*)-pg, 1.52 g, 20 mmol, 2 equiv) was added and the reaction was stirred at room temperature overnight. The mixture was concentrated and then distilled at 72 ºC at 0.1 torr pressure using a short path distillation apparatus. A solid crashed out on the distilled arm and receiving flask that after recollection yielded 840 mg of (*S,S*)-B_2pg_2 as a white solid (49% yield) with a mp = 152 – 154 ºC that matched previously reported spectra.[2]

^{1}H NMR (500 MHz, $CDCl_3$) δ 4.63 – 4.46 (m, 2H), 4.24 (dd, *J* = 9.0, 7.9 Hz, 2H), 3.67 (dd, *J* = 9.0, 7.4 Hz, 2H), 1.28 (d, *J* = 6.3 Hz, 6H). ^{13}C NMR{^{1}H} (126 MHz, $CDCl_3$) δ 73.5, 72.1, 21.8. ^{11}B NMR (160 MHz, $CDCl_3$) δ 30.7. HRMS (GC/ToF) *m/z* calc for $C_6H_{12}B_2O_4$ $[M]^+$ 170.0922, found: 170.0664; $[\alpha]^{22.66}_{589nm}$ = (+)21.26 (0.1, $CHCl_3$)

Synthesis of B_2bg_2

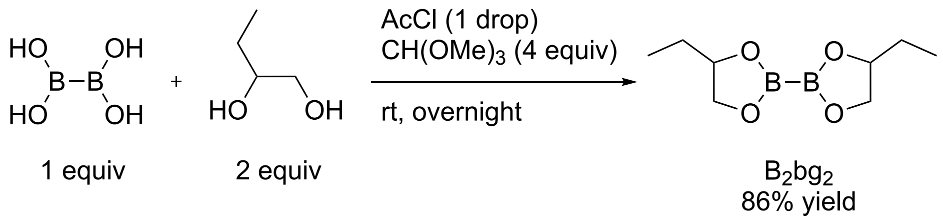

$B_2(OH)_4$ (3.59 g, 40 mmol, 1 equiv) and $CH(OMe)_3$ (16.98 g, 160 mmol, 4 equiv) were stirred in a Schlenk flask and the suspension was degassed with N_2 for 15 minutes. One drop of acetyl chloride, AcCl, was added and the mixture becomes a homogenous solution. Butylene glycol (bg, 7.21 g, 80 mmol, 2 equiv) was added and the reaction wasa stirred at room temperature overnight. The mixture was concentrated and then distilled at 82 ºC at 0.1 torr pressure using a short path distillation apparatus to yield 6.78 g of B_2bg_2 as a colorless oil (86% yield) that matched previously reported spectra.[2]

^{1}H NMR (500 MHz, $CDCl_3$) δ 4.40 – 4.30 (m, 2H), 4.23 (dd, *J* = 9.0, 8.1 Hz, 2H), 3.77 (ddd, *J* = 9.0, 7.3, 1.7 Hz, 2H), 1.73 – 1.50 (m, 4H), 0.94 (t, *J* = 7.4 Hz, 6H). ^{13}C NMR{^{1}H} (126 MHz, $CDCl_3$) δ 78.6, 70.4, 29.0, 9.3. ^{11}B NMR (160 MHz, $CDCl_3$) δ 30.7. HRMS (GC/ToF) *m/*z calc for $C_8H_{16}B_2O_4$ $[M]^+$ 198.1235, found: 198.1224

Synthesis of B₂mpg₂

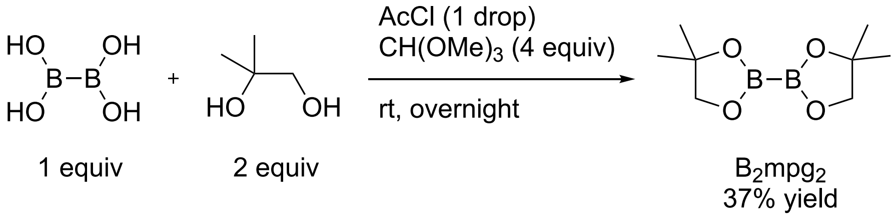

$B_2(OH)_4$ (896 mg, 10 mmol, 1 equiv) and $CH(OMe)_3$ (4.24 g, 40 mmol, 4 equiv) were stirred in a Schlenk flask and the suspension was degassed with N_2 for 15 minutes. One drop of acetyl chloride, AcCl, was added and the mixture becomes a homogenous solution. 2-methyl-1,2-propandiol (mpg, 1.80 g, 20 mmol, 2 equiv) was added and the reaction was stirred at room temperature overnight. The mixture was concentrated and then distilled at 78 ºC at 0.1 torr pressure using a short path distillation apparatus to yield 730 mg of B_2mpg_2 as a colorless oil (37% yield) that matched previously reported spectra.[2]

^{1}H NMR (500 MHz, $CDCl_3$) δ 3.89 (s, 4H), 1.36 (s, 12H). ^{13}C NMR{^{1}H} (126 MHz, $CDCl_3$) δ 80.5, 77.3, 28.6. ^{11}B NMR (160 MHz, $CDCl_3$) δ 30.8. GC-MS (EI) *m/*z calcd for $C_8H_{16}B_2O_4$ [M] 198.1, found: 198.1

Synthesis of B₂((2*R*,3*R*)bg)₂

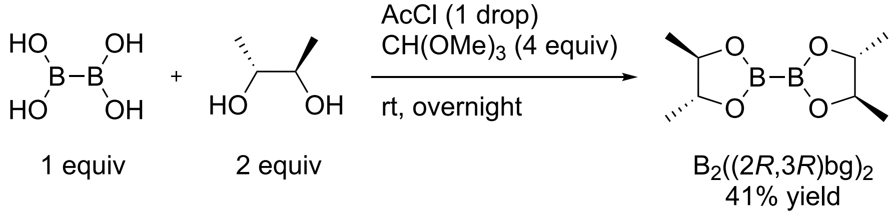

$B_2(OH)_4$ (896 mg, 10 mmol, 1 equiv) and $CH(OMe)_3$ (4.24 g, 40 mmol, 4 equiv) were stirred in a Schlenk flask and the suspension was degassed with N_2 for 15 minutes. One drop of acetyl chloride, AcCl, was added and the mixture becomes a homogenous solution. (2*R*,3*R*)-butanediol ((2*R*,3*R*)bg, 1.80 g, 20 mmol, 2 equiv) was added and the reaction was stirred at room temperature overnight. The mixture was concentrated and then distilled at 80 ºC at 0.1 torr pressure using a short path distillation apparatus to yield 820 mg of B_2(2*R*,3*R*)bg_2 as a colorless oil (41% yield) that matched previously reported spectra.[2]

^{1}H NMR (500 MHz, $CDCl_3$) δ 4.00 (qd, *J* = 4.0, 2.1 Hz, 4H), 1.33 – 1.22 (m, 12H). $^{13}C\{^1H\}$ NMR (126 MHz, $CDCl_3$) δ 80.3, 20.9. ^{11}B NMR (160 MHz, $CDCl_3$) δ 30.5. GC-MS (EI) *m/z* calcd for $C_8H_{16}B_2O_4$ [M] 198.1, found: 198.1; $[\alpha]^{22.66}_{589nm}$ = (-)26.64 (0.1, CH_2Cl_2)

Synthesis of a stable ortho borylated aniline with a Bpg group

First Step (2.1)

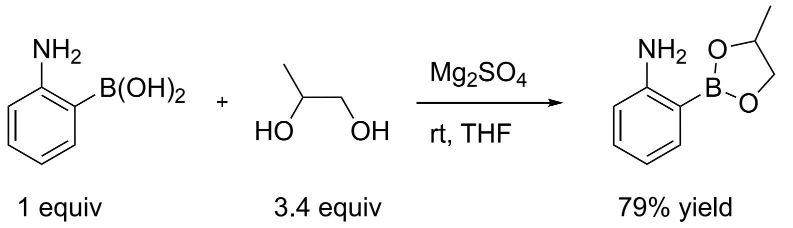

2-Aminophenyl boronic acid (274 mg, 2 mmol, 1 equiv), 1,2-propandiol (517 mg, 6.8 mmol, 3.4 equiv), Mg_2SO_4 anhydrous (100 mg) and THF (1.6 mL) were placed in a round bottom flask and stirred overnight at room temperature. After 12 h, the mixture was filtered, and the liquid concentrated. The residue was purified by column chromatography with silica gel (hexane: ethyl acetate = 4: 1 as eluent) to remove the excess of diol. The fractions containing product were collected and concentrated to give 139 mg of **2.1** as a colorless oil (79% yield).

^{1}H NMR (500 MHz, $CDCl_3$) δ 7.62 (dd, *J* = 7.5, 1.7 Hz, 1H), 7.23 (ddd, *J* = 8.2, 7.2, 1.7 Hz, 1H), 6.69 (td, *J* = 7.3, 1.0 Hz, 1H), 6.61 (dd, *J* = 8.2 Hz, 0.9 Hz, 1H), 4.84 – 4.53 (m, 3H), 4.44 (dd, *J*

= 8.8, 7.6 Hz, 1H), 3.88 (dd, J = 8.8, 7.2 Hz, 1H), 1.41 (d, J = 6.2 Hz, 3H). ^{13}C NMR{^{1}H} (126 MHz, $CDCl_3$) δ 153.7, 136.9, 133.1, 117.1, 115.0, 73.6, 72.2, 21.9. ^{11}B NMR (160 MHz, $CDCl_3$) δ 31.4. HRMS (ESI) *m/z* calcd for $C_9H_{13}BNO_2$ $[M+H]^+$ 178.1039, found: 178.1043.

Second Step (2.3)

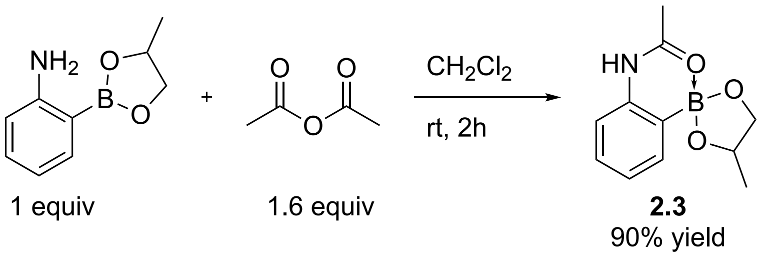

In a 20 mL vial, **2.1** (177 mg, 1 mmol, 1 equiv) was stirred with acetic anhydride (0.15 mL, 1.6 mmol, 1.6 equiv) in DCM (2.7 mL) at room temperature. After 2 h, the mixture was concentrated under reduce pressure and diethyl ether was added to crashed out a solid. The white solid was filtrated to yield 197 mg of **2.3** (90% yield, mp 192.5-194.5 °C).

^{1}H NMR (500 MHz, $CDCl_3$) δ 12.39 (bs, 1H), 7.59 (dd, J = 6.9, 2.0 Hz, 1H), 7.17 (m, 2H), 6.96 (d, J = 7.5 Hz, 1H), 4.57 – 4.49 (m, 1H), 4.27 (dd, J = 8.3, 6.1 Hz, 1H), 3.70 (t, J = 7.9 Hz, 1H), 1.64 (s, 3H), 1.34 (d, J = 6.1 Hz, 3H). ^{13}C NMR{^{1}H} (126 MHz, $CDCl_3$) δ 169.6, 137.9, 133.2, 128.6, 126.7, 116.2, 71.7, 71.5, 21.1, 20.6. ^{11}B NMR (160 MHz, $CDCl_3$) δ 11.0. HRMS (ESI) *m/z* calc for $C_{11}H_{15}BNO_3$ $[M+H]^+$ 220.1147, found 220.1152 $^{\nu}$CO = 1650 cm^{-1}

CHB of anilines with B_2bg_2 as diboron partner

Borylation of aniline with B_2bg_2 (3)

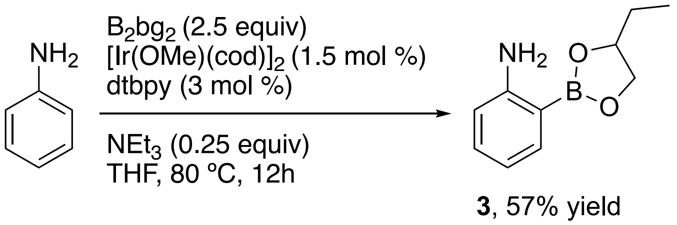

In a nitrogen filled glove box, a 5.0 mL conical vial was charged with $[Ir(cod)(OMe)]_2$ (10 mg, 1.5 mol %), dtbpy (8 mg, 3.0 mol %), B_2bg_2 (495 mg, 2.5 mmol, 2.5 equiv), aniline (93 mg, 1.0 mmol, 1 equiv) and Et_3N (0.04 mL, 0.25 mmol, 0.25 equiv) in dry THF (3.0 mL). The vial was capped with a teflon pressure cap, taken out of the glove box and stirred into a pre-heated aluminum block at 80 °C. After 12 h, the mixture was concentrated under reduced pressure and purified by gradient column chromatography with silica gel (hexane/ethyl acetate 95:05 → hexane/ ethyl acetate 90:10). The fractions containing product were collected to yield 109 mg of **3** as a thick oil that contained ~ 7% of aniline according to ^1H NMR (57% yield of **3**).

^1H NMR (500 MHz, $CDCl_3$) δ 7.62 (dd, *J* = 7.5, 1.7 Hz, 1H), 7.23 (ddd, *J* = 8.2, 7.2, 1.7 Hz, 1H), 6.69 (ddd, *J* = 7.2, 7.5, 1.0 Hz, 1H), 6.61 (dd, *J* = 8.2, 1.0 Hz, 1H), 4.71 (bs, 2H), 4.52 (dtd, *J* = 7.8, 6.9, 5.8 Hz, 1H), 4.40 (dd, *J* = 8.8, 7.8 Hz, 1H), 3.95 (dd, *J* = 8.8, 6.9 Hz, 1H), 1.80 – 1.61 (m, 2H), 1.01 (t, *J* = 7.4 Hz, 3H). $^{13}C\{^1H\}$ NMR (126 MHz, $CDCl_3$) δ 153.7, 137.0, 133.1, 117.2, 115.1, 78.5, 70.6, 29.1, 9.4. ^{11}B NMR (160 MHz, $CDCl_3$) δ 31.5. HRMS (ESI) *m/z* calc for $C_{10}H_{15}BNO_2$ $[M+H]^+$ 192.1198, found 192.1202

Borylation of 4-chloroaniline with B_2bg_2 (4)

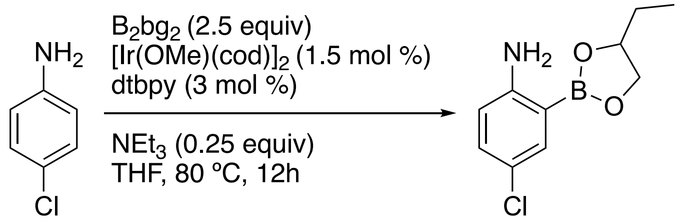

4, 60% yield

In a nitrogen filled glove box, a 5.0 mL conical vial was charged with $[Ir(cod)(OMe)]_2$ (10 mg, 1.5 mol %), dtbpy (8 mg, 3.0 mol %), B_2bg_2 (495 mg, 2.5 mmol, 2.5 equiv), 4-chloroaniline (127 mg, 1.0 mmol, 1 equiv) and Et_3N (0.04 mL, 0.25 mmol, 0.25 equiv) in dry THF (3.0 mL). The vial was capped with a teflon pressure cap, taken out of the glove box and stirred into a pre-

heated aluminum block at 80 °C. After 12 h, the mixture was concentrated under reduced pressure and purified by gradient column chromatography with silica gel (hexane/ethyl acetate 95:05 → hexane/ethyl acetate 90:10). The fractions containing product were collected to yield 135 mg of **4** as a thick oil that contained ~ 5% of 4-chloroaniline according to ^{1}H NMR (60% yield of **4**).

^{1}H NMR (500 MHz, $CDCl_3$) δ 7.55 (d, J = 2.6 Hz, 1H), 7.15 (dd, J = 8.6, 2.6 Hz, 1H), 6.54 (d, J = 8.6 Hz, 1H), 4.71 (bs, 2H), 4.52 (dtd, J = 7.8, 7.0, 5.7 Hz, 1H), 4.41 (dd, J = 8.9, 7.8 Hz, 1H), 3.95 (dd, J = 8.9, 7.0 Hz, 1H), 1.78 – 1.61 (m, 2H), 1.01 (t, J = 7.4 Hz, 3H). ^{13}C NMR{^{1}H} (126 MHz, $CDCl_3$) δ 152.2, 136.1, 132.8, 121.8, 116.5, 78.8, 70.7, 29.1, 9.3. ^{11}B NMR (160 MHz, $CDCl_3$) δ 31.0. HRMS (ESI) *m/z* calc for $C_{10}H_{14}BClNO_2$ $[M+H]^+$ 226.0808, found 226.0811

Borylation of 4-bromoaniline with B_2bg_2 (5)

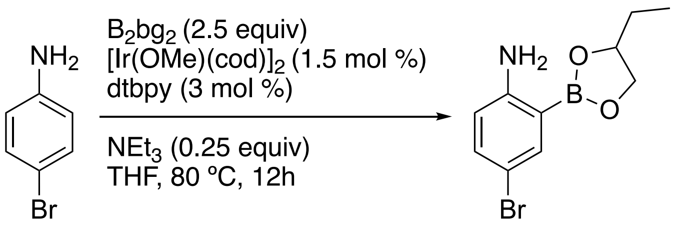

5, 68% yield

In a nitrogen filled glove box, a 5.0 mL conical vial was charged with $[Ir(cod)(OMe)]_2$ (10 mg, 1.5 mol %), dtbpy (8 mg, 3.0 mol %), B_2bg_2 (495 mg, 2.5 mmol, 2.5 equiv), 4-bromoaniline (172 mg, 1.0 mmol, 1 equiv) and Et_3N (0.04 mL, 0.25 mmol, 0.25 equiv) in dry THF (3.0 mL). The vial was capped with a teflon pressure cap, taken out of the glove box and stirred into a pre-heated aluminum block at 80 °C. After 12 h, the mixture was concentrated under reduced pressure and purified by gradient column chromatography with silica gel (hexane/ethyl acetate 95:05 → hexane/ethyl acetate 90:10). The fractions containing product were collected to yield 183 mg of **5** as a thick oil (68% yield).

^{1}H NMR (500 MHz, $CDCl_3$) δ 7.69 (d, *J* = 2.5 Hz, 1H), 7.28 (dd, *J* = 8.6, 2.5 Hz, 1H), 6.49 (d, *J* = 8.6 Hz, 1H), 4.72 (bs, 2H), 4.52 (dtd, *J* = 7.8, 7.0, 5.7 Hz, 1H), 4.41 (dd, *J* = 8.9, 7.8 Hz, 1H), 3.95 (dd, *J* = 8.9, 7.0 Hz, 1H), 1.80 – 1.61 (m, 2H), 1.01 (t, *J* = 7.4 Hz, 3H). ^{13}C{^{1}H} NMR (126 MHz, $CDCl_3$) δ 152.6, 139.0, 135.6, 116.9, 108.9, 78.8, 70.7, 29.1, 9.3. ^{11}B NMR (160 MHz, $CDCl_3$) δ 30.9. HRMS (ESI) *m/z* calc for $C_{10}H_{14}BBrNO_2$ $[M+H]^+$ 270.0295, found 270.0293

Borylation of 4-iodoaniline with B_2bg_2 (6)

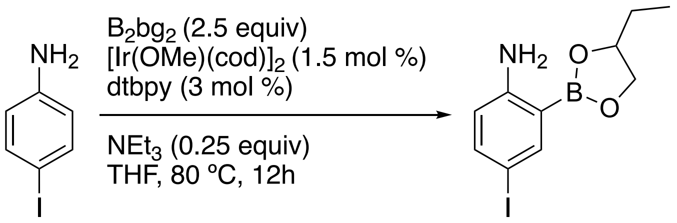

6, 58% yield

In a nitrogen filled glove box, a 5.0 mL conical vial was charged with $[Ir(cod)(OMe)]_2$ (10 mg, 1.5 mol %), dtbpy (8 mg, 3.0 mol %), B_2bg_2 (495 mg, 2.5 mmol, 2.5 equiv), 4-iodoaniline (219 mg, 1.0 mmol, 1 equiv) and Et_3N (0.04 mL, 0.25 mmol, 0.25 equiv) in dry THF (3.0 mL). The vial was capped with a teflon pressure cap, taken out of the glove box and stirred into a pre-heated aluminum block at 80 °C. After 12 h, the mixture was concentrated under reduced pressure and purified by gradient column chromatography with silica gel (hexane/ethyl acetate 95:05 → hexane/ethyl acetate 90:10). The fractions containing product were collected to yield 183 mg of **6** as a thick oil (58% yield).

^{1}H NMR (500 MHz, $CDCl_3$) δ 7.86 (d, *J* = 2.3 Hz, 1H), 7.44 (dd, *J* = 8.5, 2.3 Hz, 1H), 6.41 (d, *J* = 8.5 Hz, 1H), 4.51 (ddd, *J* = 7.8, 7.0, 5.7 Hz, 1H), 4.40 (dd, *J* = 8.9, 7.8 Hz, 1H), 4.18 (bs, 2H), 3.94 (dd, *J* = 8.9, 7.0 Hz, 1H), 1.80 – 1.60 (m, 2H), 1.01 (t, *J* = 7.4 Hz, 3H). ^{13}C{^{1}H} NMR (126 MHz, $CDCl_3$) δ 153.0, 145.1, 141.3, 138.1, 117.5, 78.7, 70.7, 29.1, 9.3. ^{11}B NMR (160 MHz, $CDCl_3$) δ 30.7. HRMS (ESI) *m/z* calc for $C_{10}H_{14}BINO_2$ $[M+H]^+$ 318.0157, found 318.0164

Borylation of 3-chloroaniline with B_2bg_2 (7)

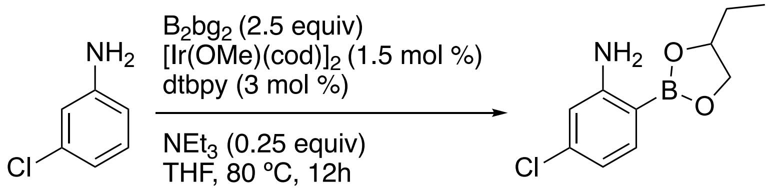

7, 57% yield

In a nitrogen filled glove box, a 5.0 mL conical vial was charged with $[Ir(cod)(OMe)]_2$ (10 mg, 1.5 mol %), dtbpy (8 mg, 3.0 mol %), B_2bg_2 (495 mg, 2.5 mmol, 2.5 equiv), 3-chloroaniline (127 mg, 1.0 mmol, 1 equiv) and Et_3N (0.04 mL, 0.25 mmol, 0.25 equiv) in dry THF (3.0 mL). The vial was capped with a teflon pressure cap, taken out of the glove box and stirred into a pre-heated aluminum block at 80 °C. After 12 h, the mixture was concentrated under reduced pressure and purified by gradient column chromatography with silica gel (hexane/ethyl acetate 95:5 → hexane/ethyl acetate 90:10). The fractions containing product were collected to yield 128 mg of **7** as a thick oil (57% yield).

^{1}H NMR (500 MHz, $CDCl_3$) δ 7.52 (d, *J* = 8.0 Hz, 1H), 6.64 (dd, *J* = 8.0, 1.9 Hz, 1H), 6.60 (d, *J* = 1.9 Hz, 1H), 4.79 (bs, 2H), 4.51 (ddd, *J* = 7.8, 7.0, 5.7 Hz, 1H), 4.40 (dd, *J* = 8.9, 7.8 Hz, 1H), 3.94 (dd, *J* = 8.8, 7.0 Hz, 1H), 1.76 – 1.62 (m, 2H), 1.01 (t, *J* = 7.4 Hz, 3H). ^{13}C{^{1}H} NMR (126 MHz, $CDCl_3$) δ 154.7, 138.9, 138.3, 117.4, 114.6, 78.7, 70.6, 29.1, 9.4. ^{11}B NMR (160 MHz, $CDCl_3$) δ 31.1. HRMS (ESI) *m/z* calc for $C_{10}H_{14}BClNO_2$ $[M+H]^+$ 226.0808, found 226.0811

Borylation of 3-(trifluoromethyl)aniline with B_2bg_2 (8)

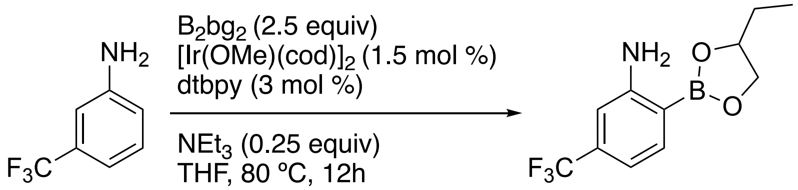

8, 62% yield

In a nitrogen filled glove box, a 5.0 mL conical vial was charged with $[Ir(cod)(OMe)]_2$ (10 mg, 1.5 mol %), dtbpy (8 mg, 3.0 mol %), B_2bg_2 (495 mg, 2.5 mmol, 2.5 equiv), 3-(trifluoromethyl)aniline (161 mg, 1.0 mmol, 1 equiv) and Et_3N (0.04 mL, 0.25 mmol, 0.25 equiv) in dry THF (3.0 mL). The vial was capped with a teflon pressure cap, taken out of the glove box and stirred into a pre-heated aluminum block at 80 °C. After 12 h, the mixture was concentrated under reduced pressure and purified by gradient column chromatography with silica gel (hexane/ethyl acetate 95:5 → hexane/ethyl acetate 90:10). The fractions containing product were collected to yield 160 mg of **8** as a thick oil that contained ~ 8% of unknown trace impurity according to ^1H NMR (62% yield of **8**).

^1H NMR (500 MHz, $CDCl_3$) δ 7.70 (d, J = 7.8 Hz, 1H), 6.88 (dd, J = 7.8, 1.6 Hz, 1H), 6.81 (d, J = 1.6 Hz, 1H), 4.90 (bs, 2H), 4.54 (ddd, J = 7.8, 7.0, 5.7 Hz, 1H), 4.43 (dd, J = 8.9, 7.8 Hz, 1H), 3.97 (dd, J = 8.9, 7.0 Hz, 1H), 1.79 – 1.64 (m, 2H), 1.02 (t, J = 7.4 Hz, 3H). $^{13}C\{^1H\}$ NMR (126 MHz, $CDCl_3$) δ 153.7, 137.7, 134.6 (q, J = 31.7 Hz), 124.2 (q, J = 272.6 Hz), 113.1 (q, J = 3.8 Hz), 111.2 (q, J = 3.9 Hz), 78.8, 70.7, 29.1, 9.3. ^{11}B NMR (160 MHz, $CDCl_3$) δ 31.1. ^{19}F NMR (470 MHz, CDCl3) δ 63.6 Hz. HRMS (ESI) *m/z* calc for $C_{11}H_{14}BF_3NO_2$ $[M+H]^+$ 260.1072, found 260.1078

Borylation of 3-methylaniline with B_2bg_2 (9)

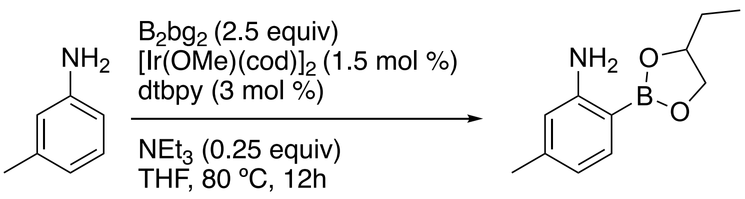

9, 52% yield

In a nitrogen filled glove box, a 5.0 mL conical vial was charged with $[Ir(cod)(OMe)]_2$ (10 mg, 1.5 mol %), dtbpy (8 mg, 3.0 mol %), B_2bg_2 (495 mg, 2.5 mmol, 2.5 equiv), 3-methylaniline (107 mg, 1.0 mmol, 1 equiv) and Et_3N (0.04 mL, 0.25 mmol, 0.25 equiv) in dry THF (3.0 mL).

The vial was capped with a teflon pressure cap, taken out of the glove box and stirred into a pre-heated aluminum block at 80 °C. After 12 h, the mixture was concentrated under reduced pressure and purified by gradient column chromatography with silica gel (hexane/ethyl acetate 95:5 → hexane/ethyl acetate 90:10). The fractions containing product were collected to yield 106 mg of **9** as a thick oil that contained ~ 12% of unknown trace impurity according to ^{1}H NMR (52% yield of **9**).

^{1}H NMR (500 MHz, $CDCl_3$) δ 7.51 (d, J = 7.6 Hz, 1H), 6.52 (d, J = 7.6 Hz, 1H), 6.41 (s, 1H), 4.66 (bs, 2H), 4.50 (ddd, J = 7.8, 6.8, 5.7 Hz, 1H), 4.39 (dd, J = 8.8, 7.8 Hz, 1H), 3.93 (dd, J = 8.9, 6.9 Hz, 1H), 2.25 (s, 3H), 1.75 – 1.63 (m, 2H), 1.0 (t, J = 7.4 Hz, 3H). ^{13}C NMR{^{1}H} (126 MHz, $CDCl_3$) δ 153.9, 143.5, 137.0, 118.5, 115.6, 78.4, 70.5, 29.1, 21.9, 9.4. ^{11}B NMR (160 MHz, $CDCl_3$) δ 31.4. HRMS (ESI) *m/z* calc for $C_{11}H_{17}BNO_2$ $[M+H]^+$ 206.1354, found 206.1357

Borylation of 3-methoxyaniline with B_2bg_2 (10)

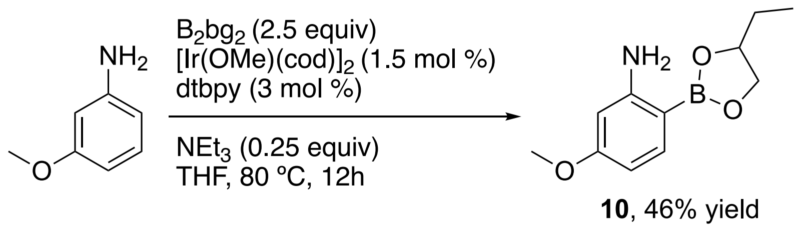

In a nitrogen filled glove box, a 5.0 mL conical vial was charged with $[Ir(cod)(OMe)]_2$ (10 mg, 1.5 mol %), dtbpy (8 mg, 3.0 mol %), B_2bg_2 (495 mg, 2.5 mmol, 2.5 equiv), 3-methoxyaniline (123 mg, 1.0 mmol, 1 equiv) and Et_3N (0.04 mL, 0.25 mmol, 0.25 equiv) in dry THF (3.0 mL). The vial was capped with a teflon pressure cap, taken out of the glove box and stirred into a pre-heated aluminum block at 80 °C. After 12 h, the mixture was concentrated under reduced pressure and purified by gradient column chromatography with neutral alumina gel (hexane/ethyl acetate 90:10 → hexane/ethyl acetate 80:20). The fractions containing product were collected to yield 102

mg of **10** as a thick oil that contained ~ 7% of unknown trace impurity according to ^{1}H NMR (46% yield of **10**).

^{1}H NMR (500 MHz, $CDCl_3$) δ 7.54 (d, *J* = 8.3 Hz, 1H), 6.28 (dd, *J* = 8.4, 2.3 Hz, 1H), 6.13 (d, *J* = 2.2 Hz, 1H), 4.74 (bs, 2H), 4.48 (m, 1H), 4.37 (ddd, *J* = 8.8, 7.7, 1.1 Hz, 1H), 3.91 (dd, *J* = 8.9, 7.0 Hz, 1H), 3.77 (s, 3H), 1.76 – 1.61 (m, 2H), 1.0 (t, *J* = 7.4 Hz, 3H). ^{13}C{^{1}H} NMR (126 MHz, $CDCl_3$) δ 163.9, 155.6, 138.6, 104.1, 99.6, 78.4, 70.4, 55.1, 29.1, 9.4. ^{11}B NMR (160 MHz, $CDCl_3$) δ 31.1. HRMS (ESI) *m/z* calc for $C_{11}H_{16}BNO_3$ $[M+H]^+$ 222.1296, found 222.1304

Borylation of 3-fluoroaniline with B_2bg_2 (11)

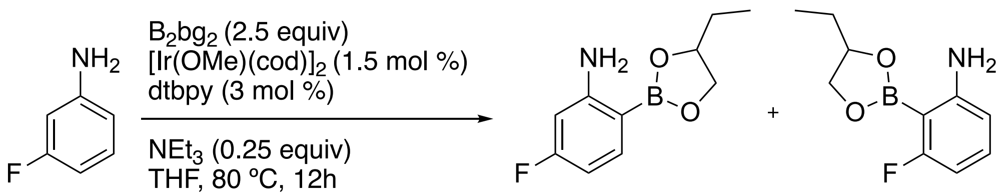

11.1 **11.2**

In a nitrogen filled glove box, a 5.0 mL conical vial was charged with $[Ir(cod)(OMe)]_2$ (10 mg, 1.5 mol %), dtbpy (8 mg, 3.0 mol %), B_2bg_2 (495 mg, 2.5 mmol, 2.5 equiv), 3-fluoroaniline (111 mg, 1.0 mmol, 1 equiv) and Et_3N (0.04 mL, 0.25 mmol, 0.25 equiv) in dry THF (3.0 mL). The vial was capped with a teflon pressure cap, taken out of the glove box and stirred into a pre-heated aluminum block at 80 °C. After 12 h, the reaction was stopped, and the mixture was concentrated under reduced pressure.

First attempt at purification. The residue was purified by gradient column chromatography with silica gel (hexane/ethyl acetate 95:05 → hexane/ethyl acetate 90:10). The fractions containing product were collected to yield 31 mg of **11.1** as a thick oil with traces of dtbpy and borate byproducts (15% yield).

NMR characterization of **11.1**: ^{1}H NMR (500 MHz, $CDCl_3$) δ 7.58 (dd, *J* = 8.3, 7.3 Hz, 1H), 6.38 (td, *J* = 8.5, 2.3 Hz, 1H), 6.28 (dd, *J* = 11.5, 2.3 Hz, 1H), 4.84 (bs, 2H), 4.54 – 4.46 (m, 1H), 4.39

(dd, J = 8.8, 7.8 Hz, 1H), 3.93 (dd, J = 8.9, 7.0 Hz, 1H), 1.78 – 1.62 (m, 2H), 1.01 (t, J = 7.4 Hz, 4H). $^{13}C\{^1H\}$ NMR (126 MHz, $CDCl_3$) δ 166.6 (d, J = 248.3 Hz), 155.9 (d, J = 11.4 Hz), 139.2 (d, J = 10.7 Hz), 104.7 (d, J = 20.9 Hz), 101.4 (d, J = 23.6 Hz), 78.6, 70.6, 29.1, 9.4. ^{11}B NMR (160 MHz, $CDCl_3$) δ 30.8. ^{19}F NMR (470 MHz, CDCl3) δ -107.9 (dt, J = 11.4, 7.9 Hz). HRMS (ESI) *m/z* calc for $C_{10}H_{14}BFNO_2$ $[M+H]^+$ 210.1104, found 210.1102

Second attempt at purification. The reaction was repeated with similar conditions as described above. In this case, the residue was purified by gradient column chromatography with neutral alumina gel (hexane/ethyl acetate 90:10 → hexane/ethyl acetate 80:20) to yield 83 mg of a mixture of **11.1** and **11.2** as a thick oil (40% yield).

NMR characterization of **11.2**: ^1H NMR (500 MHz, $CDCl_3$) δ 7.14 (td, J = 8.1, 6.6 Hz, 1H), 6.41 – 6.36 (m, 1H), 6.34 (ddd, J = 9.9, 8.0, 0.9 Hz, 1H), 4.96 (bs, 2H), 4.58 – 4.47 (m, 1H), 4.45 – 4.40 (m, 1H), 3.97 (dd, J = 8.9, 7.0 Hz, 1H), 1.80 – 1.60 (m, 2H), 1.01 (t, J = 7.4 Hz, 3H). $^{13}C\{^1H\}$ NMR (126 MHz, $CDCl_3$) δ 169.4 (d, J = 248.8 Hz), 155.6 (d, J = 10.9 Hz), 134.0 (d, J = 11.9 Hz), 110.8 (d, J = 2.8 Hz), 103.6 (d, J = 24.9 Hz), 78.3, 70.3, 29.0, 9.3. ^{11}B NMR (160 MHz, $CDCl_3$) δ 30.8. ^{19}F NMR (470 MHz, CDCl3) δ -101.4 (dd, J = 9.9, 6.6 Hz). HRMS (ESI) *m/z* calc for $C_{10}H_{14}BFNO_2$ $[M+H]^+$ 210.1104, found 210.1102

Borylation of 3-aminobiphenyl with B_2bg_2 (12)

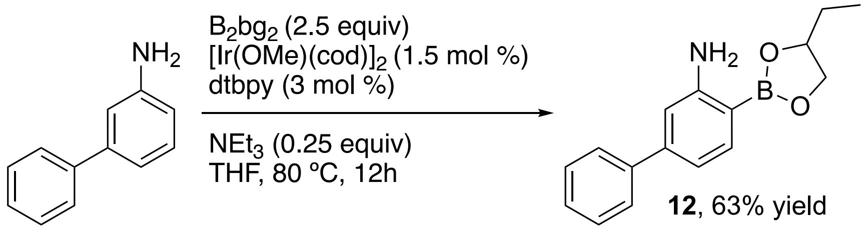

In a nitrogen filled glove box, a 5.0 mL conical vial was charged with $[Ir(cod)(OMe)]_2$ (10 mg, 1.5 mol %), dtbpy (8 mg, 3.0 mol %), B_2bg_2 (495 mg, 2.5 mmol, 2.5 equiv), 3-aminobiphenyl (169 mg, 1.0 mmol, 1 equiv) and Et_3N (0.04 mL, 0.25 mmol, 0.25 equiv) in dry THF (3.0 mL).

The vial was capped with a teflon pressure cap, taken out of the glove box and stirred into a pre-heated aluminum block at 80 °C. After 12 h, the mixture was concentrated under reduced pressure and purified by gradient column chromatography with silica gel (hexane/ethyl acetate 95:5 → hexane/ethyl acetate 90:10). The fractions containing product were collected to yield 168 mg of **12** as a thick oil that contained ~ 7% of [1,1'-biphenyl]-3-amine according to ^{1}H NMR (63% yield of **12**).

^{1}H NMR (500 MHz, $CDCl_3$) δ 7.69 (d, J = 7.7 Hz, 1H), 7.59 (m, 2H), 7.42 (m, 2H), 7.34 (m, 1H), 6.94 (dd, J = 7.7, 1.6 Hz, 1H), 6.84 (d, J = 1.6 Hz, 1H), 4.80 (bs, 2H), 4.54 (dtd, J = 7.8, 6.8, 5.7 Hz, 1H), 4.43 (dd, J = 8.8, 7.8 Hz, 1H), 3.97 (dd, J = 8.8, 6.9 Hz, 1H), 1.79 – 1.64 (m, 2H), 1.03 (t, J = 7.4 Hz, 3H). ^{13}C NMR{^{1}H} (126 MHz, $CDCl_3$) δ 154.1, 145.8, 141.3, 137.5, 128.8, 127.7, 127.3, 116.4, 113.6, 78.6, 70.6, 29.1, 9.4. ^{11}B NMR (160 MHz, $CDCl_3$) δ 30.8. HRMS (ESI) *m/z* calc for $C_{16}H_{18}BNO_2$ $[M+H]^+$ 268.1512, found 268.1518

Borylation of 2-methylaniline with B_2bg_2 (13)

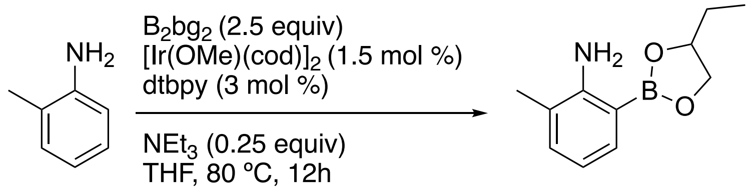

13, 34% yield

In a nitrogen filled glove box, a 5.0 mL conical vial was charged with $[Ir(cod)(OMe)]_2$ (10 mg, 1.5 mol %), dtbpy (8 mg, 3.0 mol %), B_2bg_2 (495 mg, 2.5 mmol, 2.5 equiv), 2-methylaniline (107 mg, 1.0 mmol, 1 equiv) and Et_3N (0.04 mL, 0.25 mmol, 0.25 equiv) in dry THF (3.0 mL). The vial was capped with a teflon pressure cap, taken out of the glove box and stirred into a pre-heated aluminum block at 80 °C. After 12 h, the mixture was concentrated under reduced pressure and purified by gradient column chromatography with silica gel (hexane/ethyl acetate 95:5 → hexane/ethyl acetate 90:10). The fractions containing product were collected to yield 69 mg of **13**

as a thick oil that contained ~ 8% of unknown trace impurity according to ^{1}H NMR (34% yield of **13**).

^{1}H NMR (500 MHz, $CDCl_3$) δ 7.53 (d, *J* = 7.6 Hz, 1H), 7.14 (d, *J* = 6.6 Hz, 1H), 6.65 (t, *J* = 7.4 Hz, 1H), 4.74 (bs, 2H), 4.53 (m, 1H), 4.41 (dd, *J* = 8.9, 7.8 Hz, 1H), 3.95 (dd, *J* = 8.8, 6.9 Hz, 1H), 2.14 (s, 3H), 1.78 – 1.64 (m, 2H), 1.02 (t, *J* = 7.4 Hz, 3H). ^{13}C{^{1}H} NMR (126 MHz, $CDCl_3$) δ 152.0, 134.9, 134.1, 121.7, 117.1, 78.6, 70.5, 29.1, 17.8, 9.4. ^{11}B NMR (160 MHz, $CDCl_3$) δ 31.5. HRMS (ESI) *m/z* calc for $C_{11}H_{17}BNO_2$ $[M+H]^+$ 206.1354, found 206.1353

Borylation of 2-methoxy-5-methylaniline with B_2bg_2

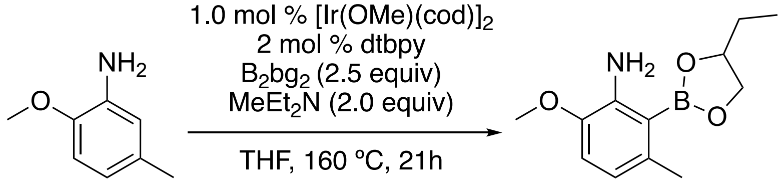

In a nitrogen filled glove box, a 5.0 mL conical vial was charged with $[Ir(cod)(OMe)]_2$ (10 mg, 1.5 mol %), dtbpy (8 mg, 3.0 mol %), B_2bg_2 (495 mg, 2.5 mmol, 2.5 equiv), 2-methoxy-5-methylaniline (137 mg, 1.0 mmol, 1 equiv) and Et_3N (0.04 mL, 0.25 mmol, 0.25 equiv) in dry THF (3.0 mL). The vial was capped with a teflon pressure cap, taken out of the glove box and stirred into a pre-heated aluminum block at 160 °C. After 21 h, the mixture was concentrated under reduced pressure. ^{1}H-NMR of the crude reaction product indicated 10 % conversion to 6-Bbg-3-methoxy-5-methylaniline (i.e. 6-methoxy-3-methyl-2-(4-methyl-1,3,2-dioxaborolan-2-yl)aniline).

REFERENCES

(1) Uson, R.; Oro, L. A.; Cabeza, J. A.; Bryndza, H. E.; Stepro, M. P. Dinuclear Methoxy, Cyclooctadiene, and Barrelene Complexes of Rhodium(I) and Iridium(I). In *Inorganic Syntheses*; John Wiley & Sons, Inc.: Hoboken, NJ, USA, 2007; pp 126–130.

(2) Fornwald, R. M.; Yadav, A.; Montero Bastidas, J. R.; Smith, M. R., III; Maleczka, R. E., Jr. Simple and Green Preparation of Tetraalkoxydiborons and Diboron Diolates from tetrahydroxydiboron *J. Org. Chem.* **2024**, *89,* (Accepted for publication April 05, 2024).

NMR Table of Contents

^{1}H NMR of racemic B_2pg_2 ($CDCl_3$, 500 MHz) 22
^{13}C NMR of racemic B_2pg_2 ($CDCl_3$, 126 MHz) 23
^{11}B NMR of racemic B_2pg_2 ($CDCl_3$, 160 MHz) 24
^{1}H NMR of (*S*)-B_2pg_2 ($CDCl_3$, 500 MHz) 25
^{13}C NMR of (*S*)-B_2pg_2 ($CDCl_3$, 126 MHz) 26
^{11}B NMR of (*S*)-B_2pg_2 ($CDCl_3$, 160 MHz) 27
^{1}H NMR of B_2bg_2 ($CDCl_3$, 500 MHz) 28
^{13}C NMR of B_2bg_2 ($CDCl_3$, 126 MHz) 29
^{11}B NMR of B_2bg_2 ($CDCl_3$, 160 MHz) 30
^{1}H NMR of B_2mpg_2 ($CDCl_3$, 500 MHz) 31
^{13}C NMR of B_2mpg_2 ($CDCl_3$, 126 MHz) 32
^{11}B NMR of B_2mpg_2 ($CDCl_3$, 160 MHz) 33
^{1}H NMR of $B_2((2R,3R)bg)_2$ ($CDCl_3$, 500 MHz) 34
^{13}C NMR of $B_2((2R,3R)bg)_2$ ($CDCl_3$, 126 MHz) 35
^{11}B NMR of $B_2((2R,3R)bg)_2$ ($CDCl_3$, 160 MHz) 36
^{1}H NMR of *ortho* Bpg-borylated aniline (2.1) ($CDCl_3$, 500 MHz) 37
^{11}B NMR of *ortho* Bpg-borylated aniline (2.1) ($CDCl_3$, 160 MHz) 38
^{1}H NMR of *ortho* Bpg-borylated phenylacetamide (2.3) ($CDCl_3$, 500 MHz) 40
^{13}C NMR of *ortho* Bpg-borylated phenylacetamide (2.3) ($CDCl_3$, 160 MHz) 41
^{11}B NMR of *ortho* Bpg-borylated phenylacetamide (2.3) ($CDCl_3$, 126 MHz) 42
^{1}H NMR spectrum of the reaction mixture of CHB aniline (3) ($CDCl_3$, 500 MHz) 43
^{1}H NMR of *ortho* Bbg-borylated aniline (3) ($CDCl_3$, 500 MHz) 44
^{13}C NMR of *ortho* Bbg-borylated aniline (3) ($CDCl_3$, 160 MHz) 45
^{11}B NMR of *ortho* Bbg-borylated aniline (3) ($CDCl_3$, 126 MHz) 46
^{1}H NMR spectrum of the reaction mixture of CHB 4-chloroaniline (4) ($CDCl_3$, 500 MHz) 47
^{1}H NMR of *ortho* Bbg-borylated 4-chloroaniline (4) ($CDCl_3$, 500 MHz) 48
^{13}C NMR of *ortho* Bbg-borylated 4-chloroaniline (4) ($CDCl_3$, 160 MHz) 49
^{11}B NMR of *ortho* Bbg-borylated 4-chloroaniline (4) ($CDCl_3$, 126 MHz) 50

^{1}H NMR spectrum of the reaction mixture of CHB 4-bromoaniline (5) ($CDCl_3$, 500 MHz)....... 51
^{1}H NMR of *ortho* Bbg-borylated 4-bromoaniline (5) ($CDCl_3$, 500 MHz) 52
^{13}C NMR of *ortho* Bbg-borylated 4-bromoaniline (5) ($CDCl_3$, 160 MHz) 53
^{11}B NMR of *ortho* Bbg-borylated 4-bromoaniline (5) ($CDCl_3$, 126 MHz) 54
^{1}H NMR spectrum of the reaction mixture of CHB 4-iodoaniline (6) ($CDCl_3$, 500 MHz).......... 55
^{1}H NMR of *ortho* Bbg-borylated 4-iodoaniline (6) ($CDCl_3$, 500 MHz)..................................... 56
^{13}C NMR of *ortho* Bbg-borylated 4-iodoaniline (6) ($CDCl_3$, 160 MHz) 57
^{11}B NMR of *ortho* Bbg-borylated 4-iodoaniline (6) ($CDCl_3$, 126 MHz) 58
^{1}H NMR spectrum of the reaction mixture of CHB 3-chloroaniline (7) ($CDCl_3$, 500 MHz)....... 59
^{1}H NMR of *ortho* Bbg-borylated 3-chloroaniline (7) ($CDCl_3$, 500 MHz) 60
^{13}C NMR of *ortho* Bbg-borylated 3-chloroaniline (7) ($CDCl_3$, 160 MHz) 61
^{11}B NMR of *ortho* Bbg-borylated 3-chloroaniline (7) ($CDCl_3$, 126 MHz) 62
^{1}H NMR spectrum of the reaction mixture of CHB 3-(trifluoromethyl)aniline (8) ($CDCl_3$, 500 MHz) 63
^{1}H NMR of *ortho* Bbg-borylated 3-(trifluoromethyl)aniline (8) ($CDCl_3$, 500 MHz).................. 64
^{13}C NMR of *ortho* Bbg-borylated 3-(trifluoromethyl)aniline (8) ($CDCl_3$, 160 MHz) 65
^{11}B NMR of *ortho* Bbg-borylated 3-(trifluoromethyl)aniline (8) ($CDCl_3$, 126 MHz) 66
^{1}H NMR spectrum of the reaction mixture of CHB 3-methylaniline (9) ($CDCl_3$, 500 MHz)...... 68
^{1}H NMR of *ortho* Bbg-borylated 3-methylaniline (9) ($CDCl_3$, 500 MHz).................................. 69
^{13}C NMR of *ortho* Bbg-borylated 3-methylaniline (9) ($CDCl_3$, 160 MHz) 70
^{11}B NMR of *ortho* Bbg-borylated 3-methylaniline (9) ($CDCl_3$, 126 MHz) 71
^{1}H NMR spectrum of the reaction mixture of CHB 3-methoxyaniline (10) ($CDCl_3$, 500 MHz). 72
^{1}H NMR of *ortho* Bbg-borylated 3-methoxyaniline (10) ($CDCl_3$, 500 MHz)............................. 73
^{13}C NMR of *ortho* Bbg-borylated 3-methoxyaniline (10) ($CDCl_3$, 160 MHz)............................ 74
^{11}B NMR of *ortho* Bbg-borylated 3-methoxyaniline (10) ($CDCl_3$, 126 MHz)............................ 75
^{1}H NMR spectrum of the reaction mixture of CHB 3-fluoroaniline (11) ($CDCl_3$, 500 MHz) 76
^{1}H NMR of *ortho* Bbg-borylated 3-fluoroaniline (11.1) ($CDCl_3$, 500 MHz) (Isolation done using silica gel) 77
^{1}H NMR of *ortho* Bbg-borylated 3-fluoroaniline (11.1 + 11.2) ($CDCl_3$, 500 MHz) (Isolation done using neutral alumina gel) 78

^{1}H NMR of *ortho* Bbg-borylated 3-fluoroaniline (11) ($CDCl_3$, 500 MHz) (Stacked NMR spectrums of reaction mixture, and products isolated by neutral alumina gel and silica gel respectively) 79
^{13}C NMR of *ortho* Bbg-borylated 3-fluoroaniline (11.1 + 11.2) ($CDCl_3$, 500 MHz) (Isolation done using neutral alumina gel) 80
^{11}B NMR of *ortho* Bbg-borylated 3-fluoroaniline (11.1 + 11.2) ($CDCl_3$, 500 MHz) (Isolation done using neutral alumina gel) 81
^{1}H NMR spectrum of the reaction mixture of CHB 3-aminobiphenyl (12) ($CDCl_3$, 500 MHz).. 83
^{1}H NMR of *ortho* Bbg-borylated 3-aminobiphenyl (12) ($CDCl_3$, 500 MHz).......... 84
^{13}C NMR of *ortho* Bbg-borylated 3-aminobiphenyl (12) ($CDCl_3$, 160 MHz) 85
^{11}B NMR of *ortho* Bbg-borylated 3-aminobiphenyl (12) ($CDCl_3$, 126 MHz) 86
^{1}H NMR spectrum of the reaction mixture of CHB 2-methylaniline (13) ($CDCl_3$, 500 MHz).... 87
^{1}H NMR of *ortho* Bbg-borylated 2-methylaniline (13) ($CDCl_3$, 500 MHz).......... 88
^{13}C NMR of *ortho* Bbg-borylated 2-methylaniline (13) ($CDCl_3$, 160 MHz) 89
^{11}B NMR of *ortho* Bbg-borylated 2-methylaniline (13) ($CDCl_3$, 126 MHz) 90

^{1}H NMR of racemic B_2pg_2 ($CDCl_3$, 500 MHz)

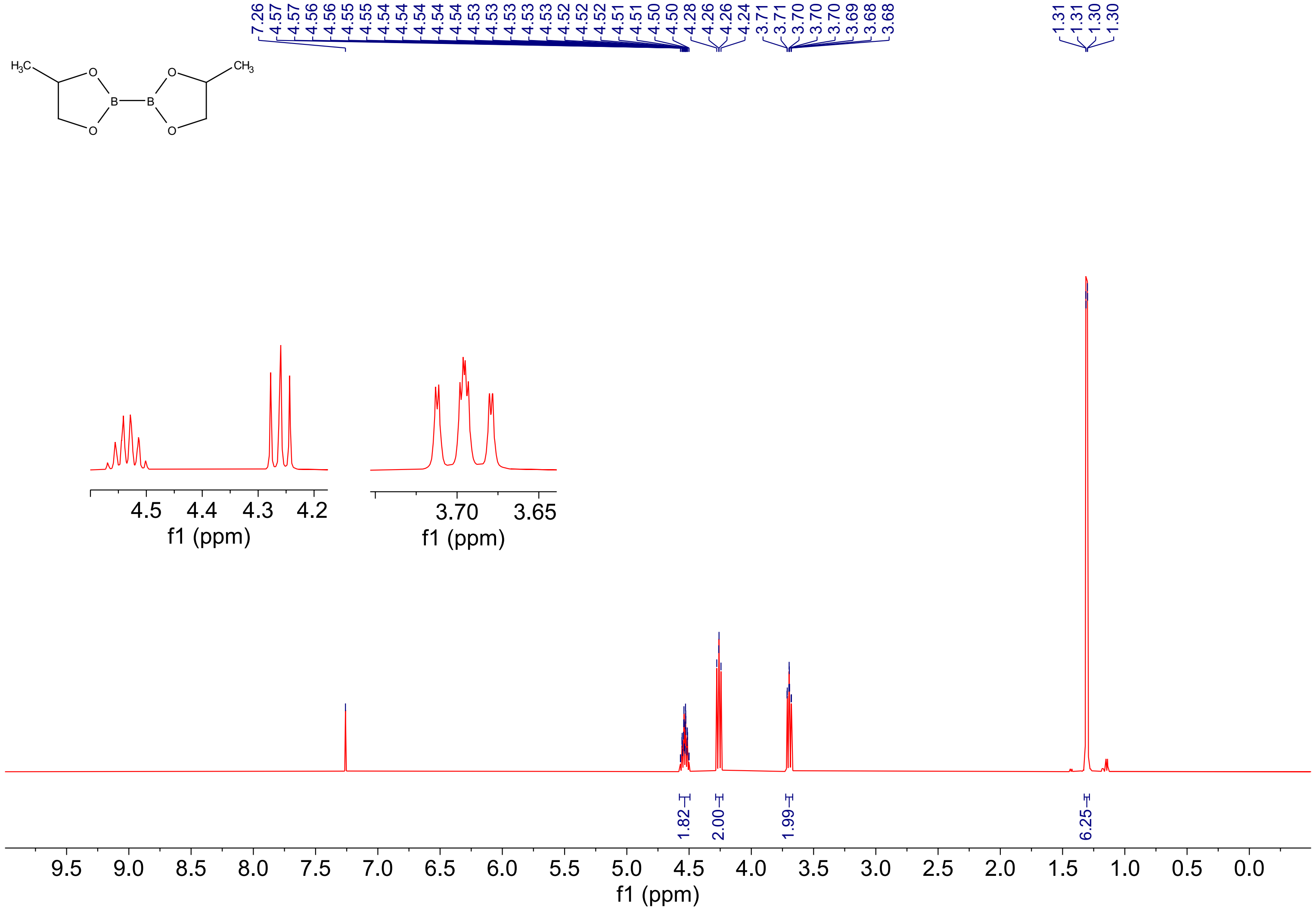

$^{13}C\{^{1}H\}$ NMR of racemic B_2pg_2 ($CDCl_3$, 126 MHz)

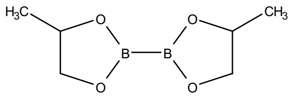

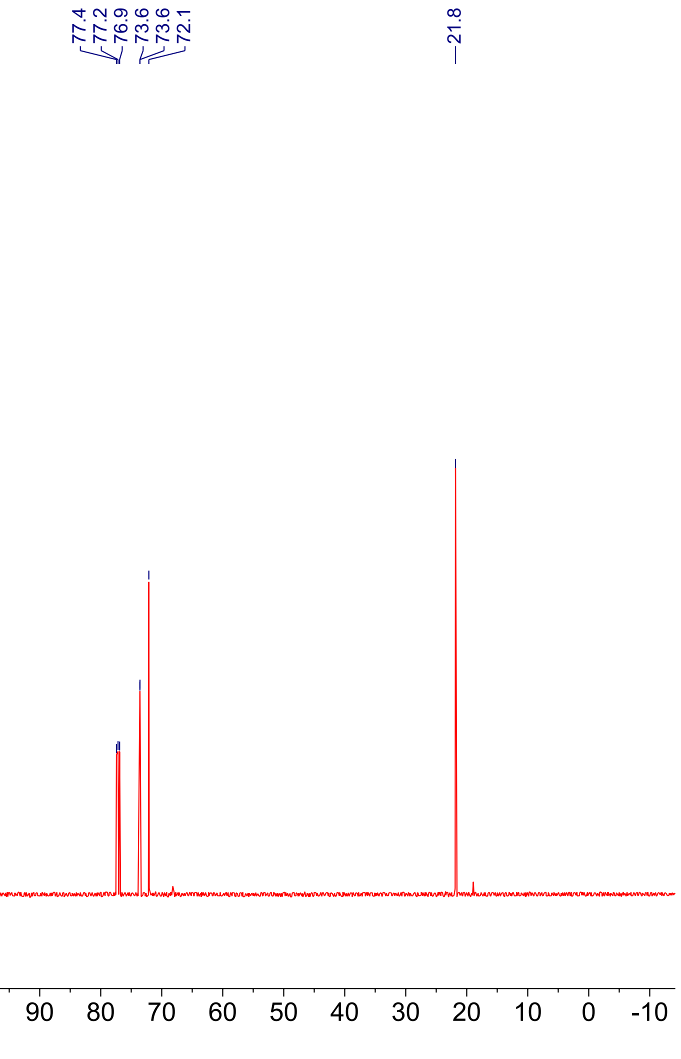

^{11}B NMR of racemic B_2pg_2 ($CDCl_3$, 160 MHz)

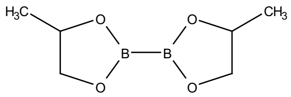

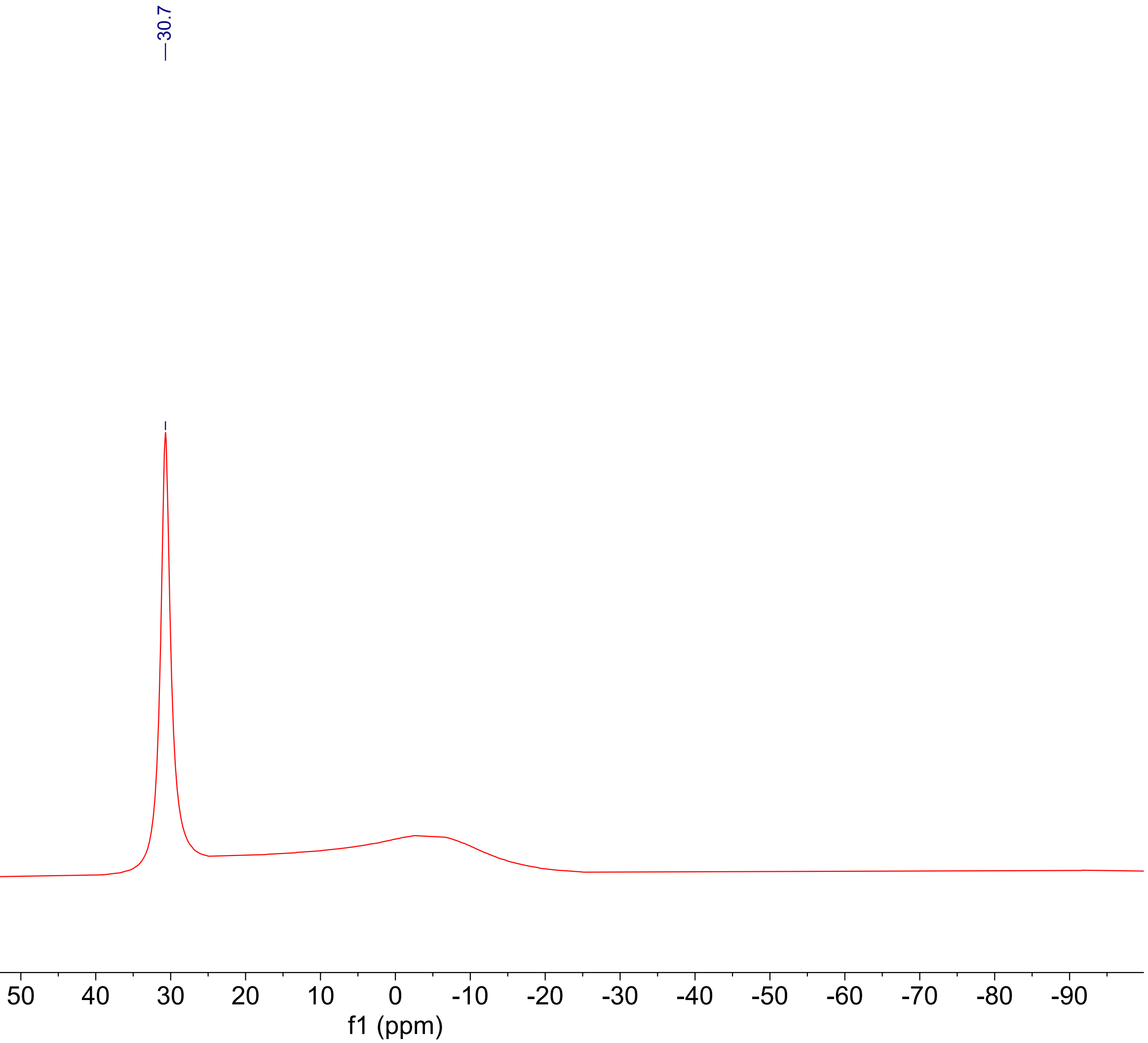

^{1}H NMR of (*S*)-B_2pg_2 ($CDCl_3$, 500 MHz)

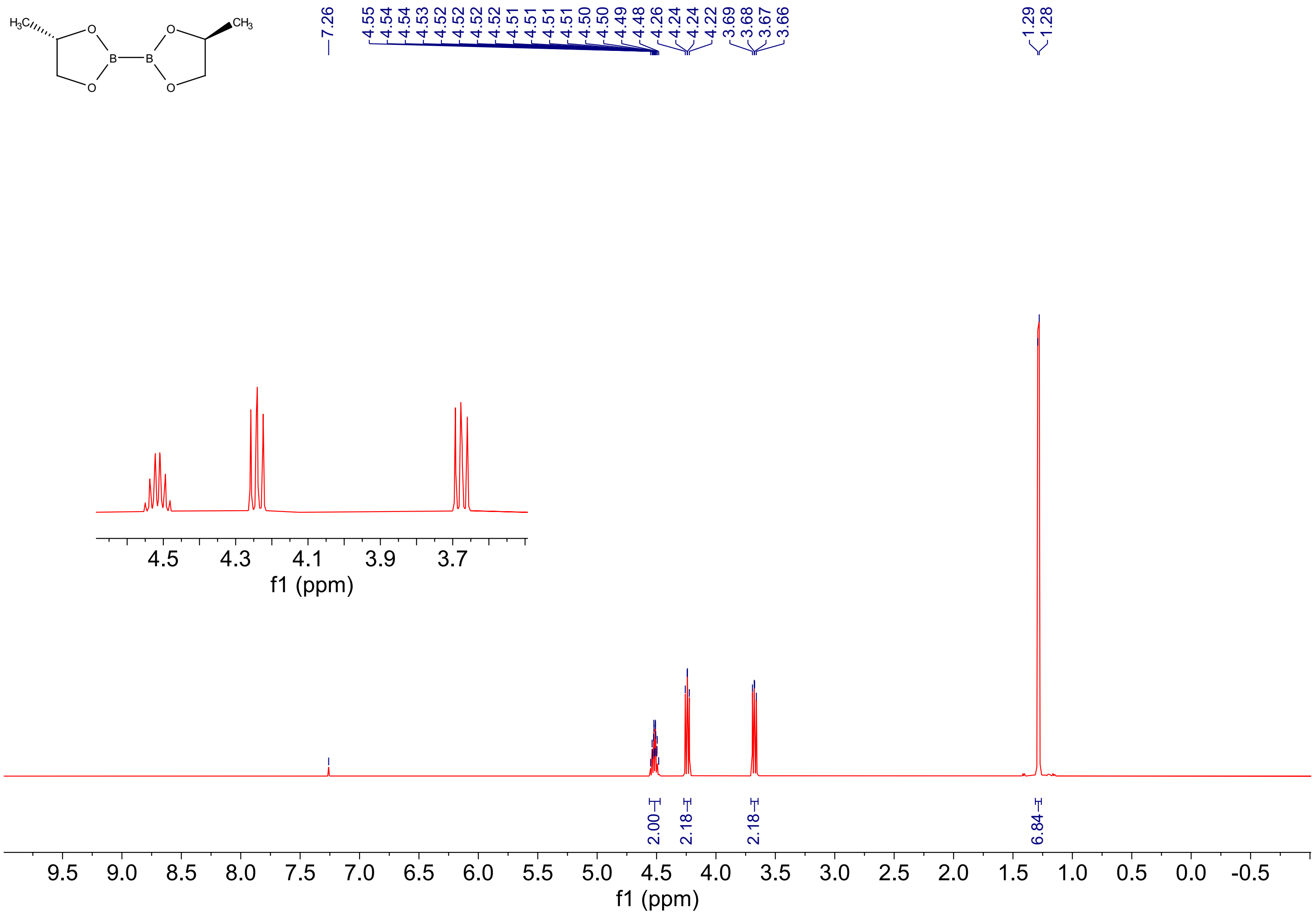

$^{13}C\{^1H\}$ NMR of (*S*)-B_2pg_2 ($CDCl_3$, 126 MHz)

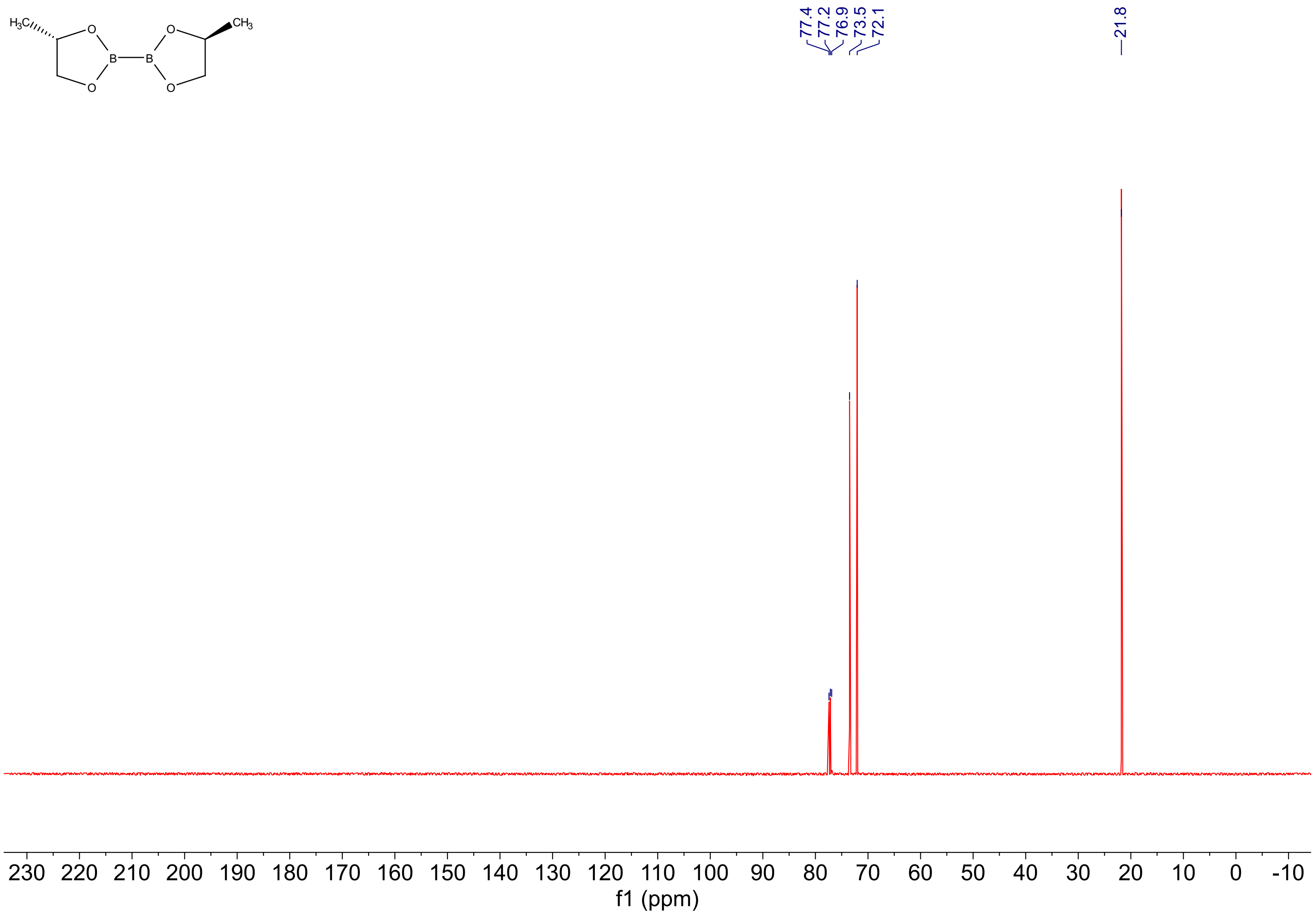

^{11}B NMR of (*S*)-B_2pg_2 ($CDCl_3$, 160 MHz)

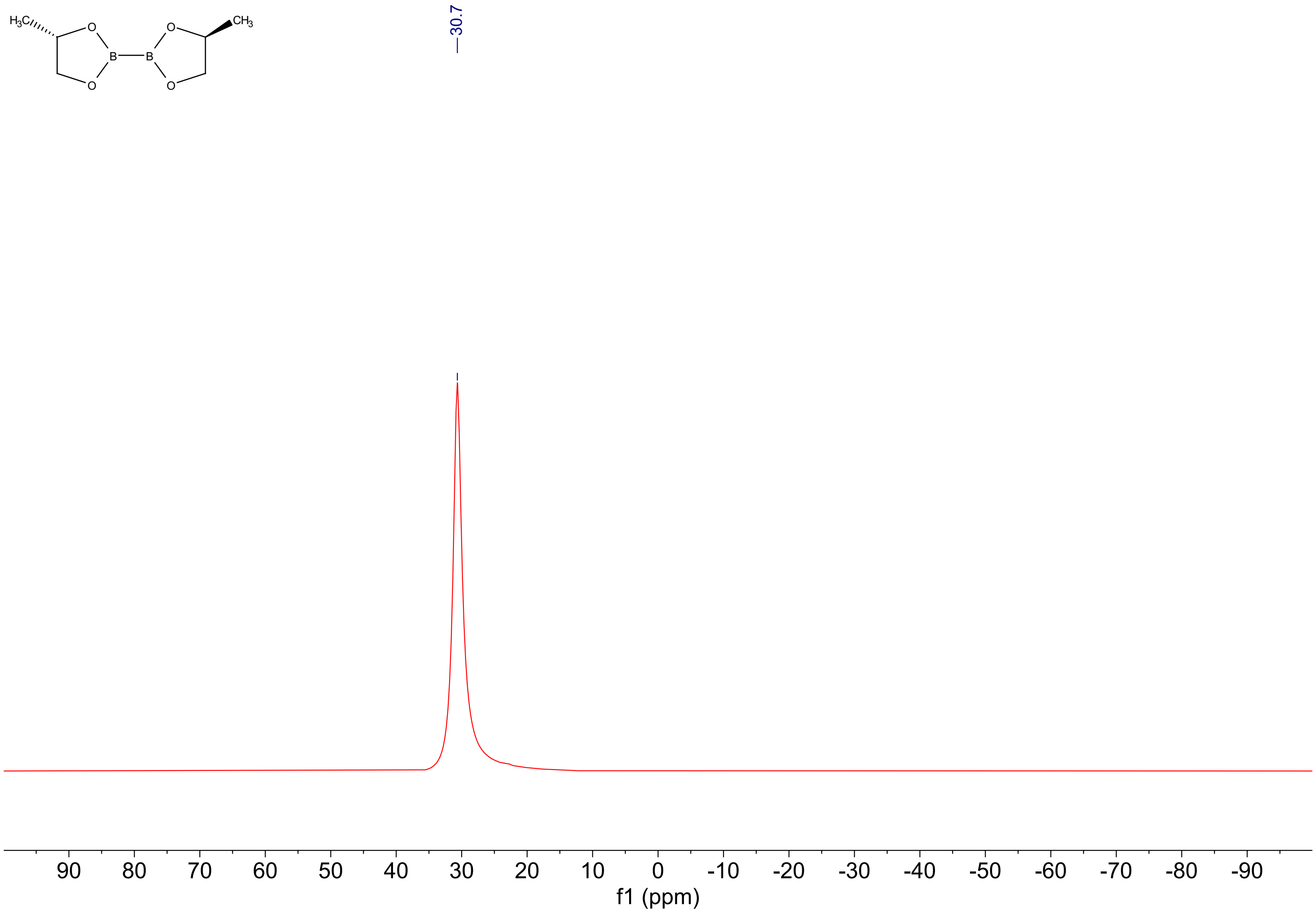

^{1}H NMR of B_2bg_2 ($CDCl_3$, 500 MHz)

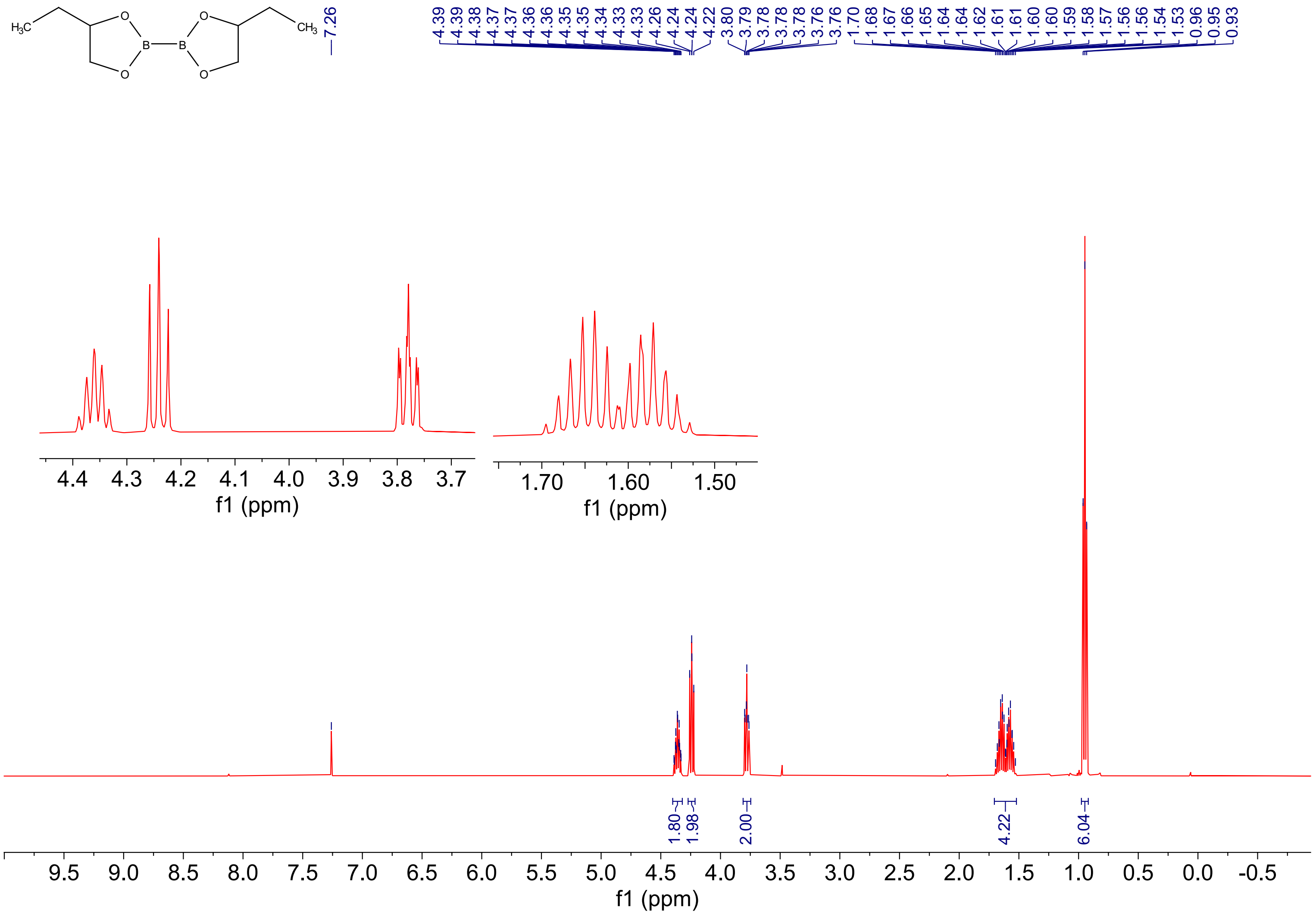

$^{13}C\{^1H\}$ NMR of B_2bg_2 ($CDCl_3$, 126 MHz)

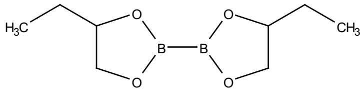

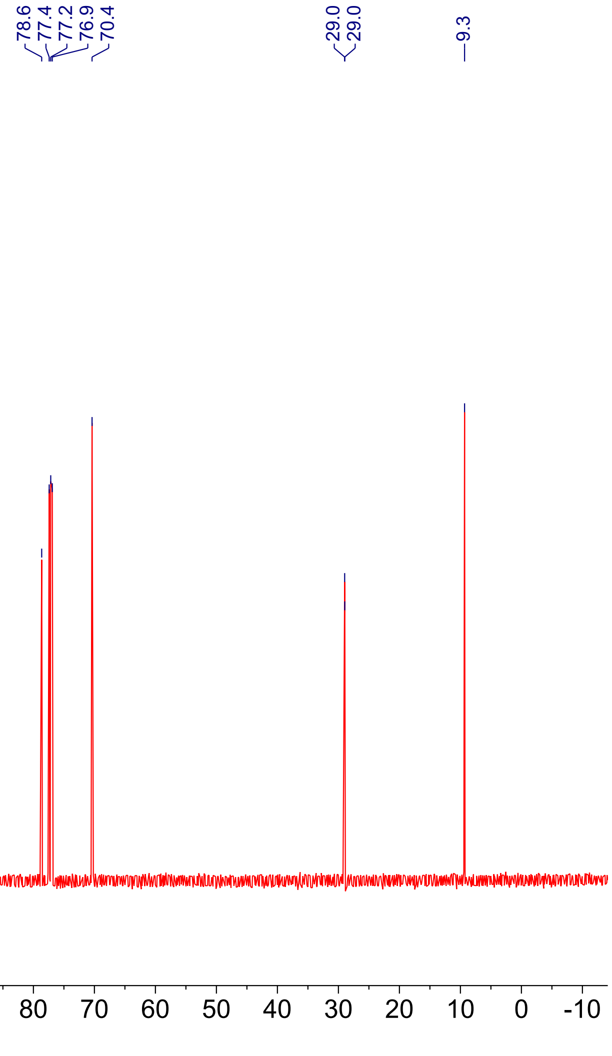

^{11}B NMR of B_2bg_2 ($CDCl_3$, 160 MHz)

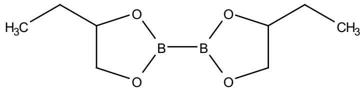

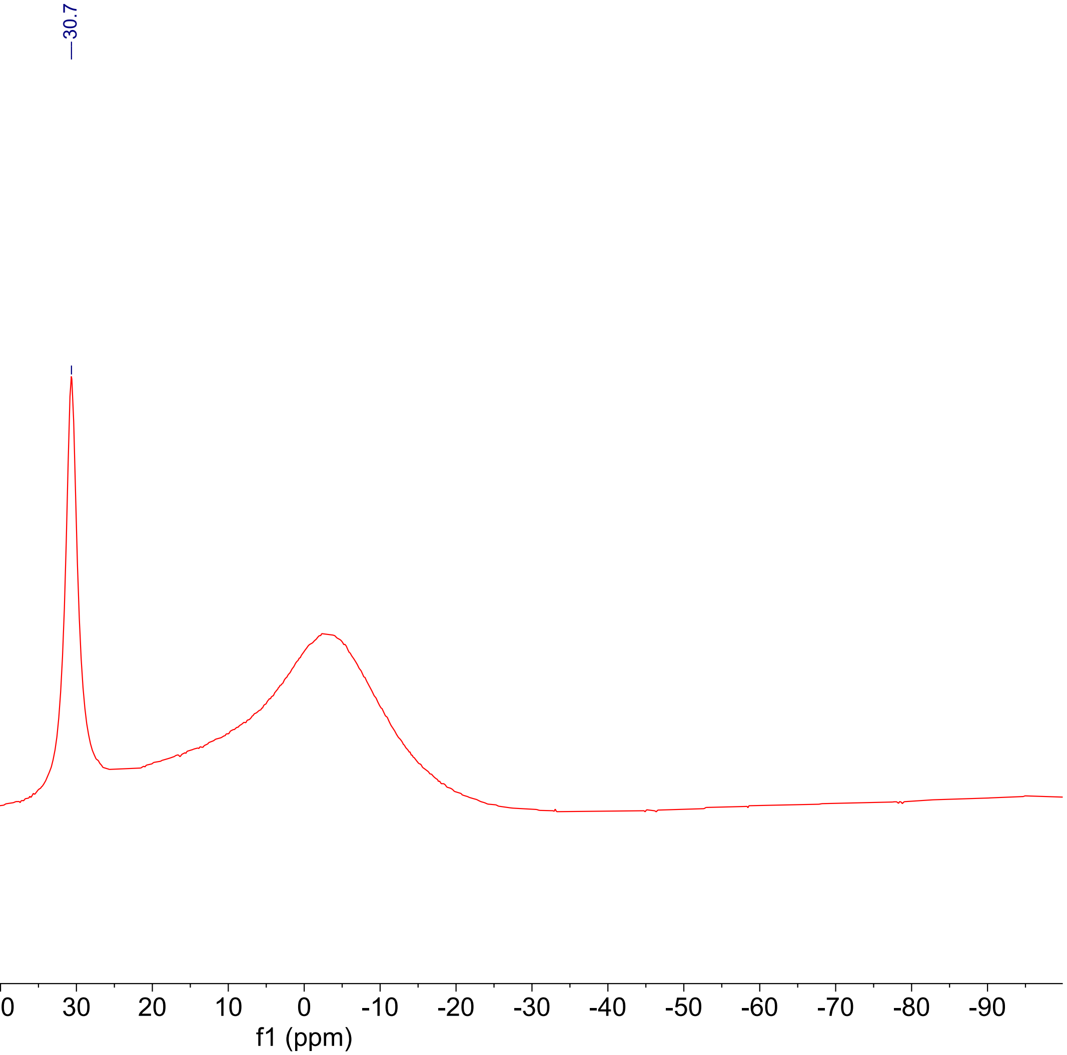

^{1}H NMR of B_2mpg_2 ($CDCl_3$, 500 MHz)

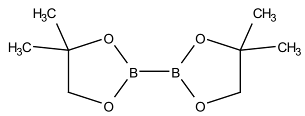

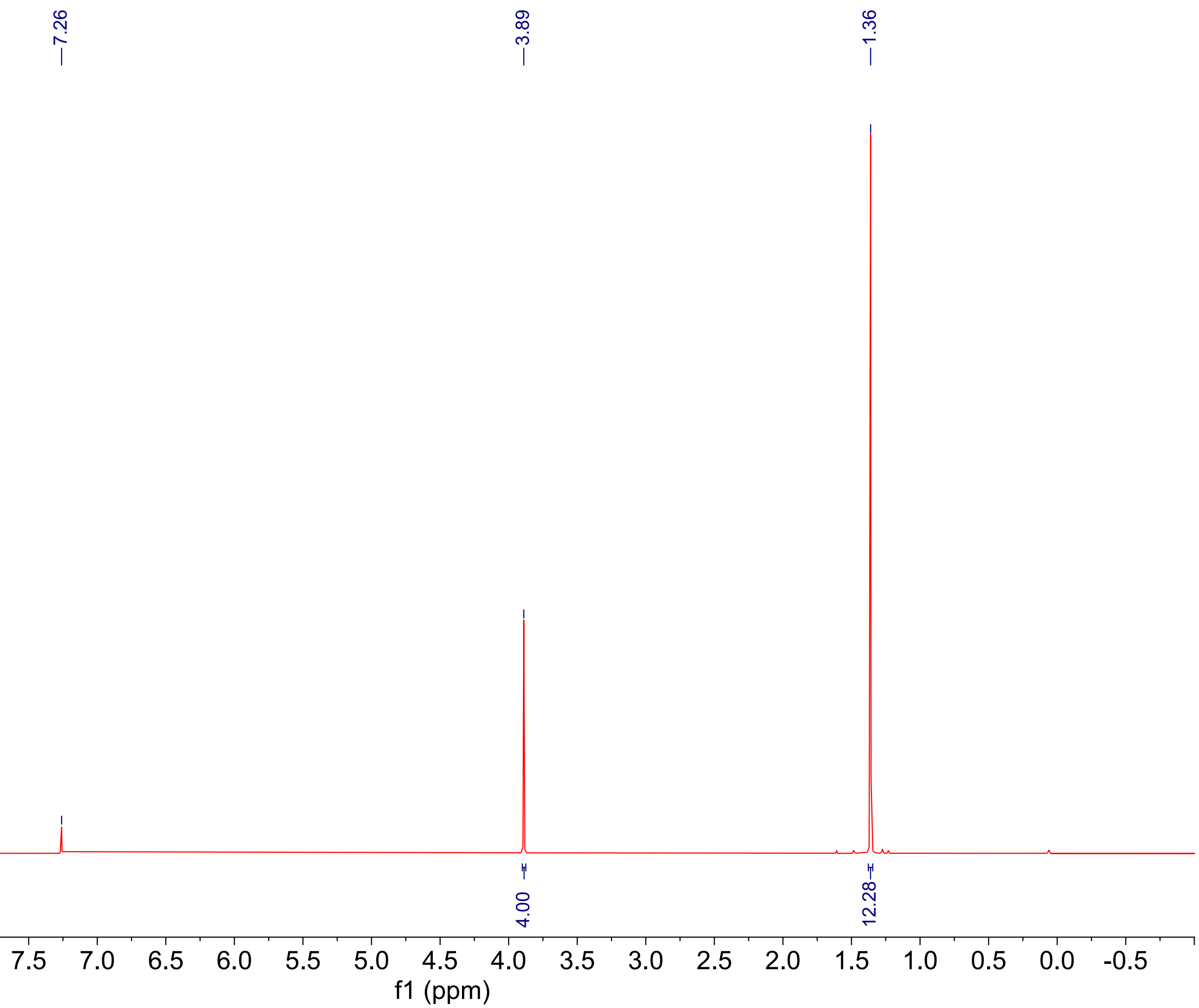

$^{13}C\{^{1}H\}$ NMR of B_2mpg_2 ($CDCl_3$, 126 MHz)

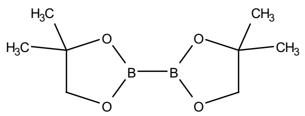

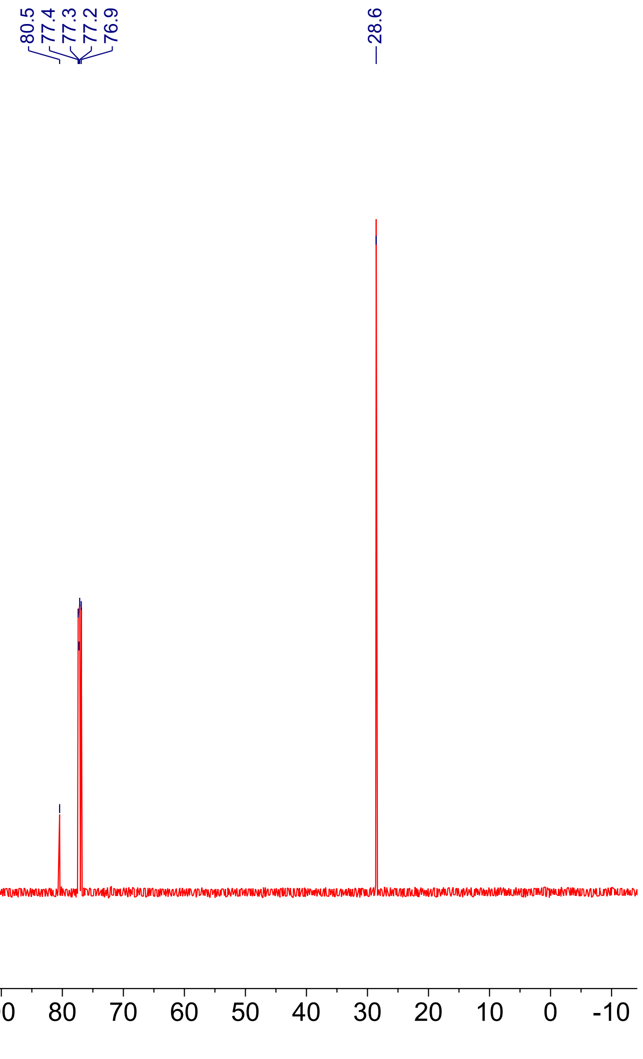

^{11}B NMR of B_2mpg_2 ($CDCl_3$, 160 MHz)

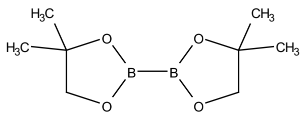

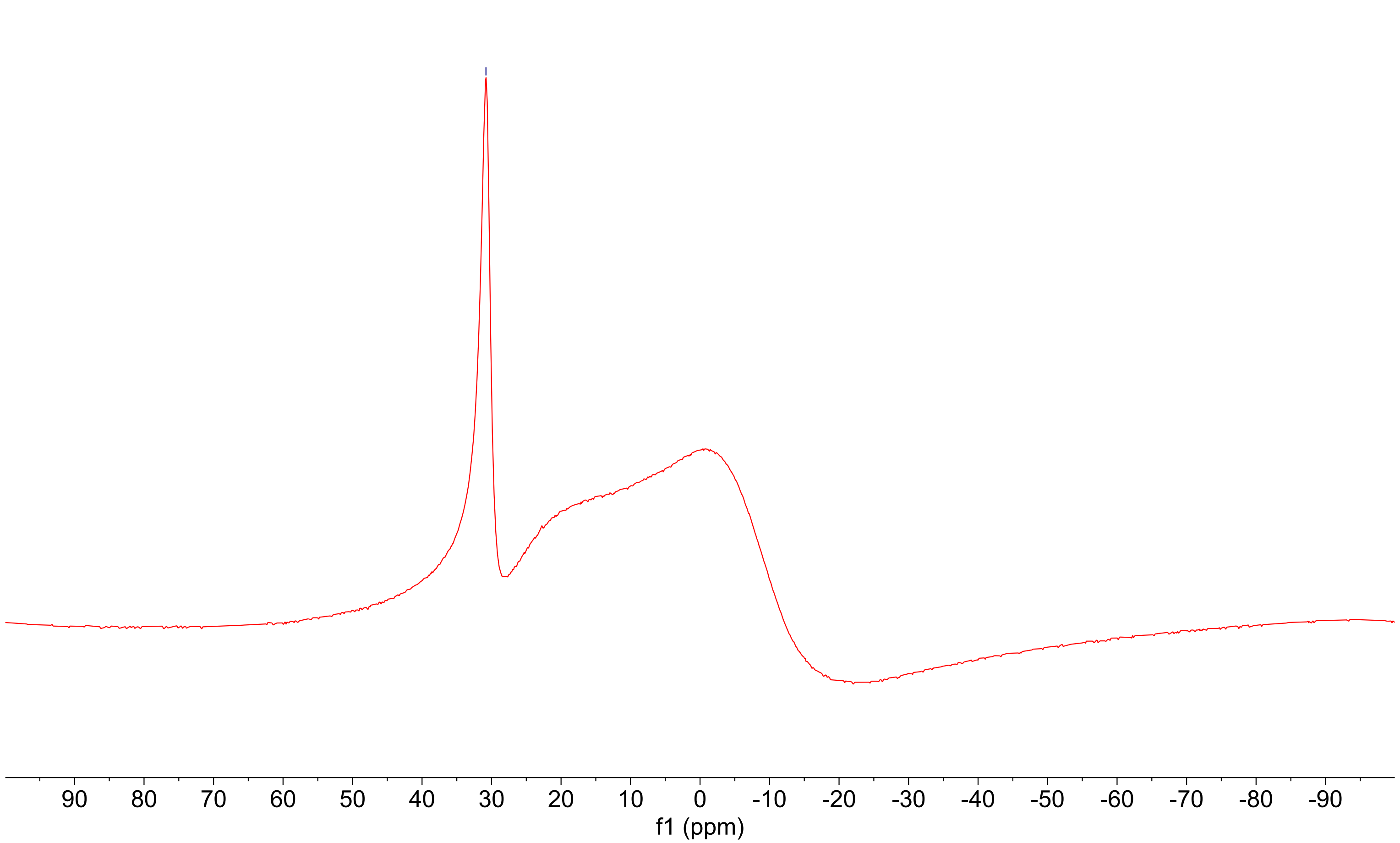

^{1}H NMR of B_2((2*R*,3*R*)bg)$_2$ ($CDCl_3$, 500 MHz)

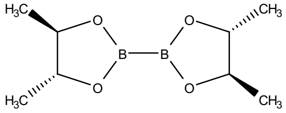

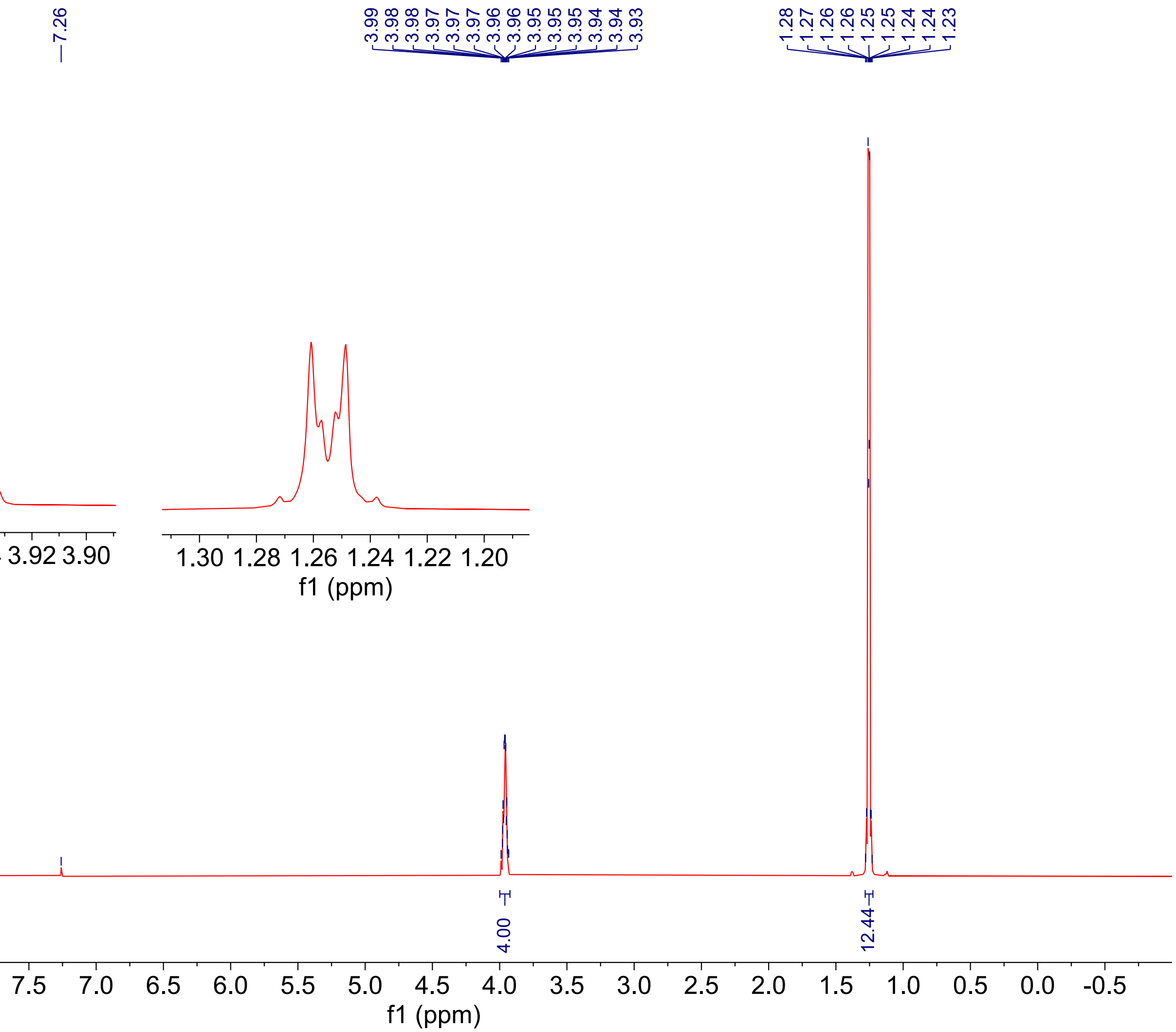

$^{13}C\{^{1}H\}$ NMR of $B_2((2\textit{R},3\textit{R})bg)_2$ ($CDCl_3$, 126 MHz)

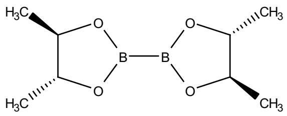

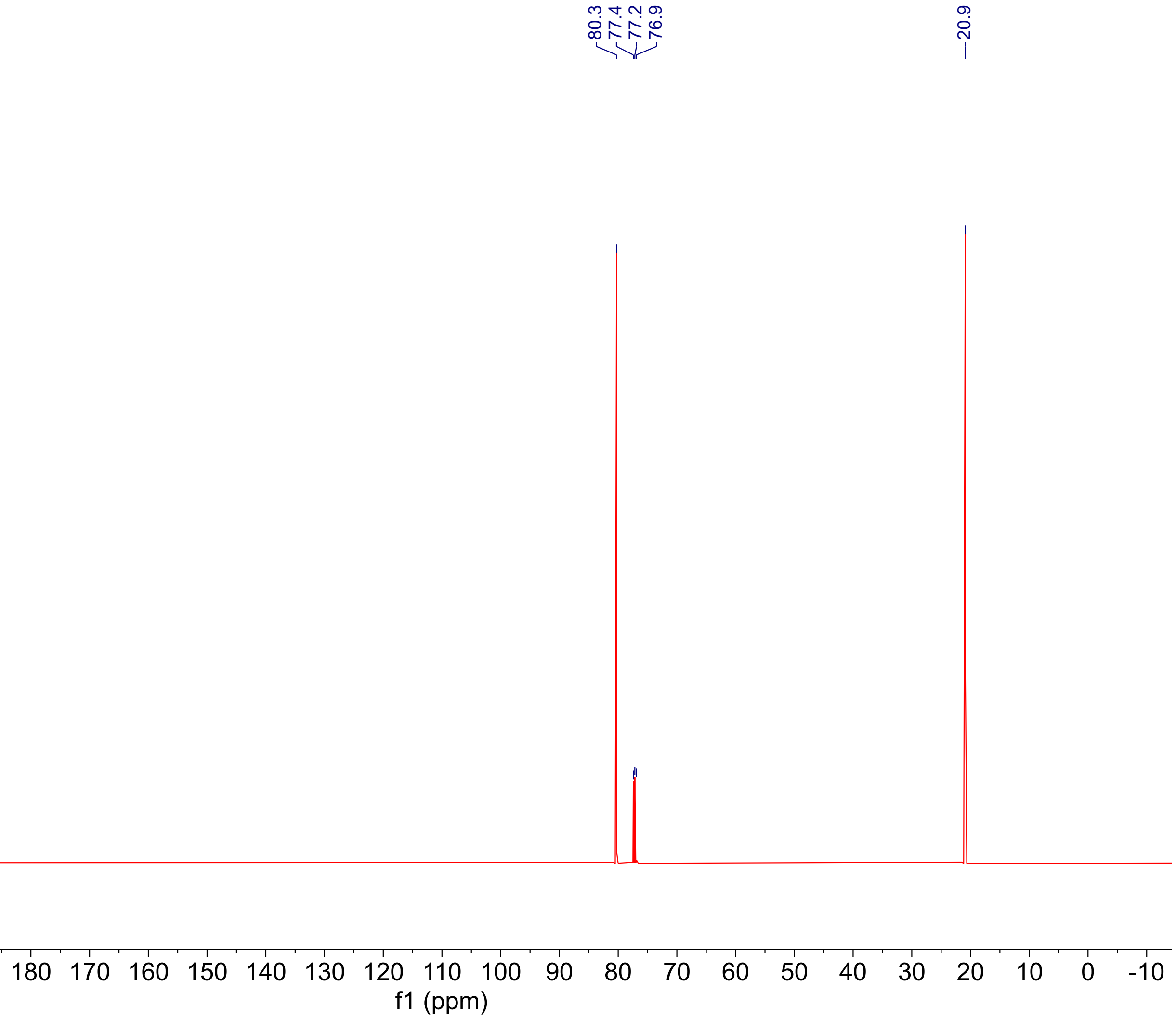

^{11}B NMR of B_2((2*R*,3*R*)bg)$_2$ ($CDCl_3$, 160 MHz)

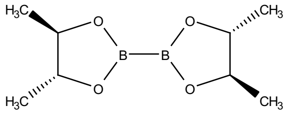

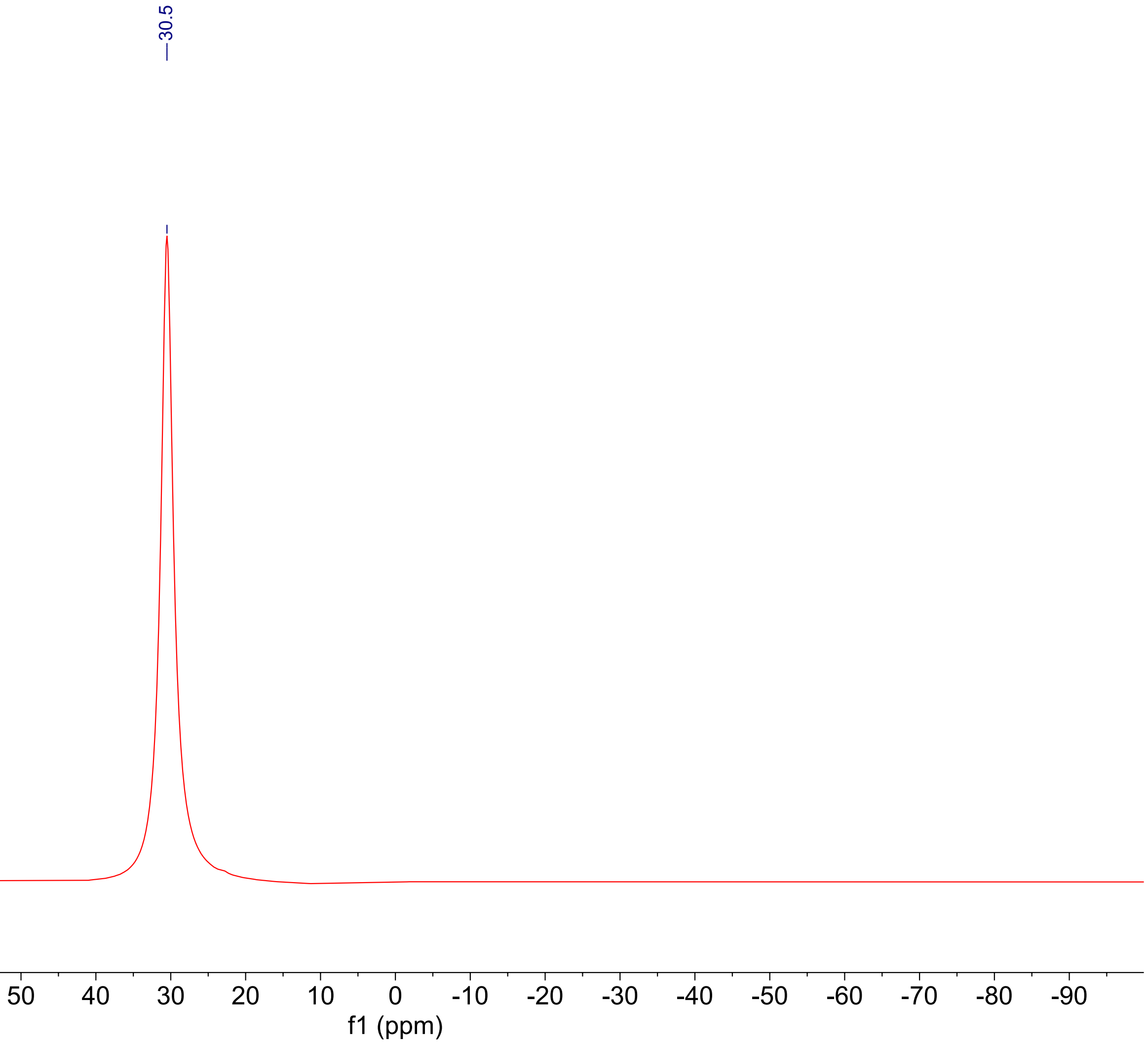

^{1}H NMR of *ortho* Bpg-borylated aniline (2.1) ($CDCl_3$, 500 MHz)

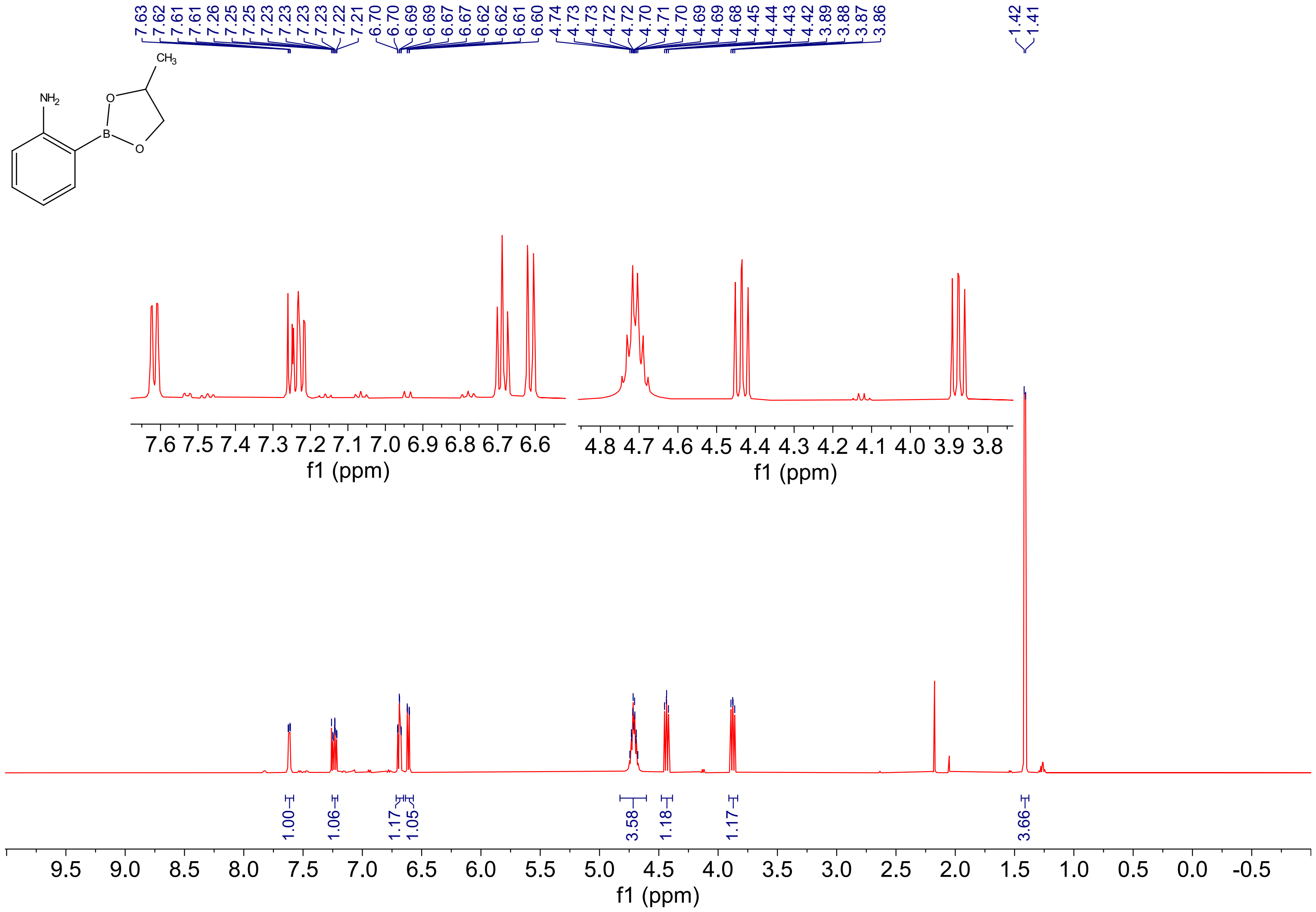

$^{13}C\{^1H\}$ NMR of *ortho* Bpg-borylated aniline (2.1) ($CDCl_3$, 126 MHz)

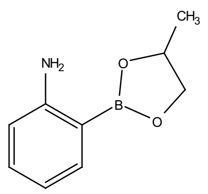

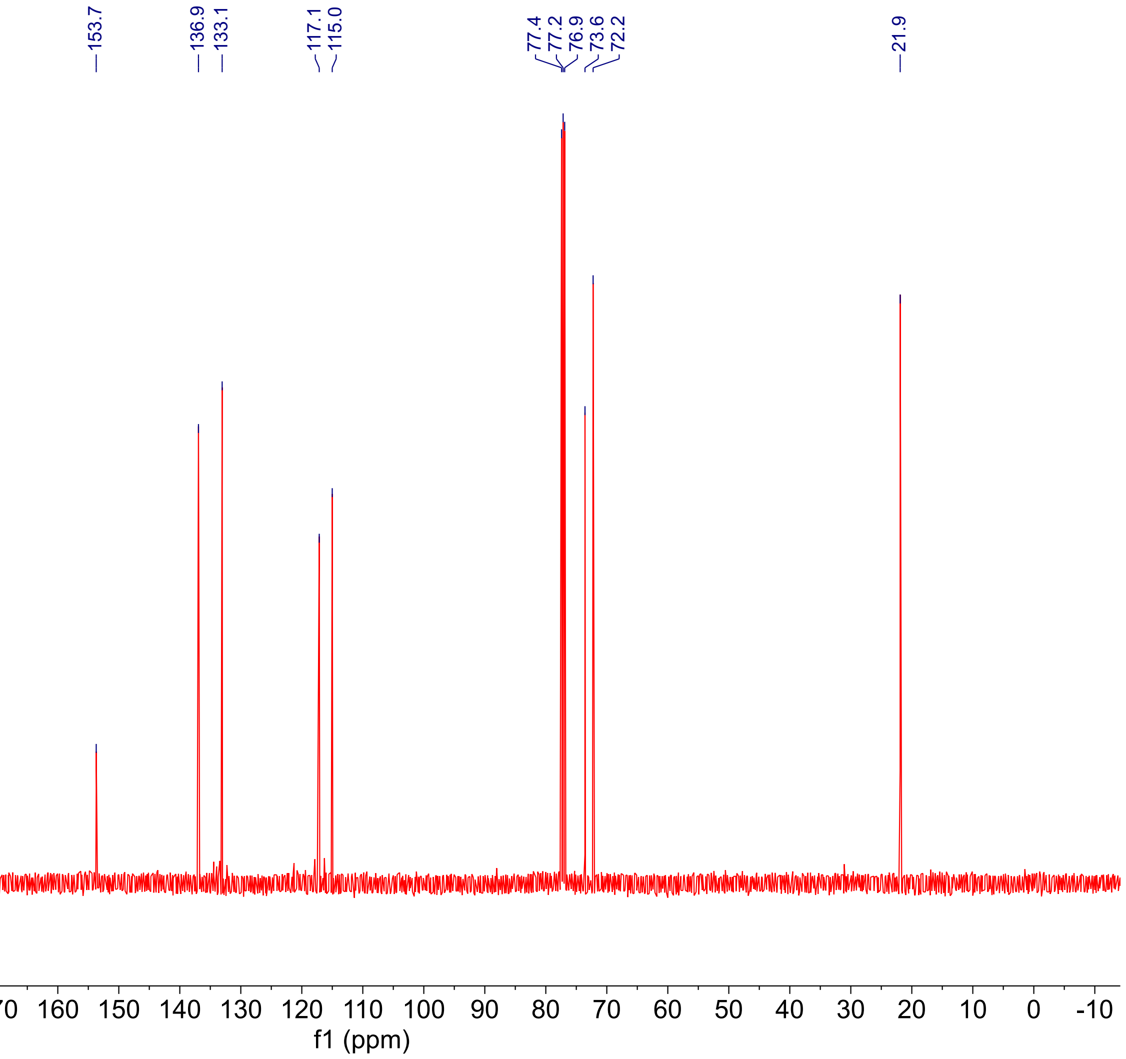

^{11}B NMR of *ortho* Bpg-borylated aniline (2.1) ($CDCl_3$, 160 MHz)

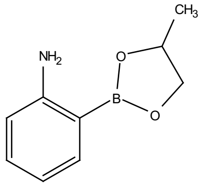

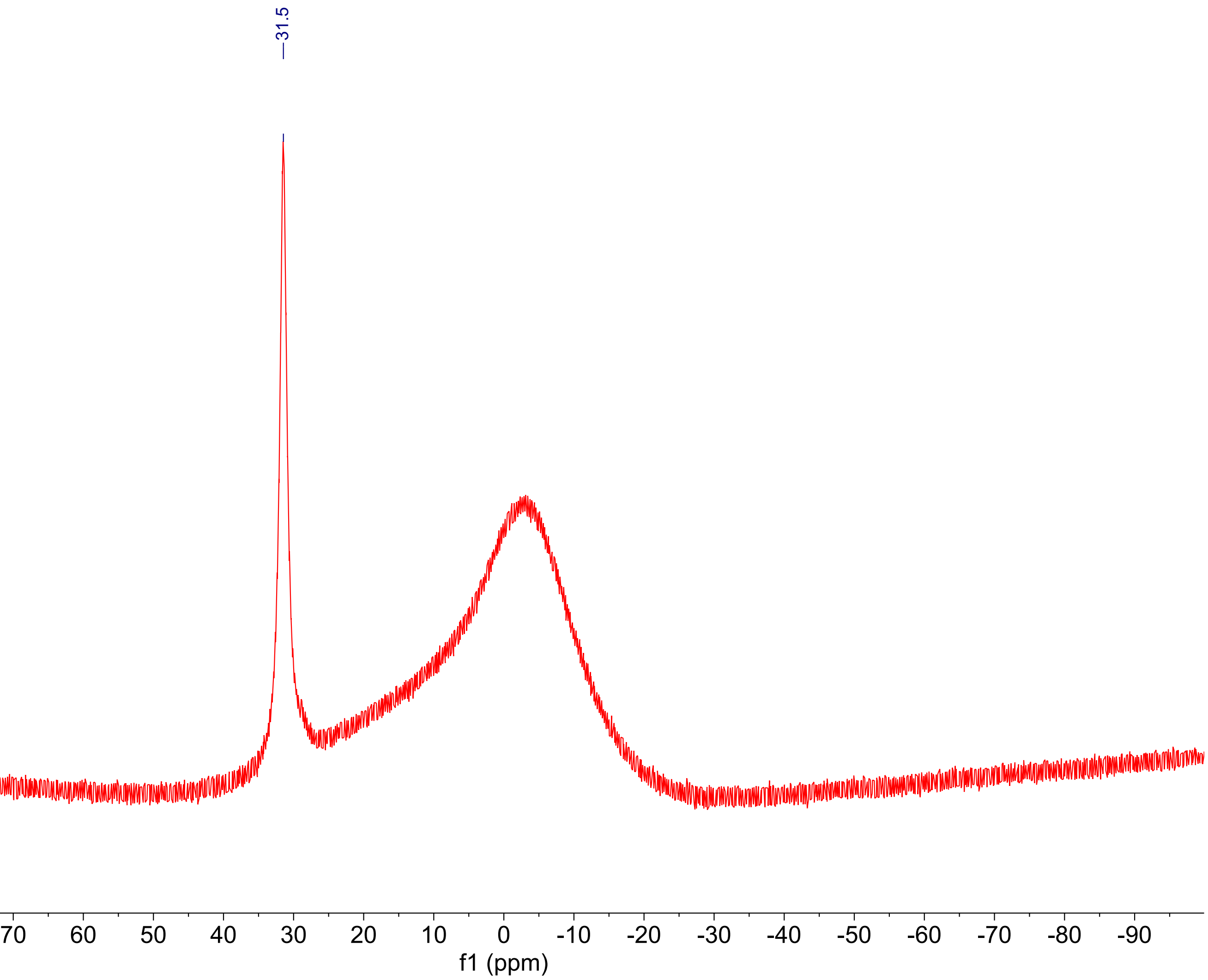

^{1}H NMR of *ortho* Bpg-borylated phenylacetamide (2.3) ($CDCl_3$, 500 MHz)

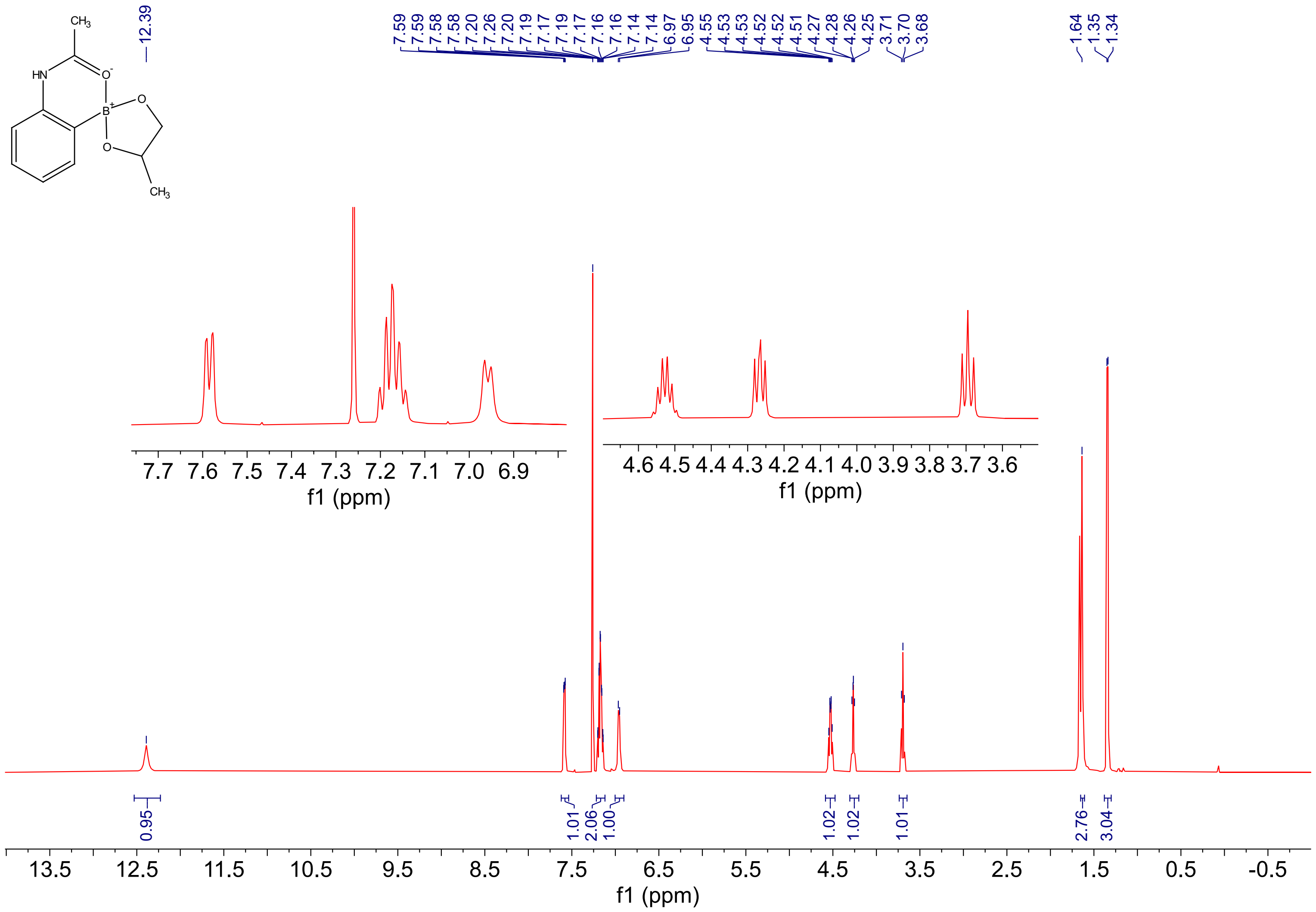

$^{13}C\{^{1}H\}$ NMR of *ortho* Bpg-borylated phenylacetamide (2.3) ($CDCl_3$, 160 MHz)

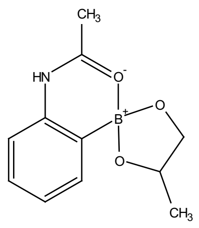

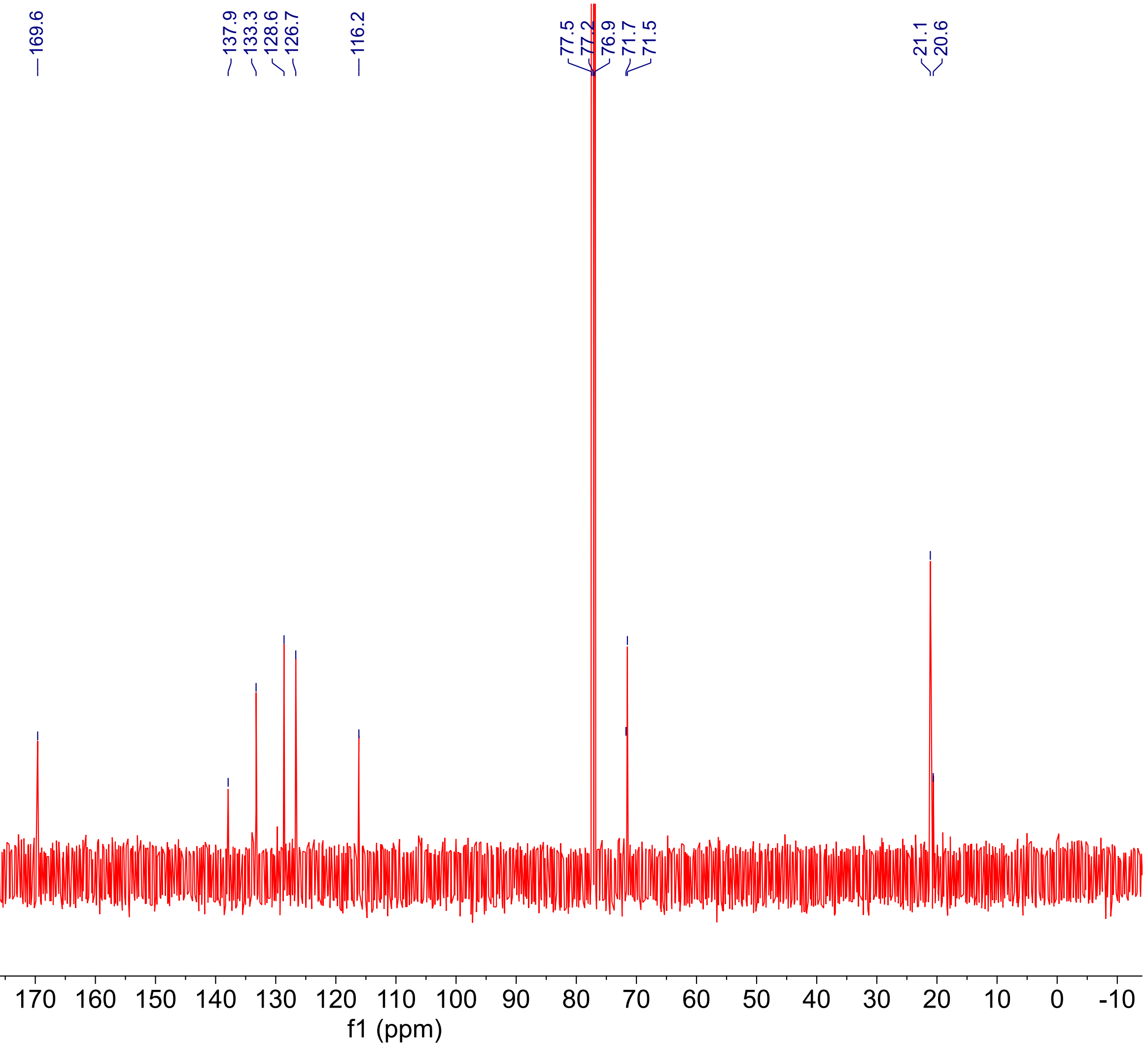

^{11}B NMR of *ortho* Bpg-borylated phenylacetamide (2.3) ($CDCl_3$, 126 MHz)

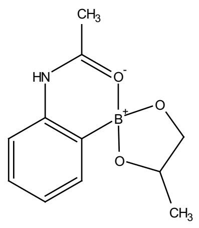

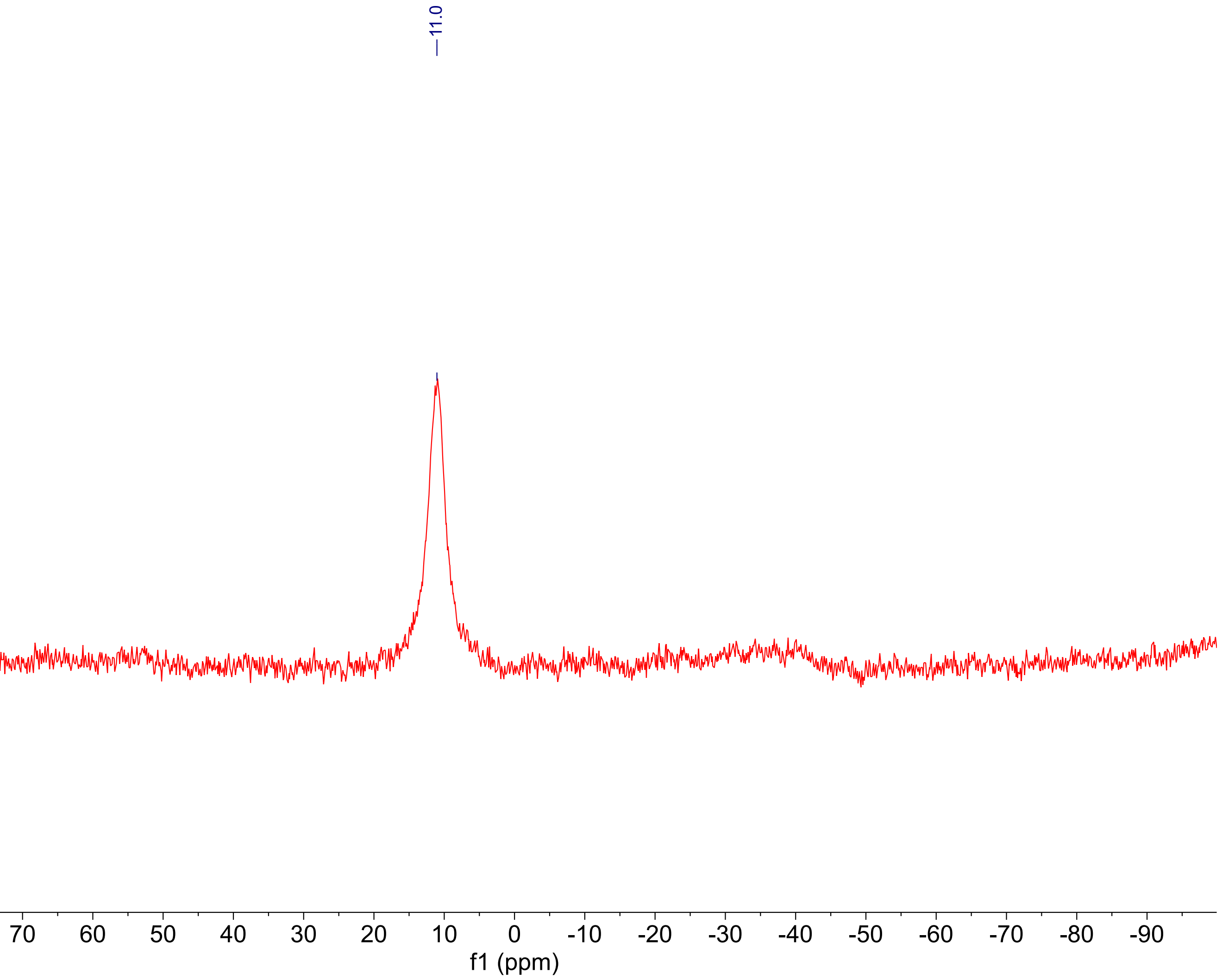

^{1}H NMR spectrum of the reaction mixture of CHB aniline (3) ($CDCl_3$, 500 MHz)

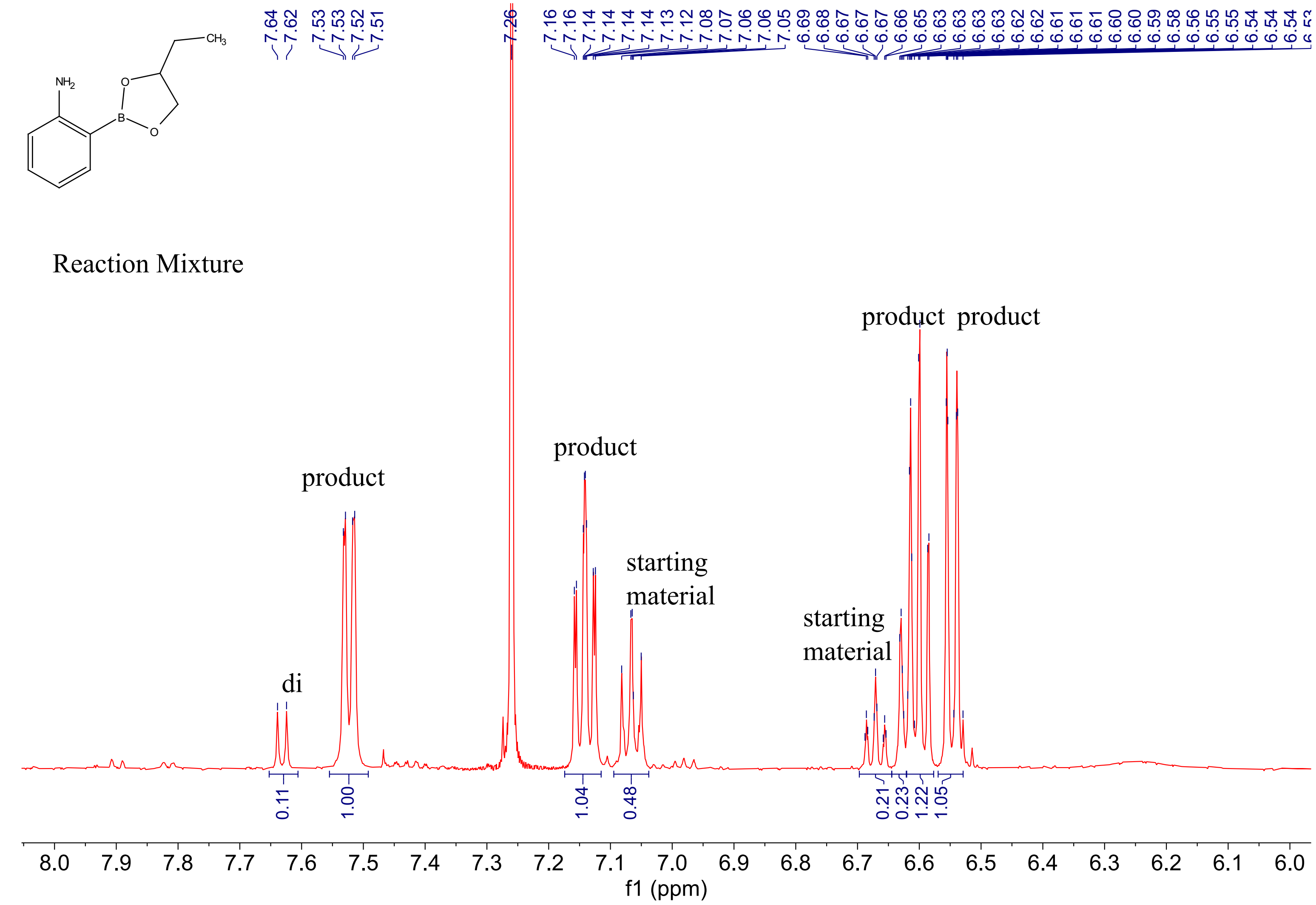

^{1}H NMR of *ortho* Bbg-borylated aniline (3) ($CDCl_3$, 500 MHz)

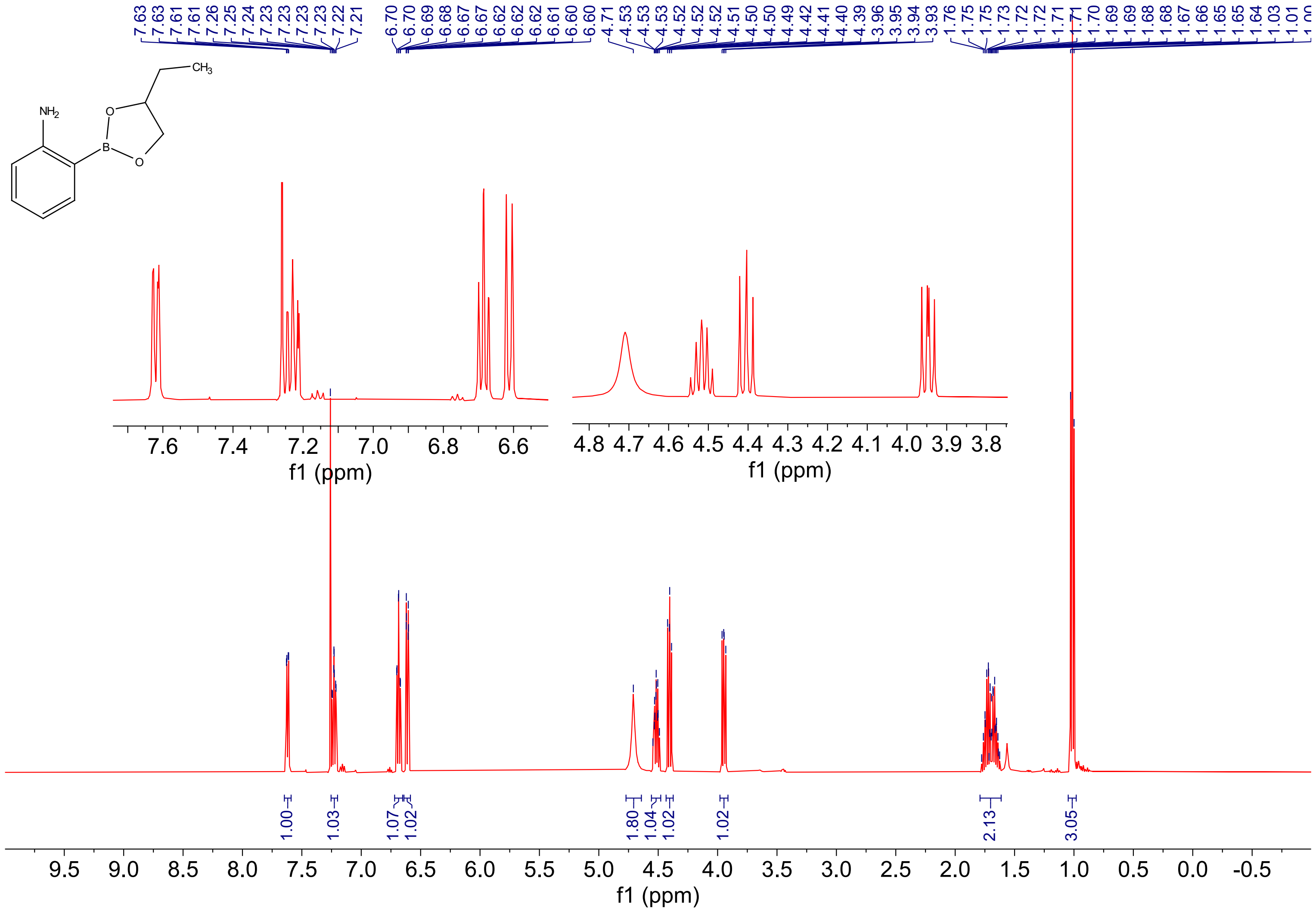

$^{13}C\{^{1}H\}$ NMR of *ortho* Bbg-borylated aniline (3) ($CDCl_3$, 160 MHz)

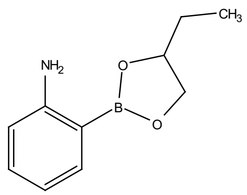

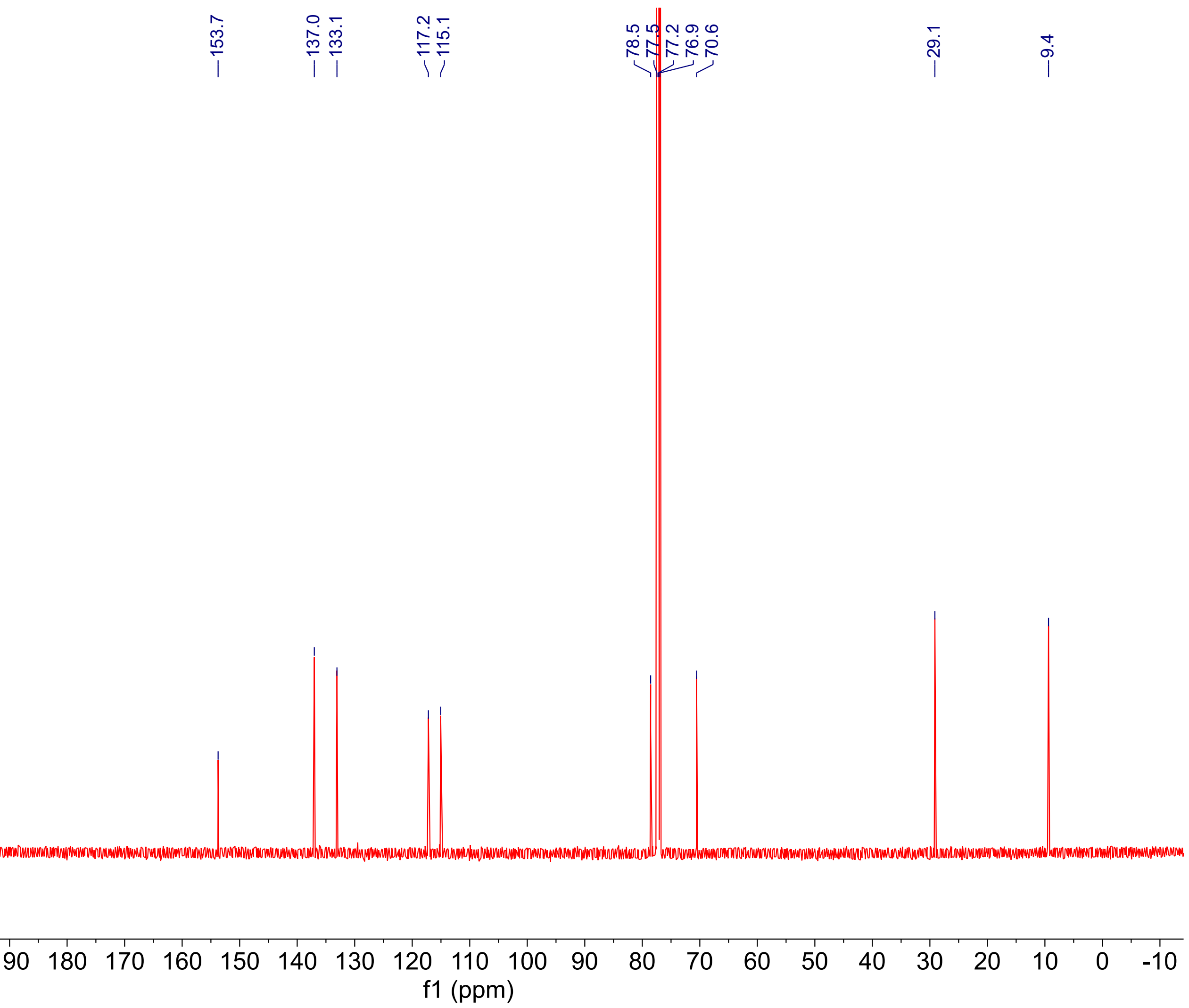

^{11}B NMR of *ortho* Bbg-borylated aniline (3) ($CDCl_3$, 126 MHz)

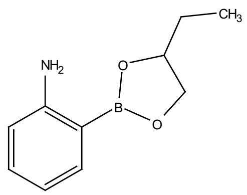

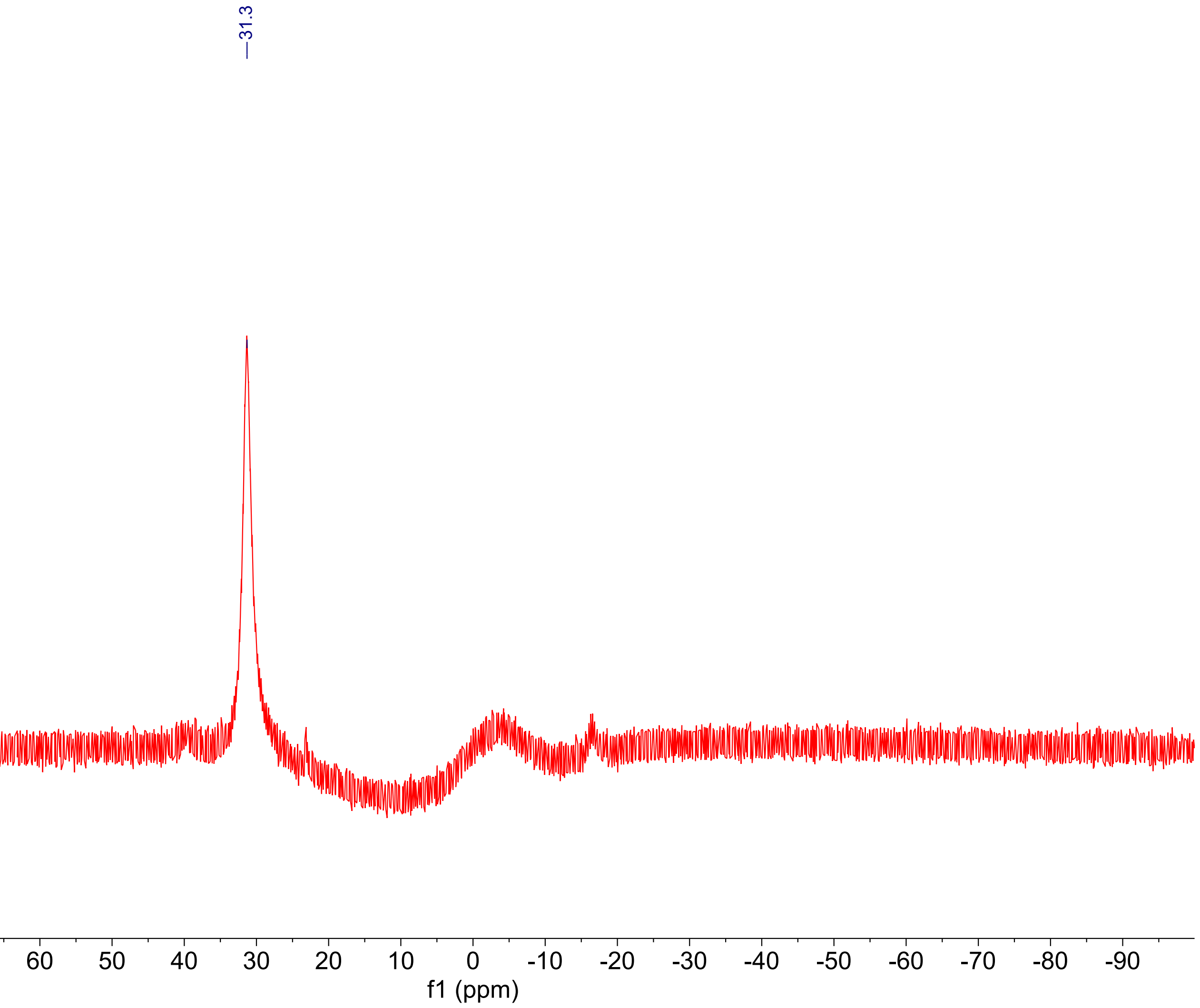

^{1}H NMR spectrum of the reaction mixture of CHB 4-chloroaniline (4) ($CDCl_3$, 500 MHz)

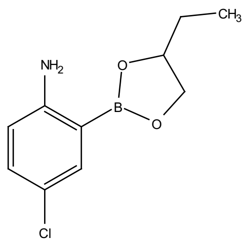

Reaction Mixture

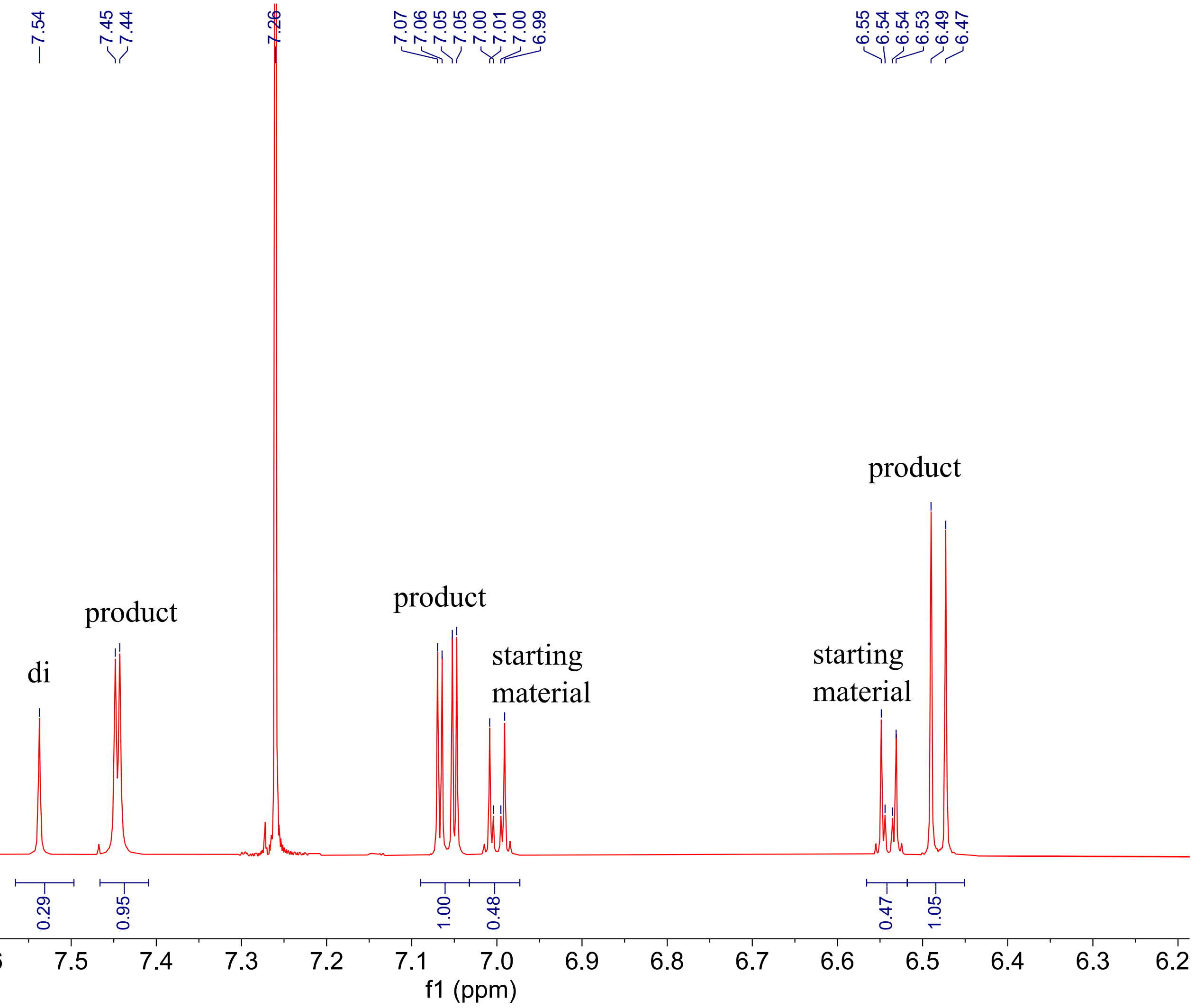

^{1}H NMR of *ortho* Bbg-borylated 4-chloroaniline (4) ($CDCl_3$, 500 MHz)

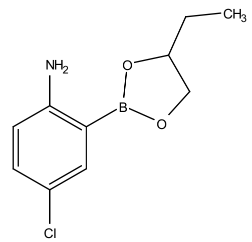

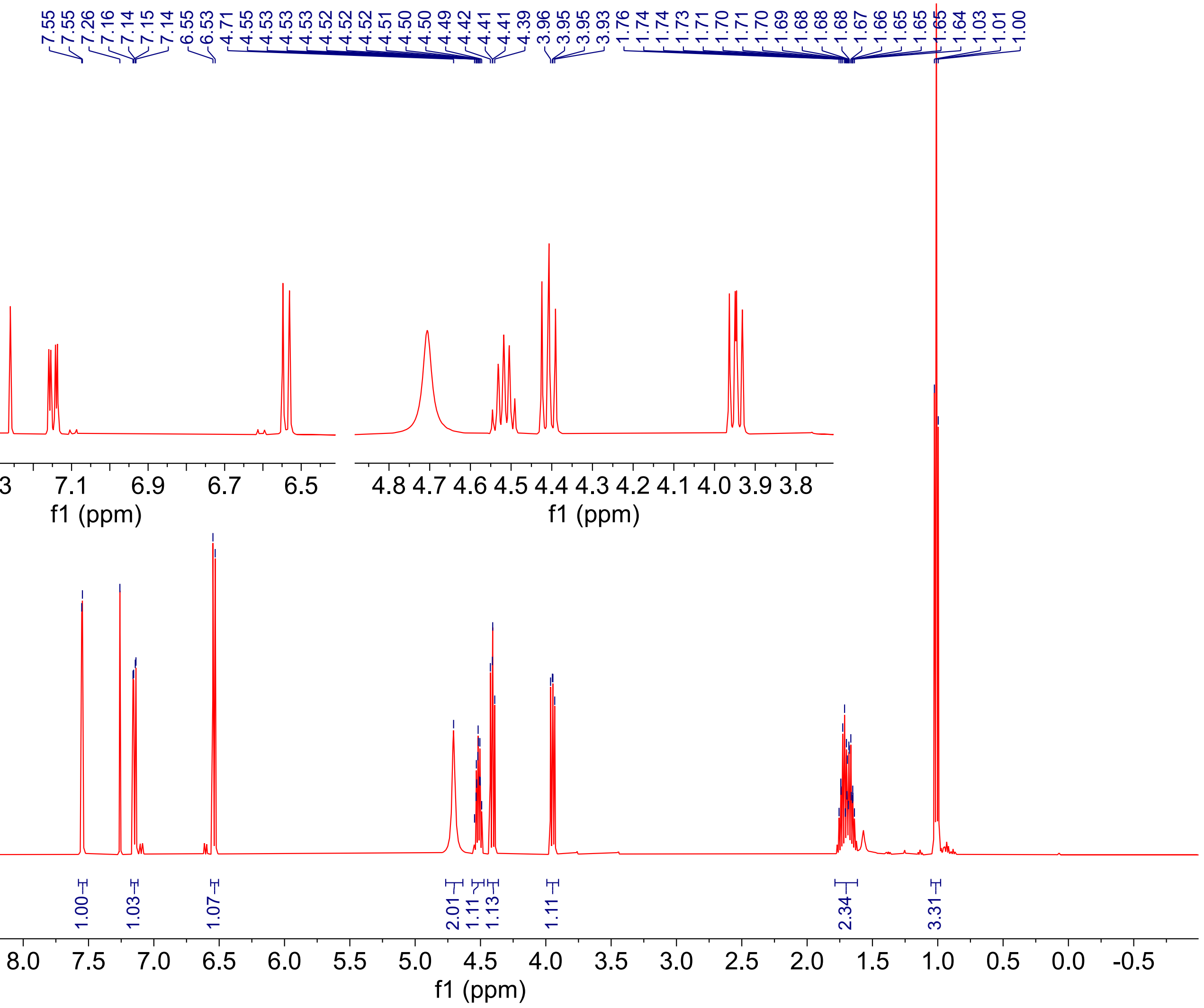

$^{13}C\{^{1}H\}$ NMR of *ortho* Bbg-borylated 4-chloroaniline (4) ($CDCl_3$, 160 MHz)

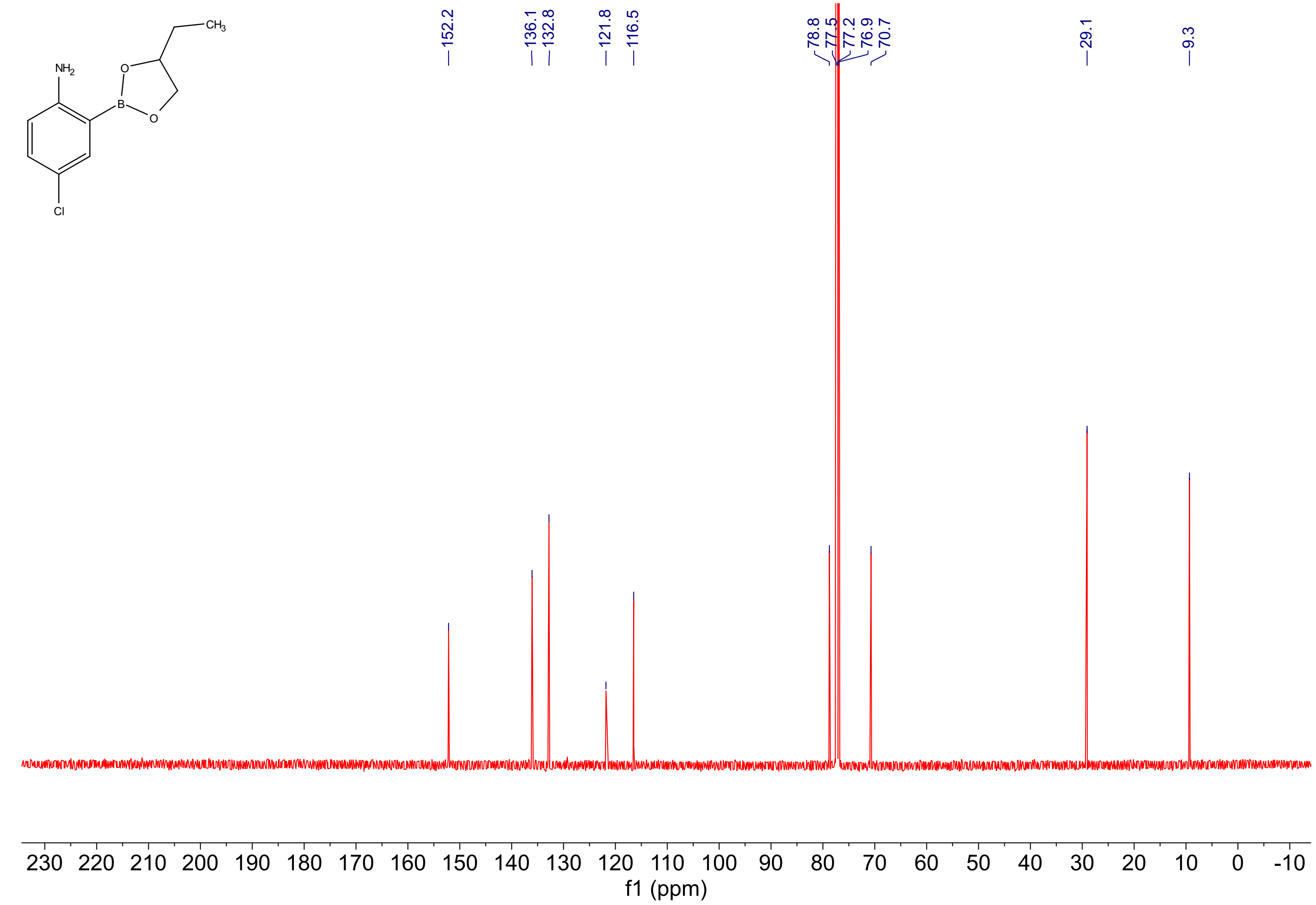

^{11}B NMR of *ortho* Bbg-borylated 4-chloroaniline (4) ($CDCl_3$, 126 MHz)

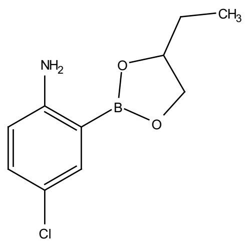

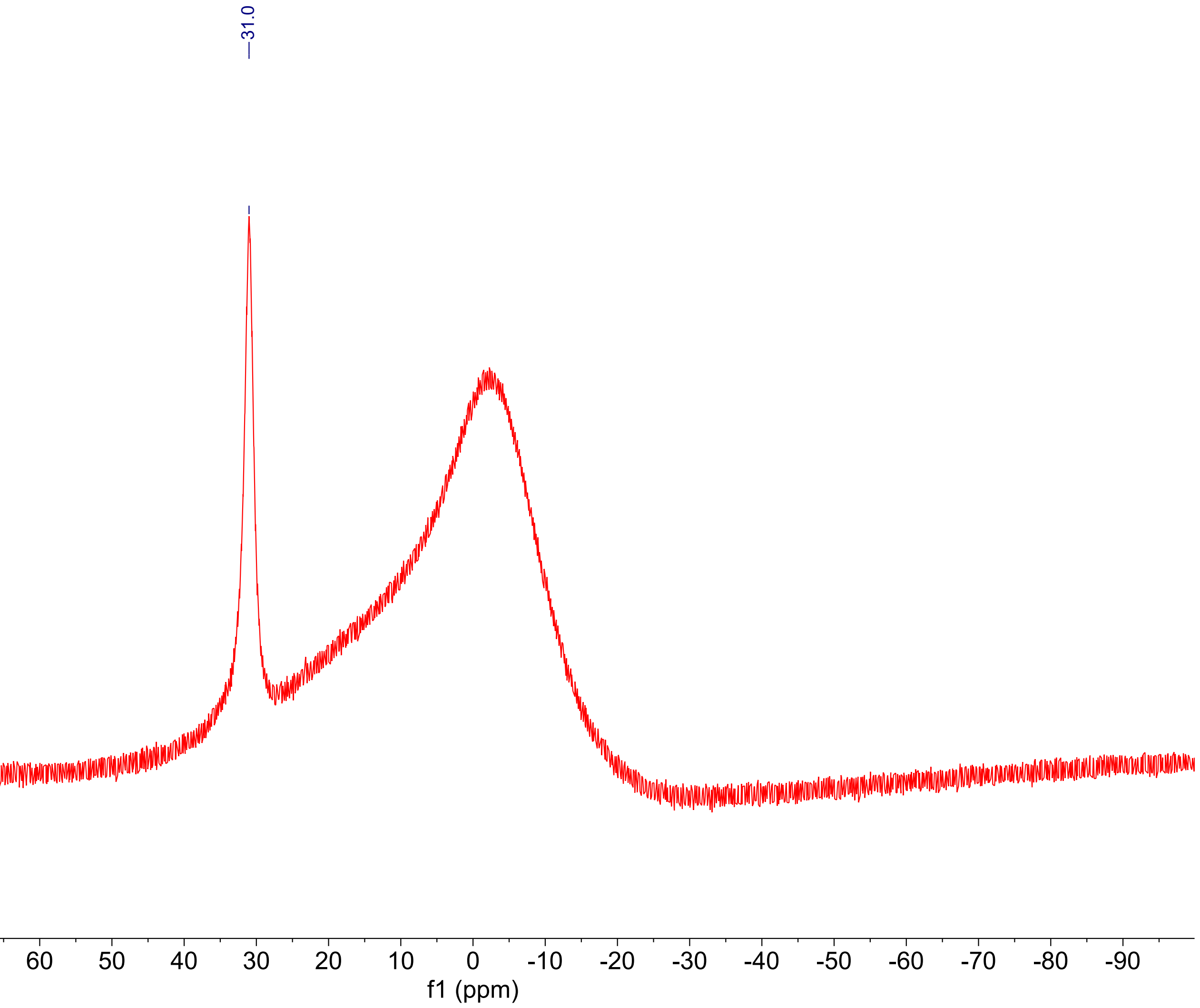

^{1}H NMR spectrum of the reaction mixture of CHB 4-bromoaniline (5) ($CDCl_3$, 500 MHz)

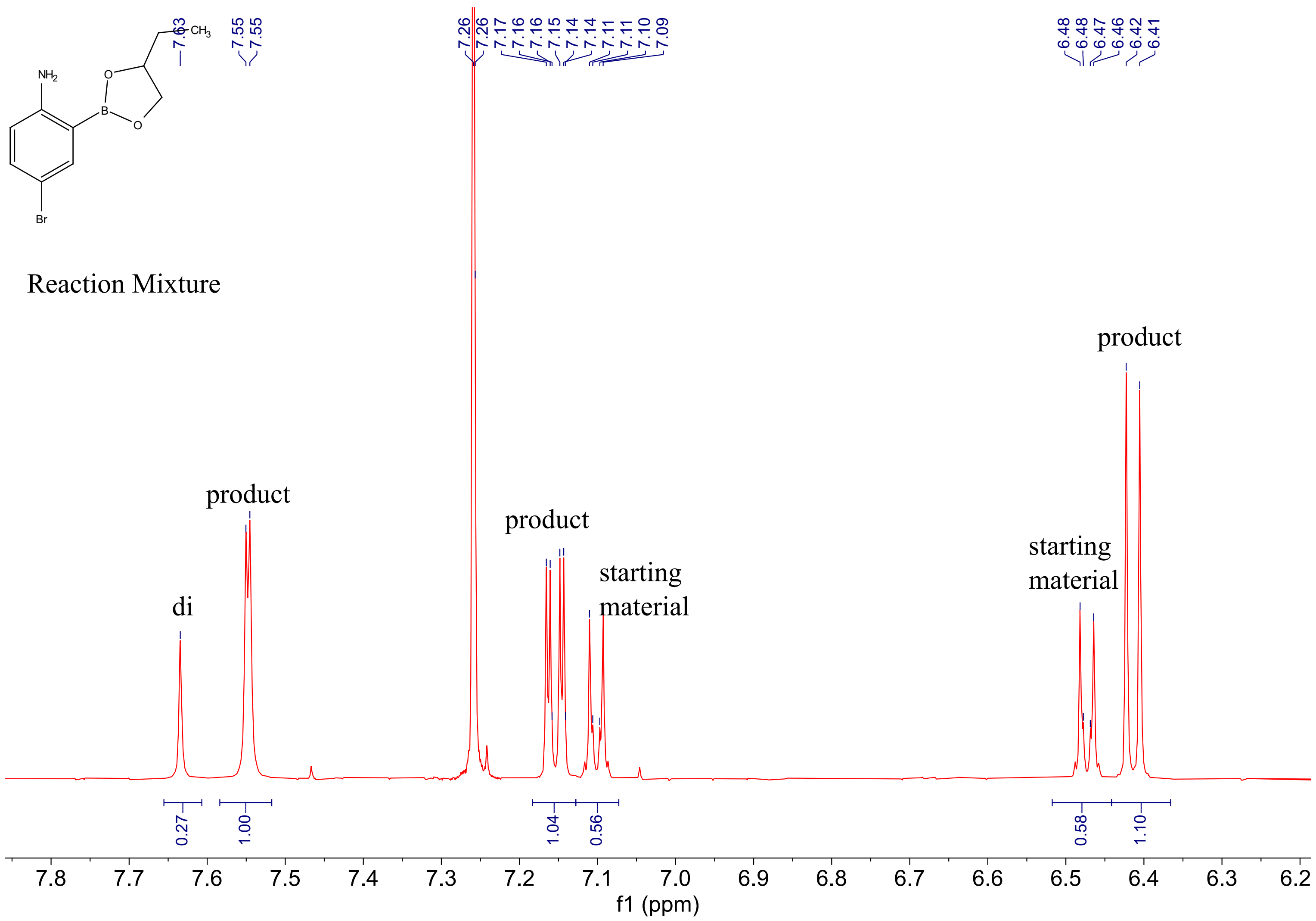

^{1}H NMR of *ortho* Bbg-borylated 4-bromoaniline (5) ($CDCl_3$, 500 MHz)

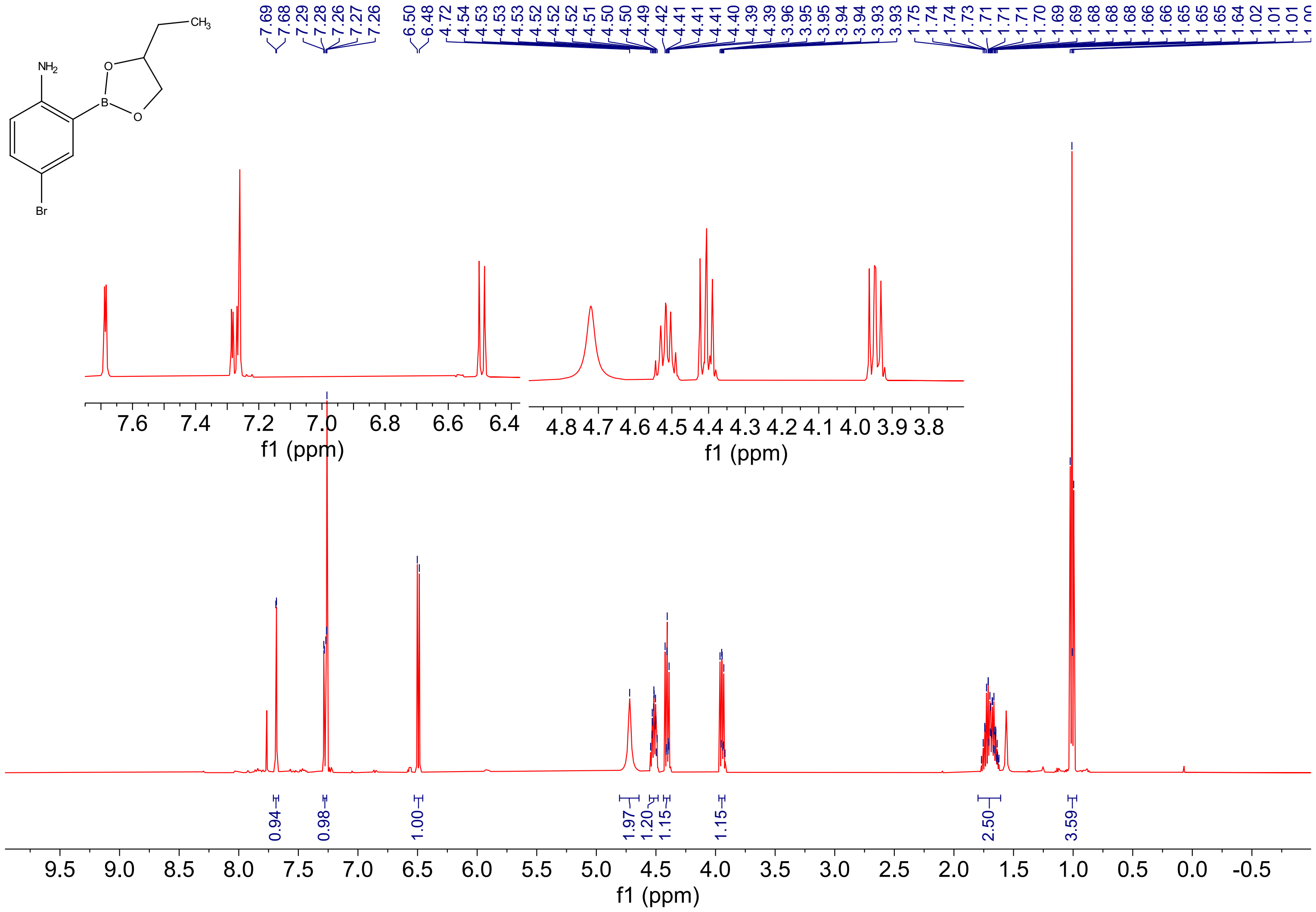

$^{13}C\{^1H\}$ **NMR of *ortho* Bbg-borylated 4-bromoaniline (5) ($CDCl_3$, 160 MHz)**

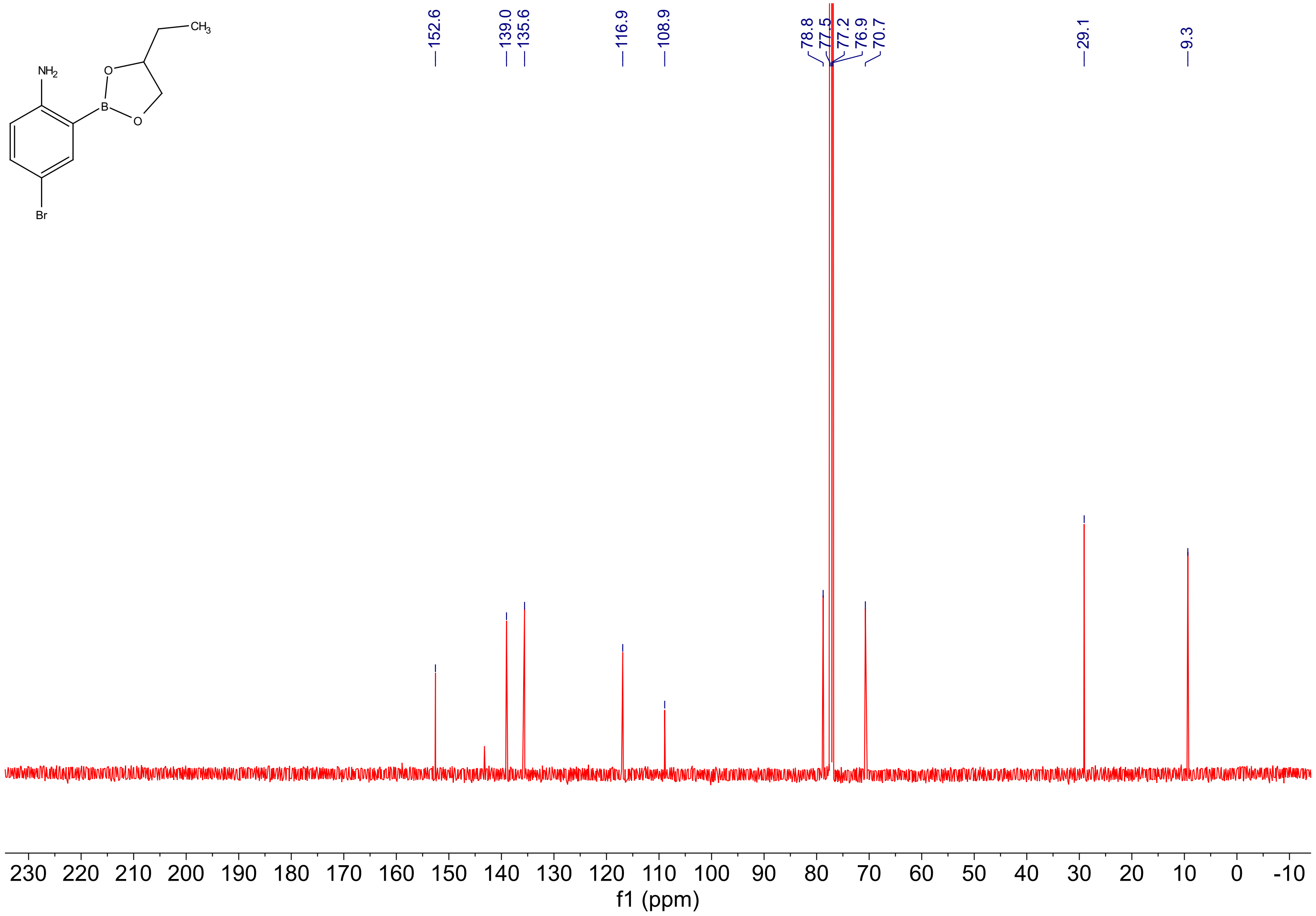

^{11}B NMR of *ortho* Bbg-borylated 4-bromoaniline (5) ($CDCl_3$, 126 MHz)

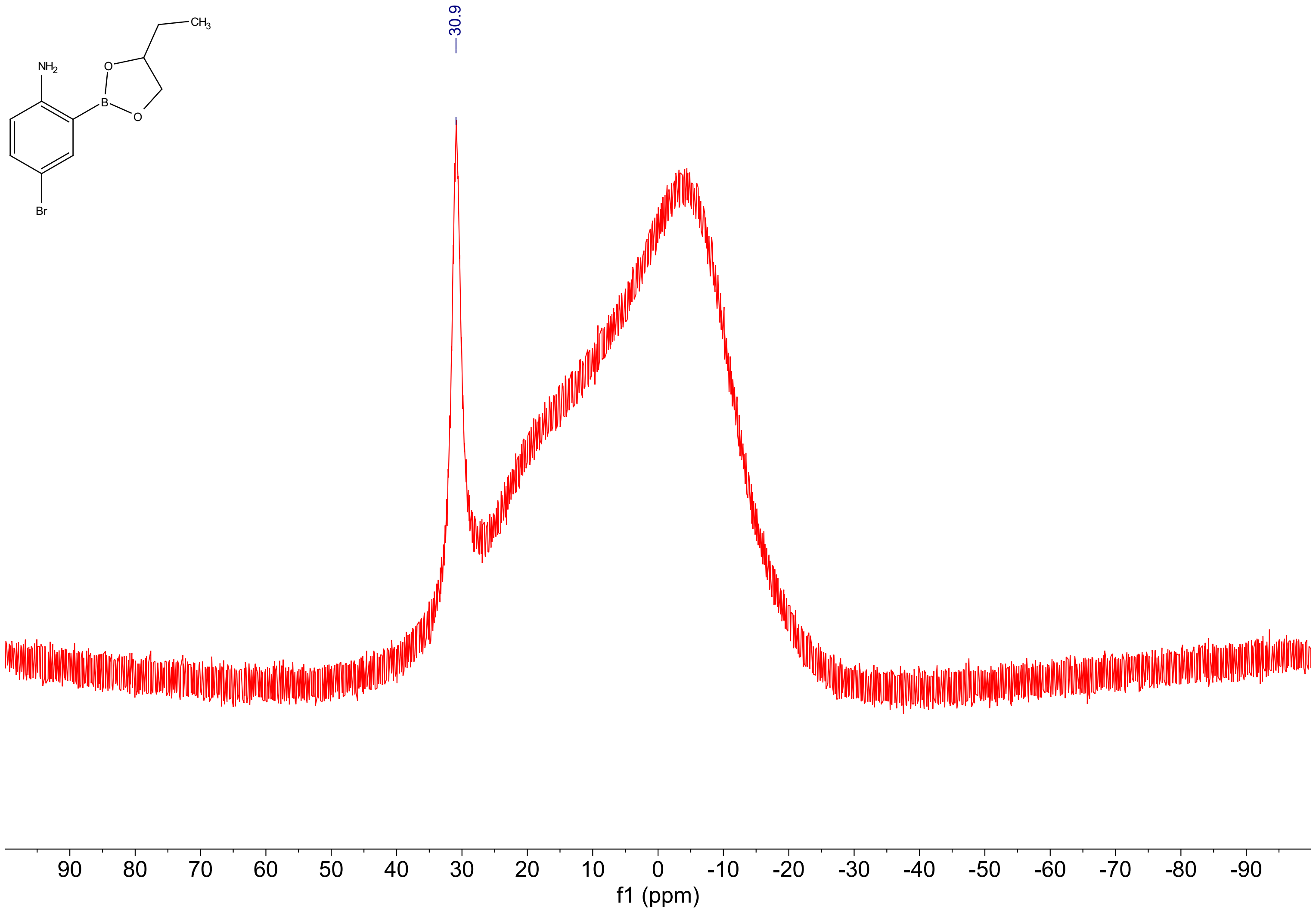

^{1}H NMR spectrum of the reaction mixture of CHB 4-iodoaniline (6) ($CDCl_3$, 500 MHz)

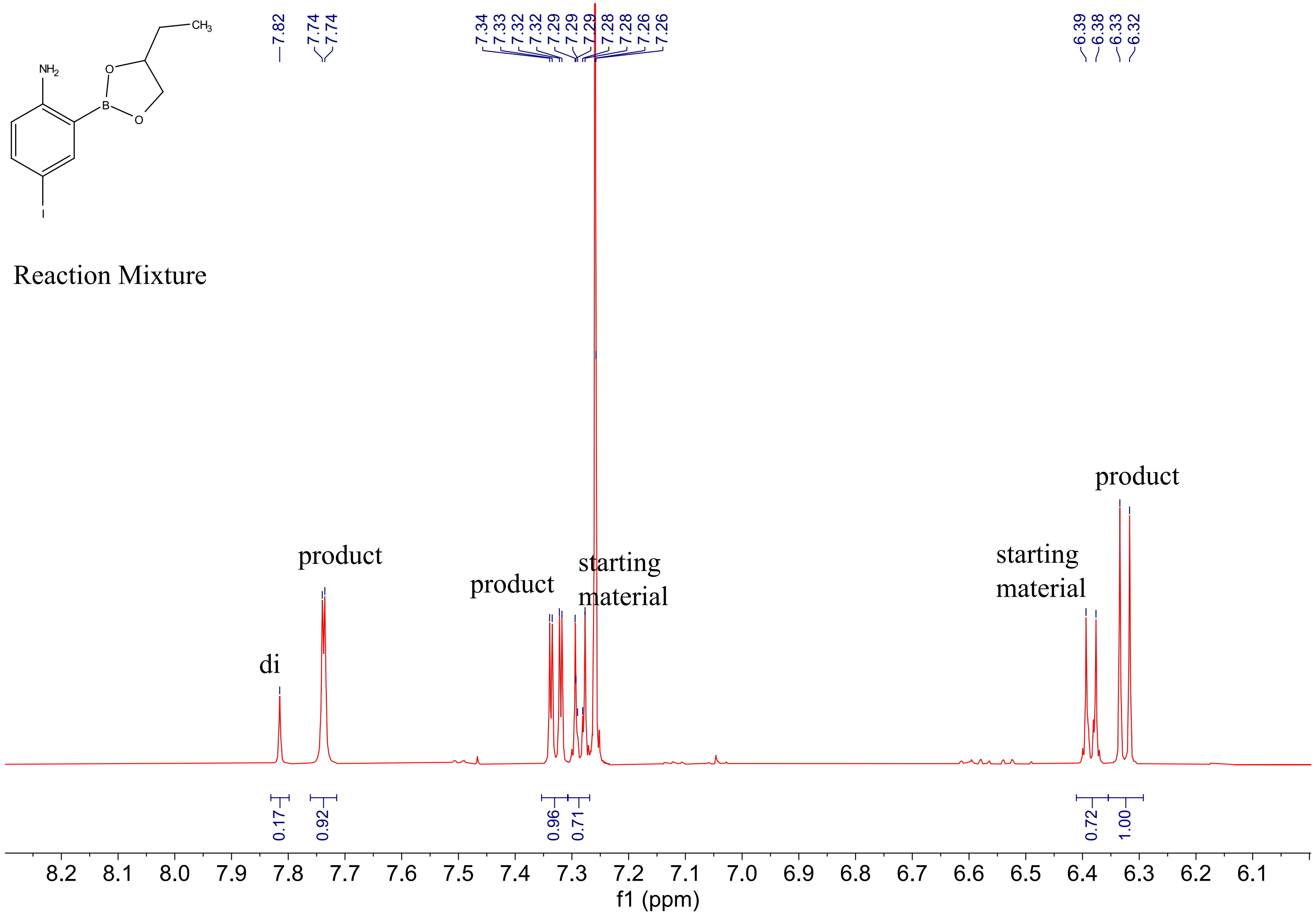

^{1}H NMR of *ortho* Bbg-borylated 4-iodoaniline (6) ($CDCl_3$, 500 MHz)

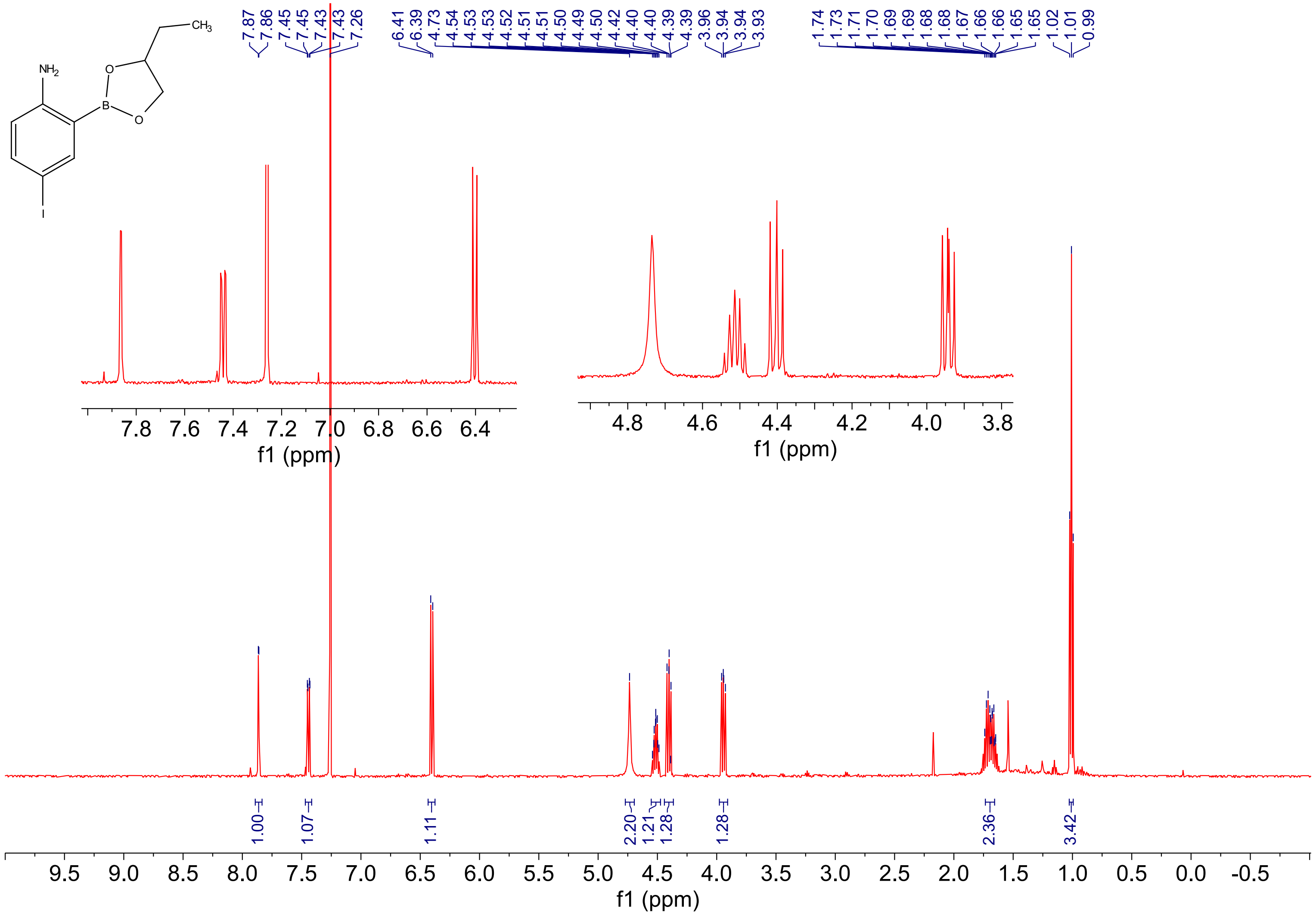

$^{13}C\{^1H\}$ NMR of *ortho* Bbg-borylated 4-iodoaniline (6) ($CDCl_3$, 160 MHz)

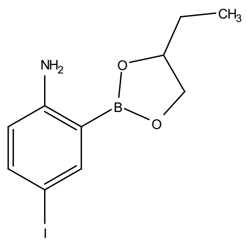

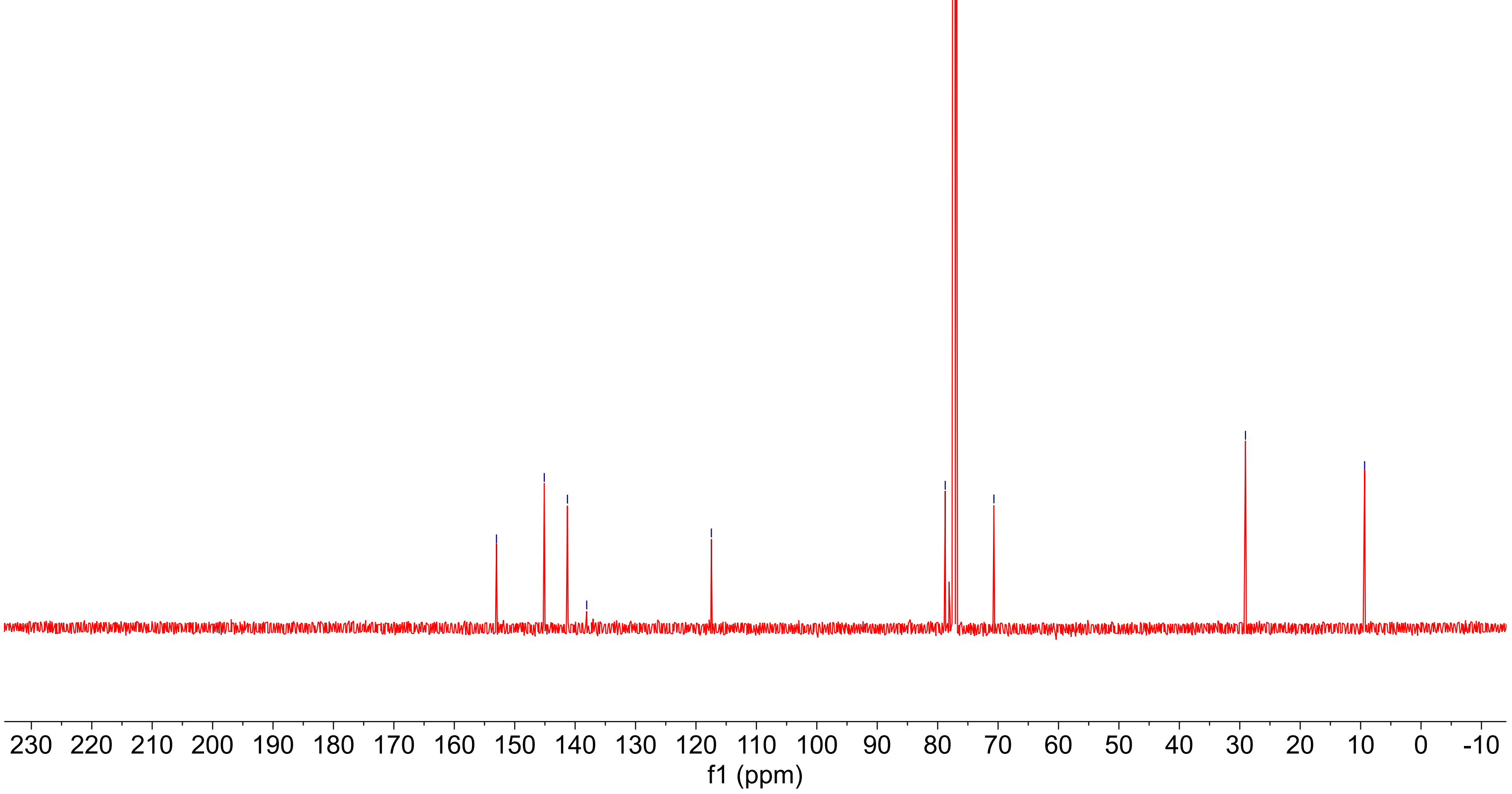

^{11}B NMR of *ortho* Bbg-borylated 4-iodoaniline (6) ($CDCl_3$, 126 MHz)

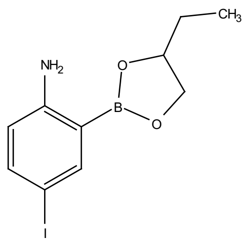

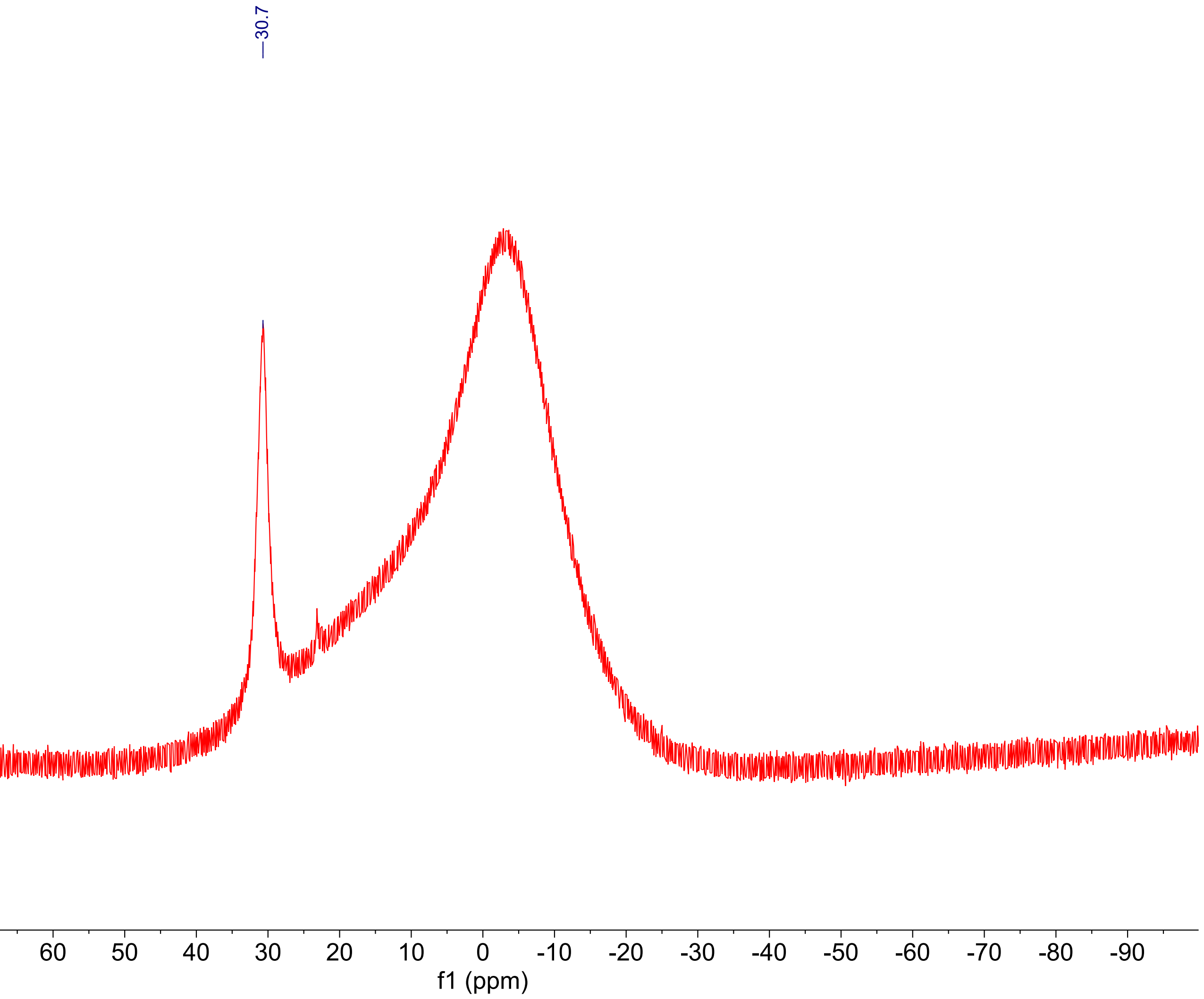

^{1}H NMR spectrum of the reaction mixture of CHB 3-chloroaniline (7) ($CDCl_3$, 500 MHz)

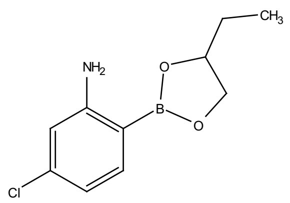

Reaction Mixture

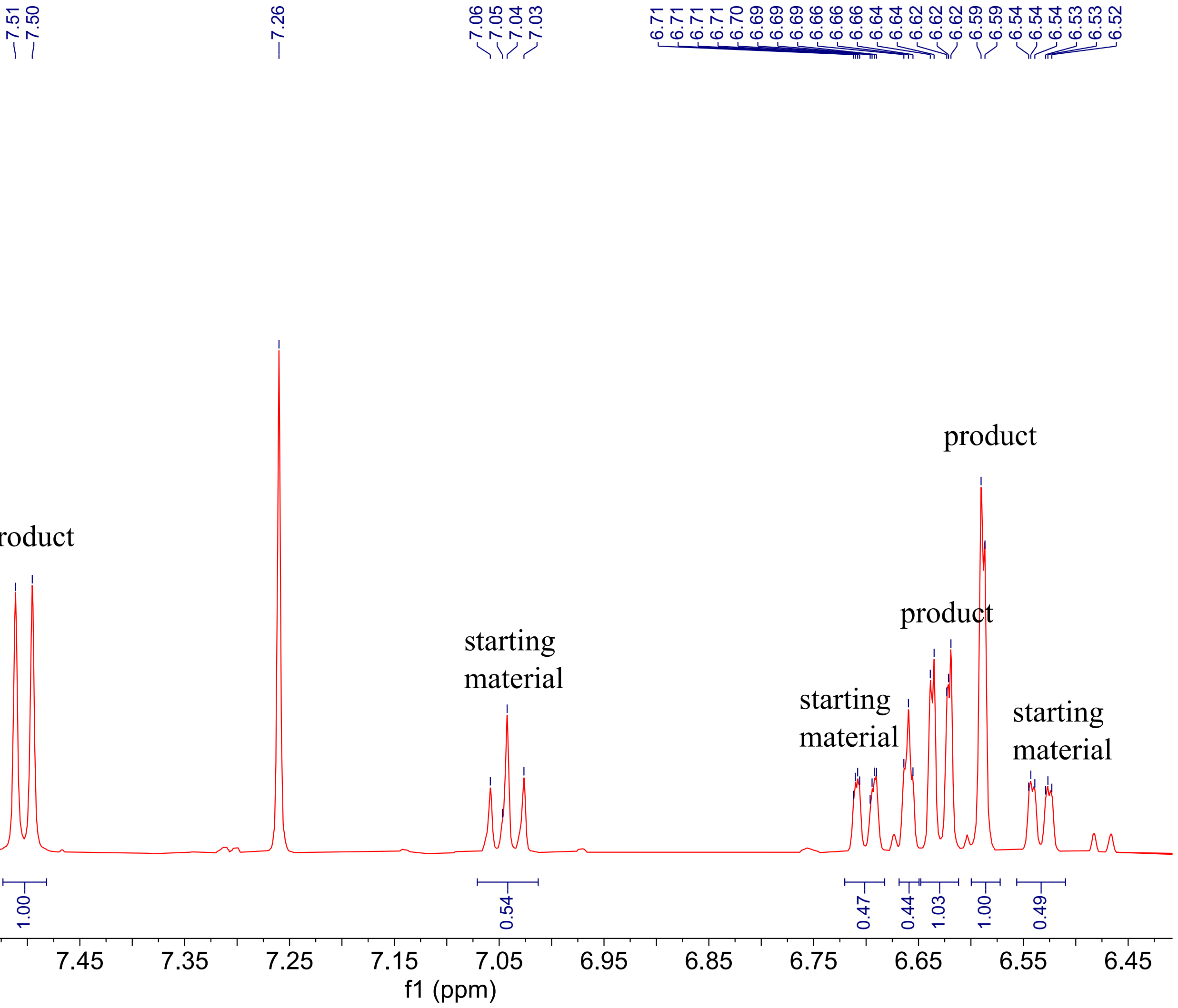

^{1}H NMR of *ortho* Bbg-borylated 3-chloroaniline (7) ($CDCl_3$, 500 MHz)

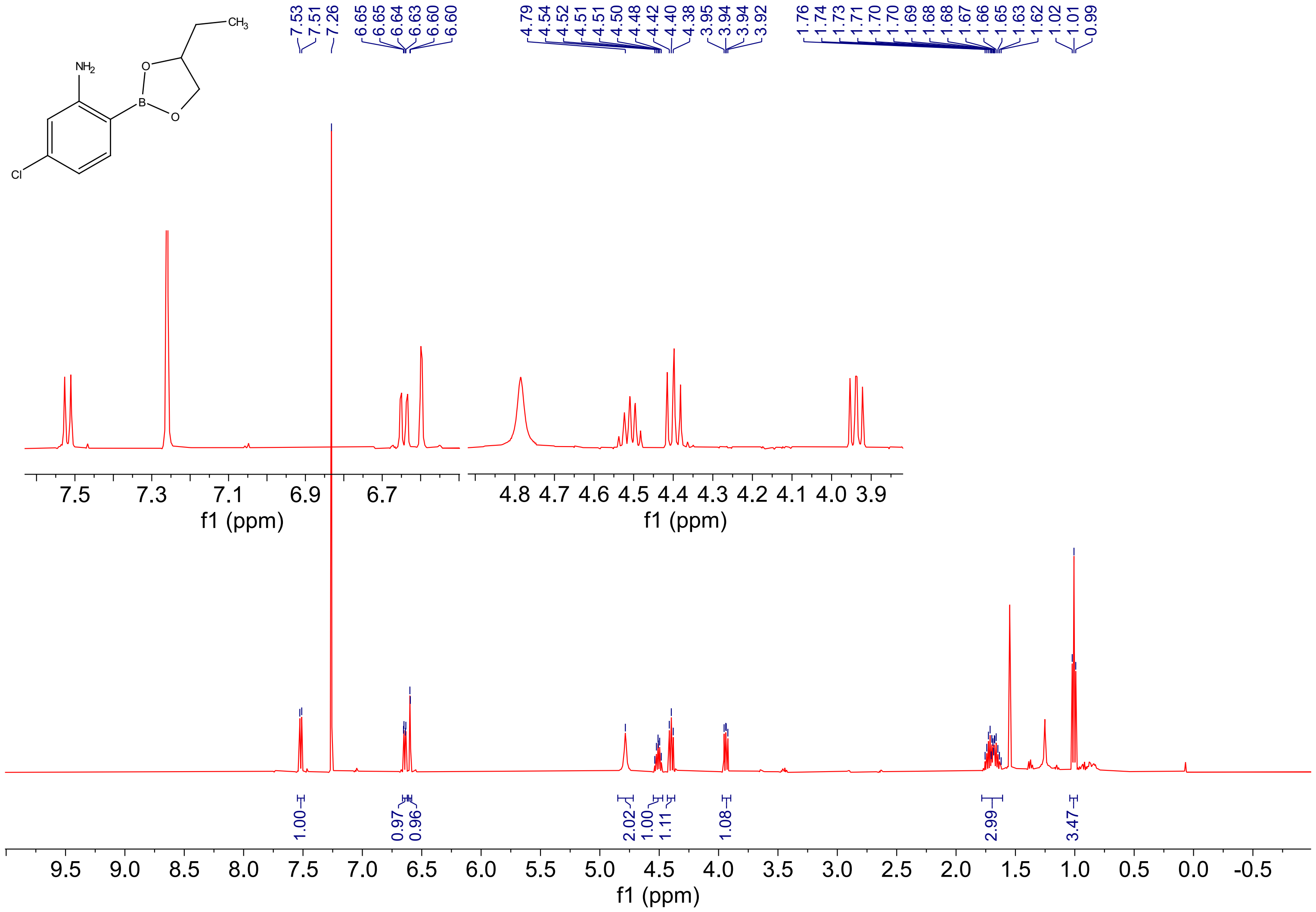

$^{13}C\{^1H\}$ NMR of *ortho* Bbg-borylated 3-chloroaniline (7) ($CDCl_3$, 160 MHz)

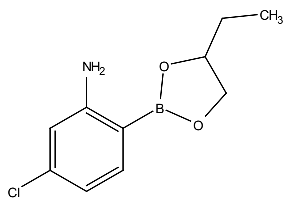

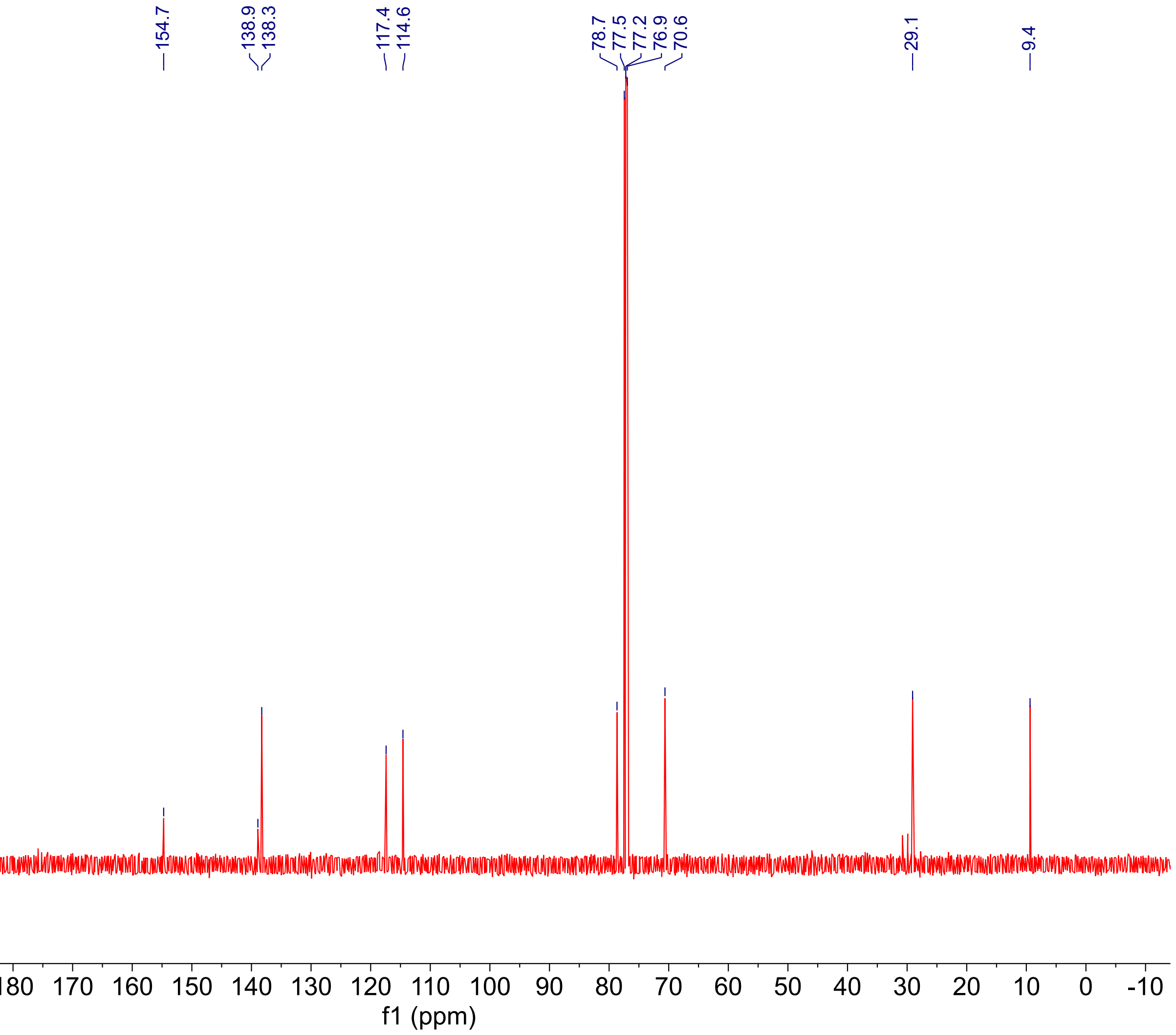

^{11}B NMR of *ortho* Bbg-borylated 3-chloroaniline (7) ($CDCl_3$, 126 MHz)

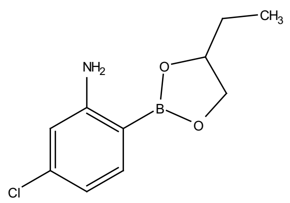

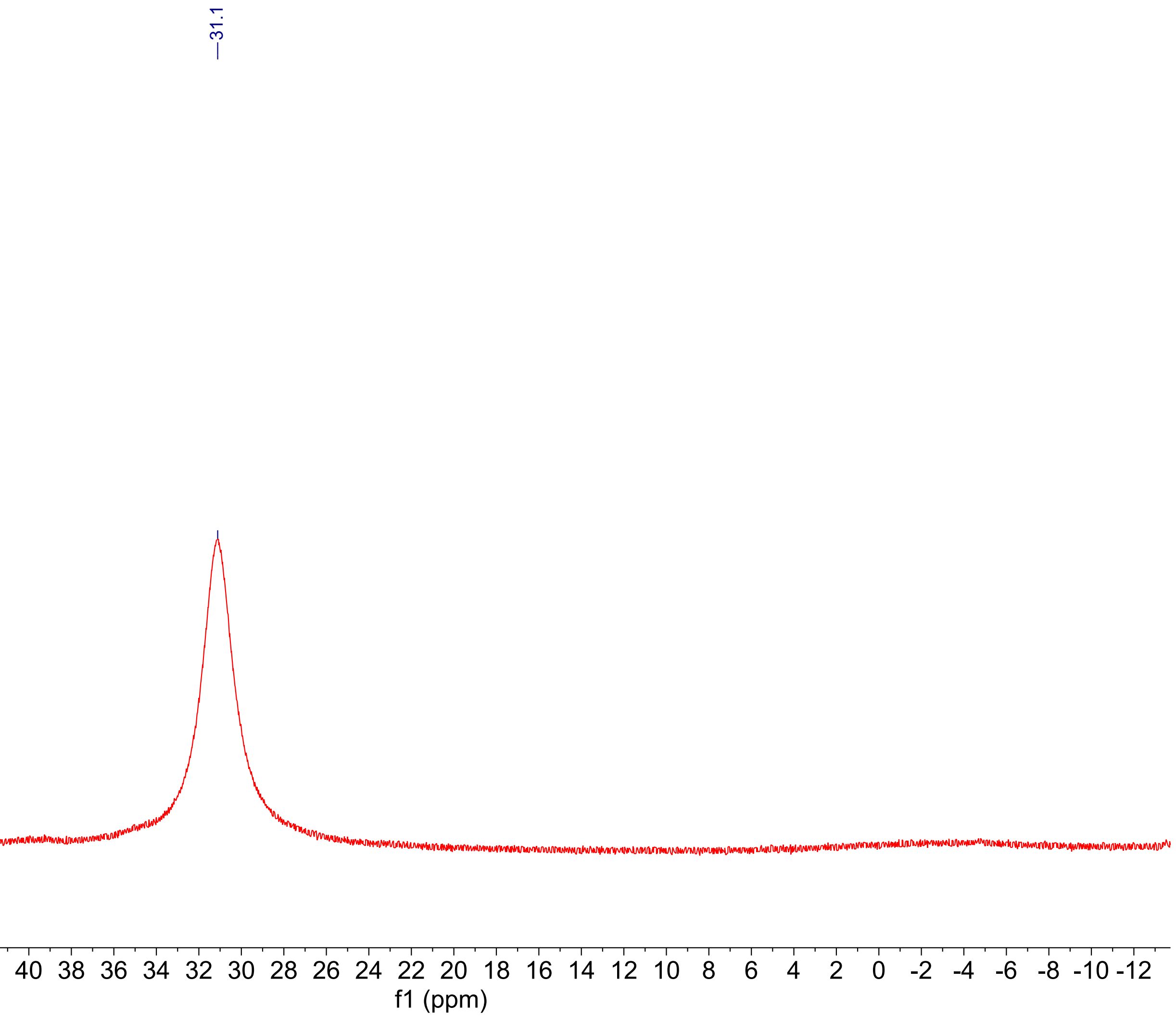

^{1}H NMR spectrum of the reaction mixture of CHB 3-(trifluoromethyl)aniline (8) ($CDCl_3$, 500 MHz)

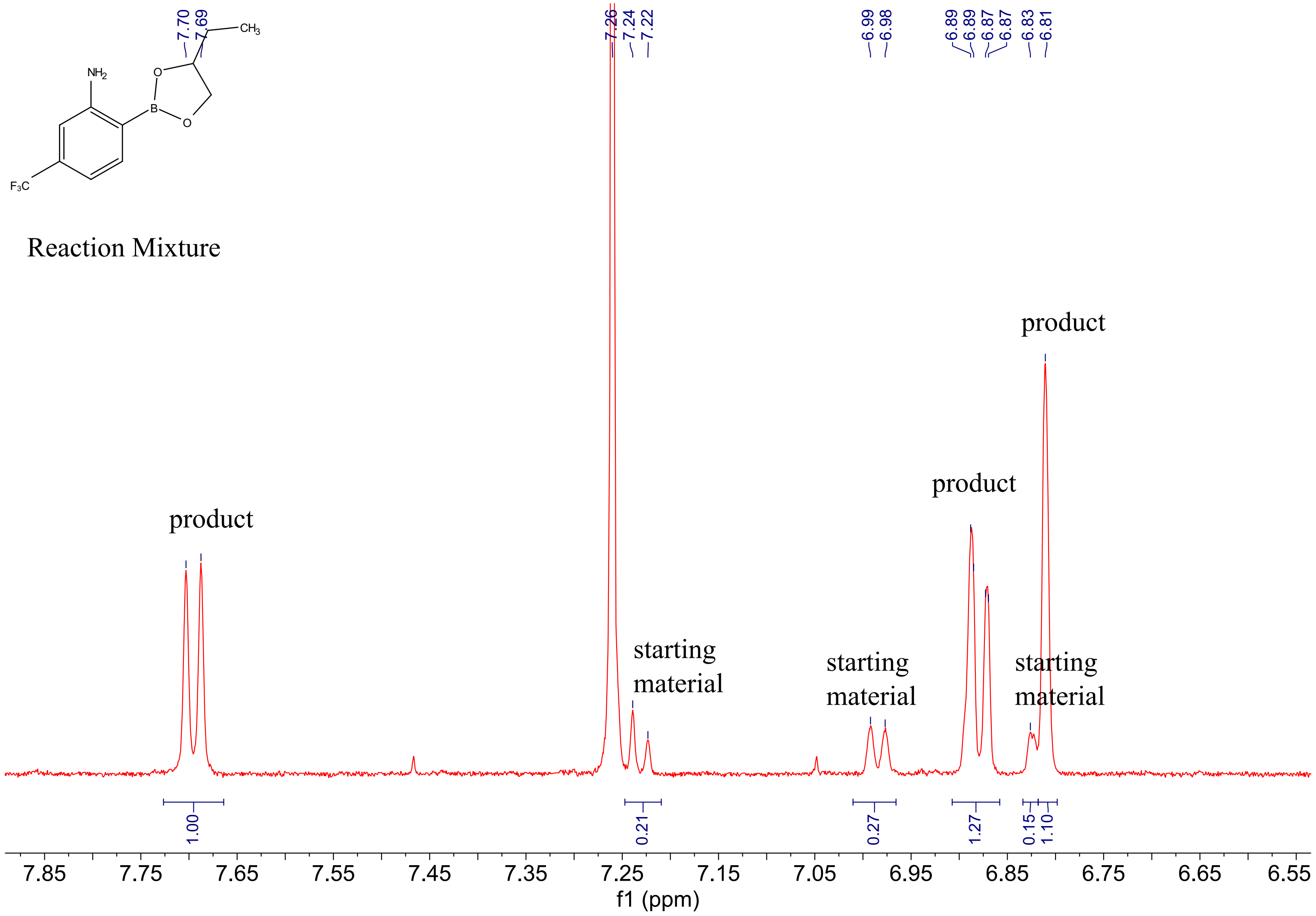

^{1}H NMR of *ortho* Bbg-borylated 3-(trifluoromethyl)aniline (8) ($CDCl_3$, 500 MHz)

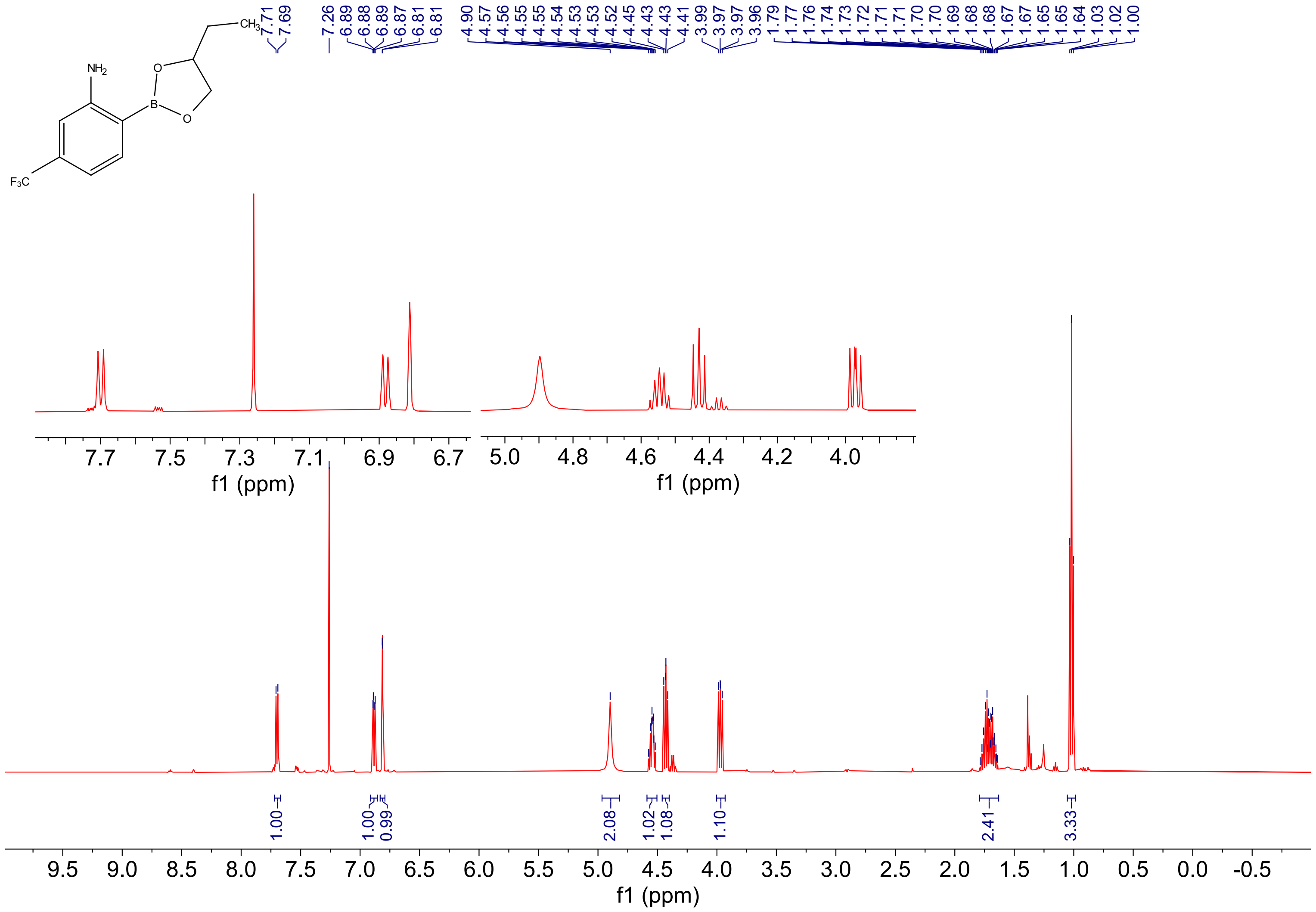

$^{13}C\{^{1}H\}$ **NMR of *ortho* Bbg-borylated 3-(trifluoromethyl)aniline (8) ($CDCl_3$, 160 MHz)**

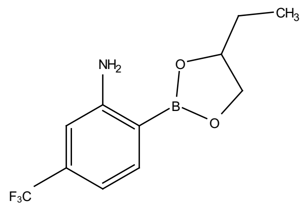

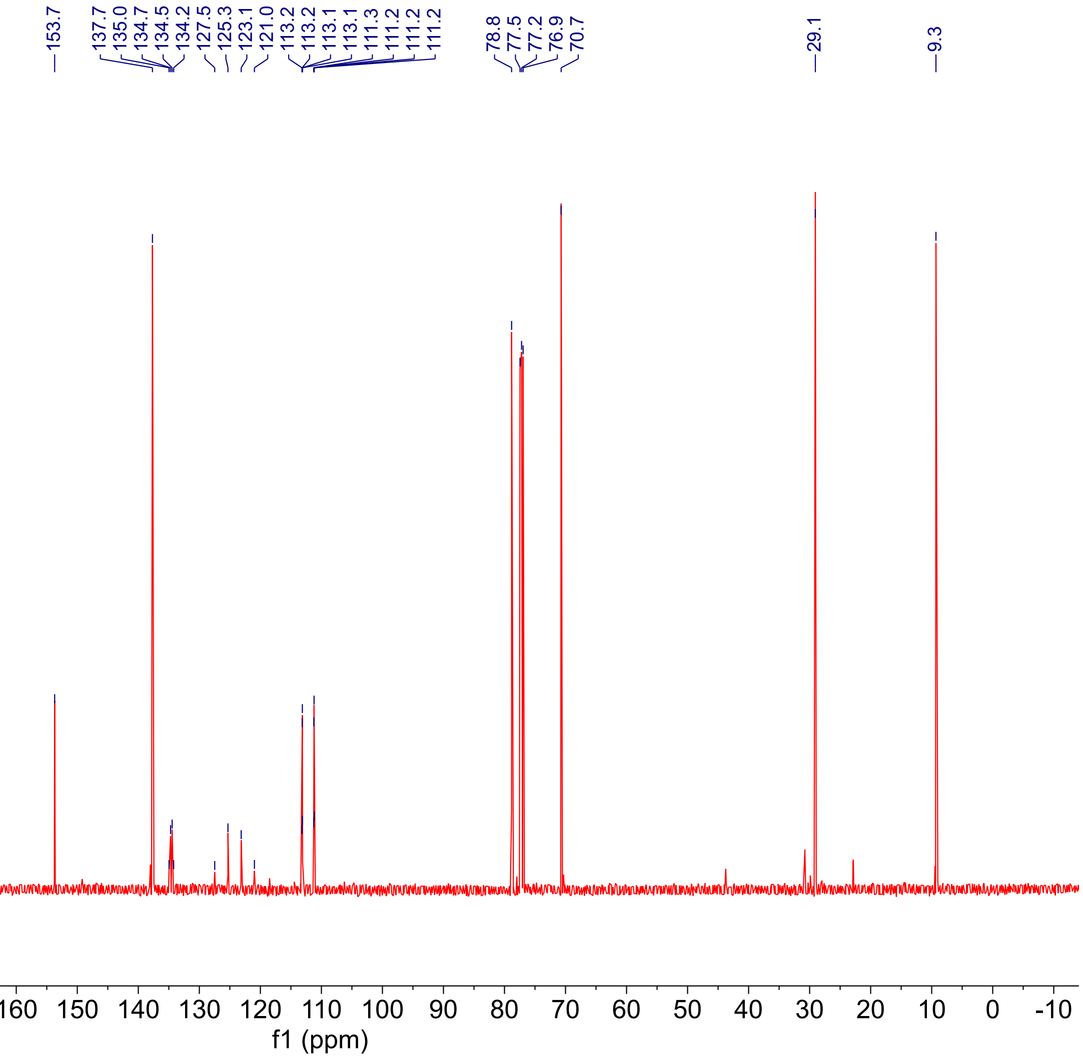

^{11}B NMR of *ortho* Bbg-borylated 3-(trifluoromethyl)aniline (8) ($CDCl_3$, 126 MHz)

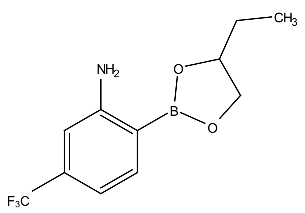

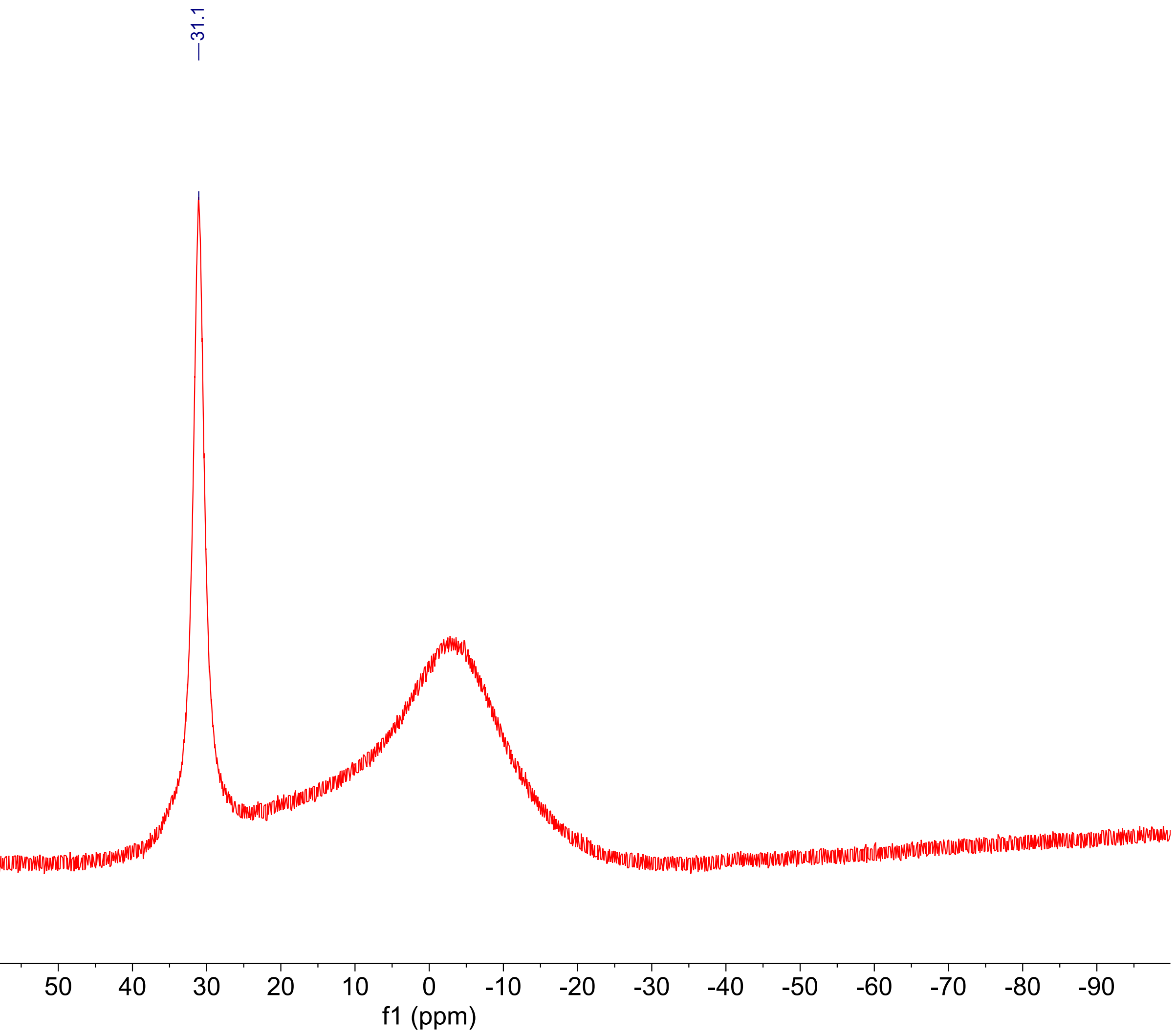

^{19}F NMR of *ortho* Bbg-borylated 3-(trifluoromethyl)aniline (8) ($CDCl_3$, 470 MHz)

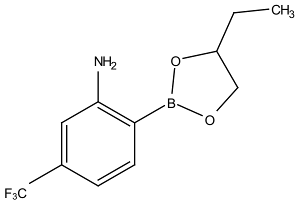

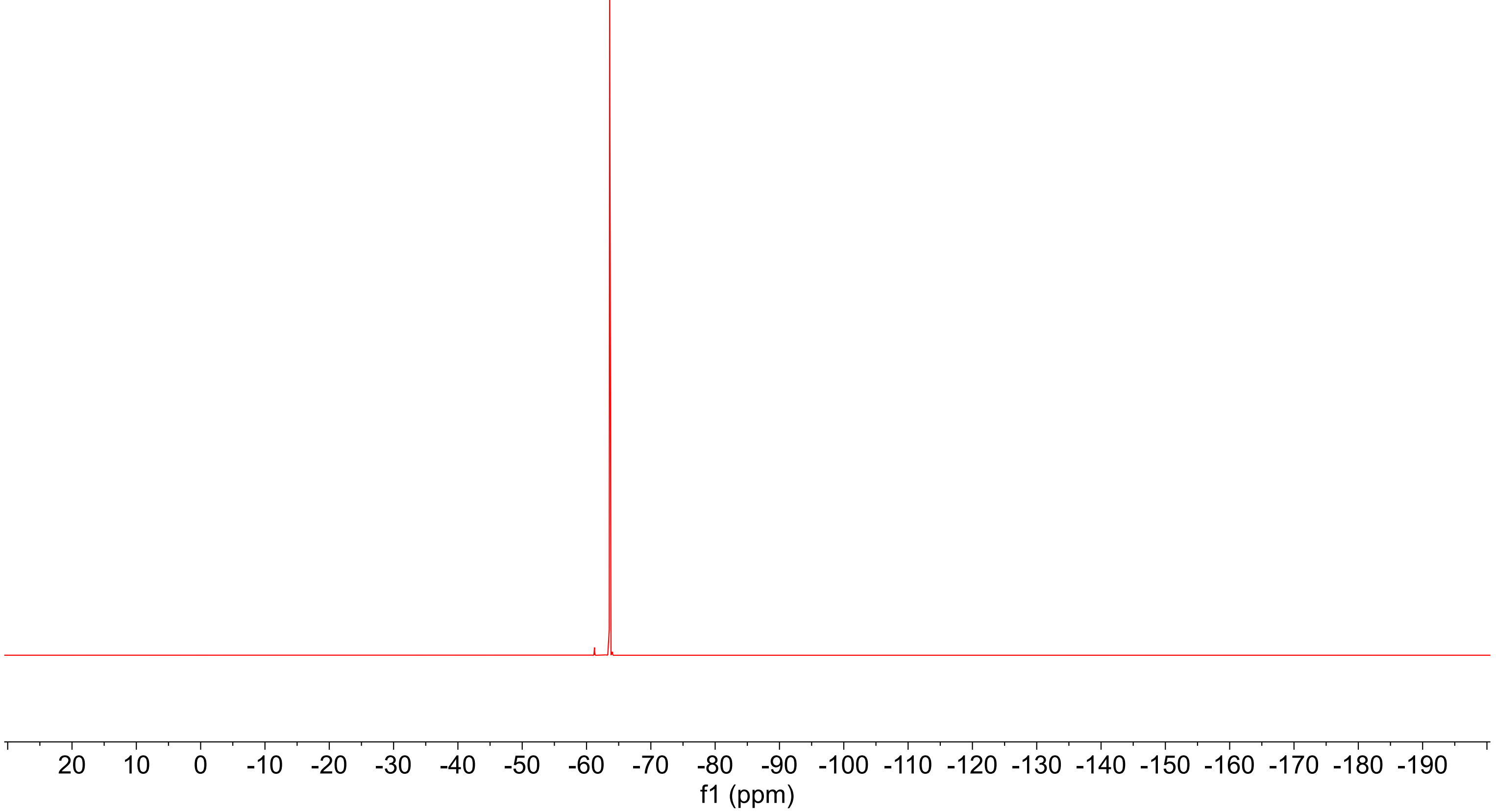

^{1}H NMR spectrum of the reaction mixture of CHB 3-methylaniline (9) ($CDCl_3$, 500 MHz)

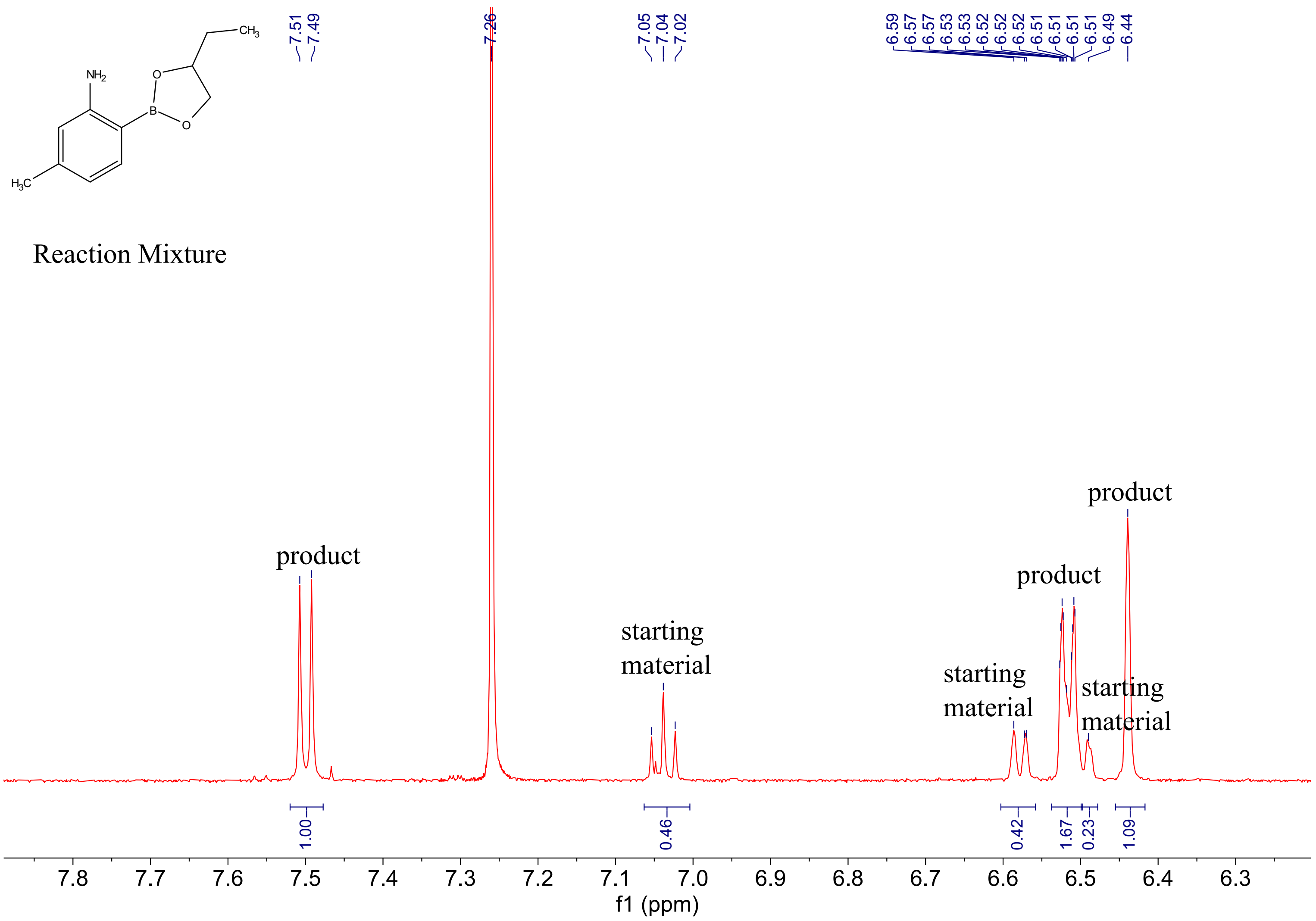

^{1}H NMR of *ortho* Bbg-borylated 3-methylaniline (9) ($CDCl_3$, 500 MHz)

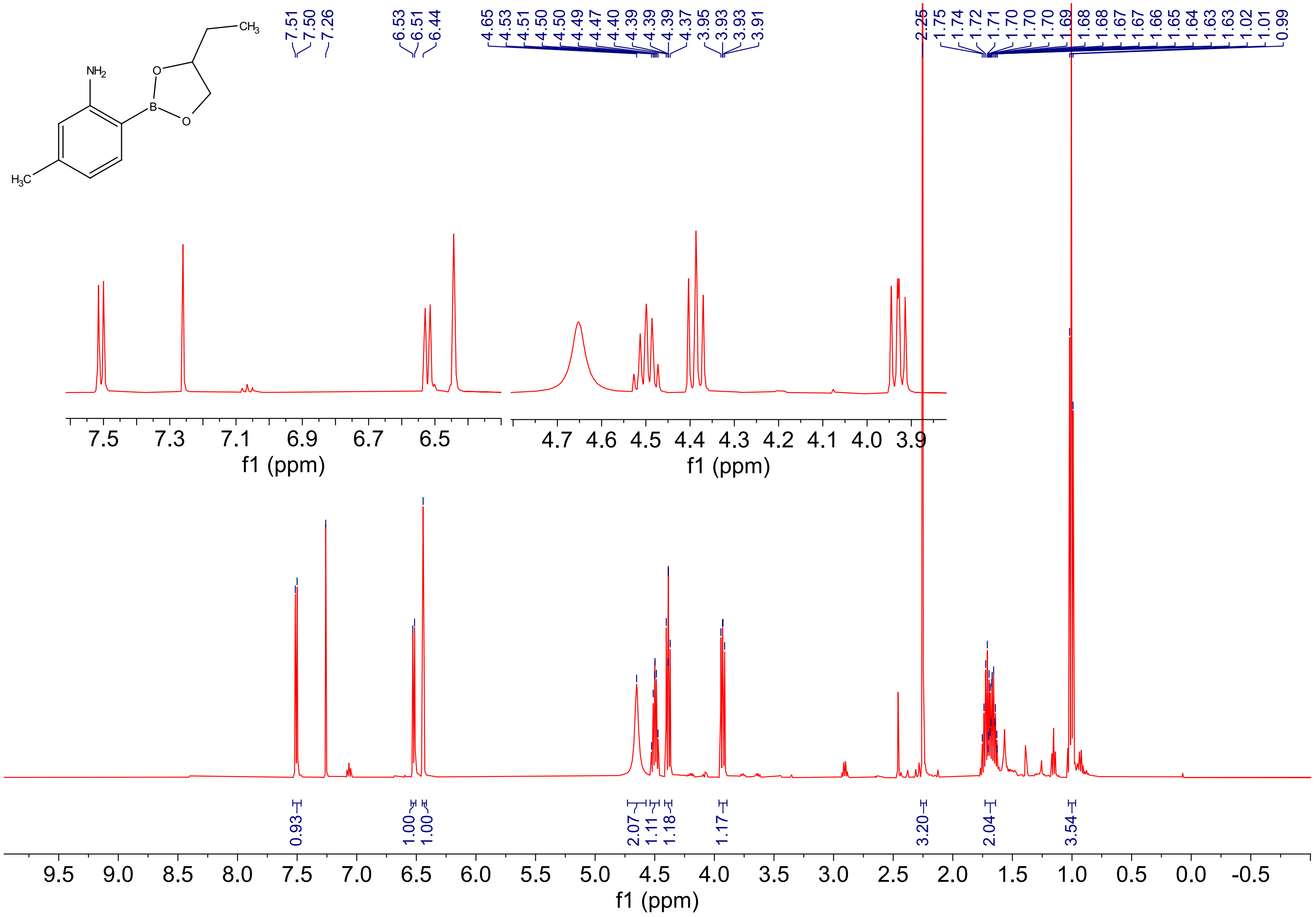

$^{13}C\{^1H\}$ NMR of *ortho* Bbg-borylated 3-methylaniline (9) ($CDCl_3$, 160 MHz)

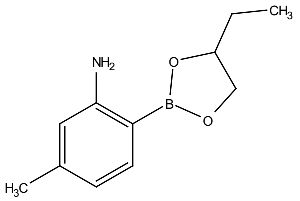

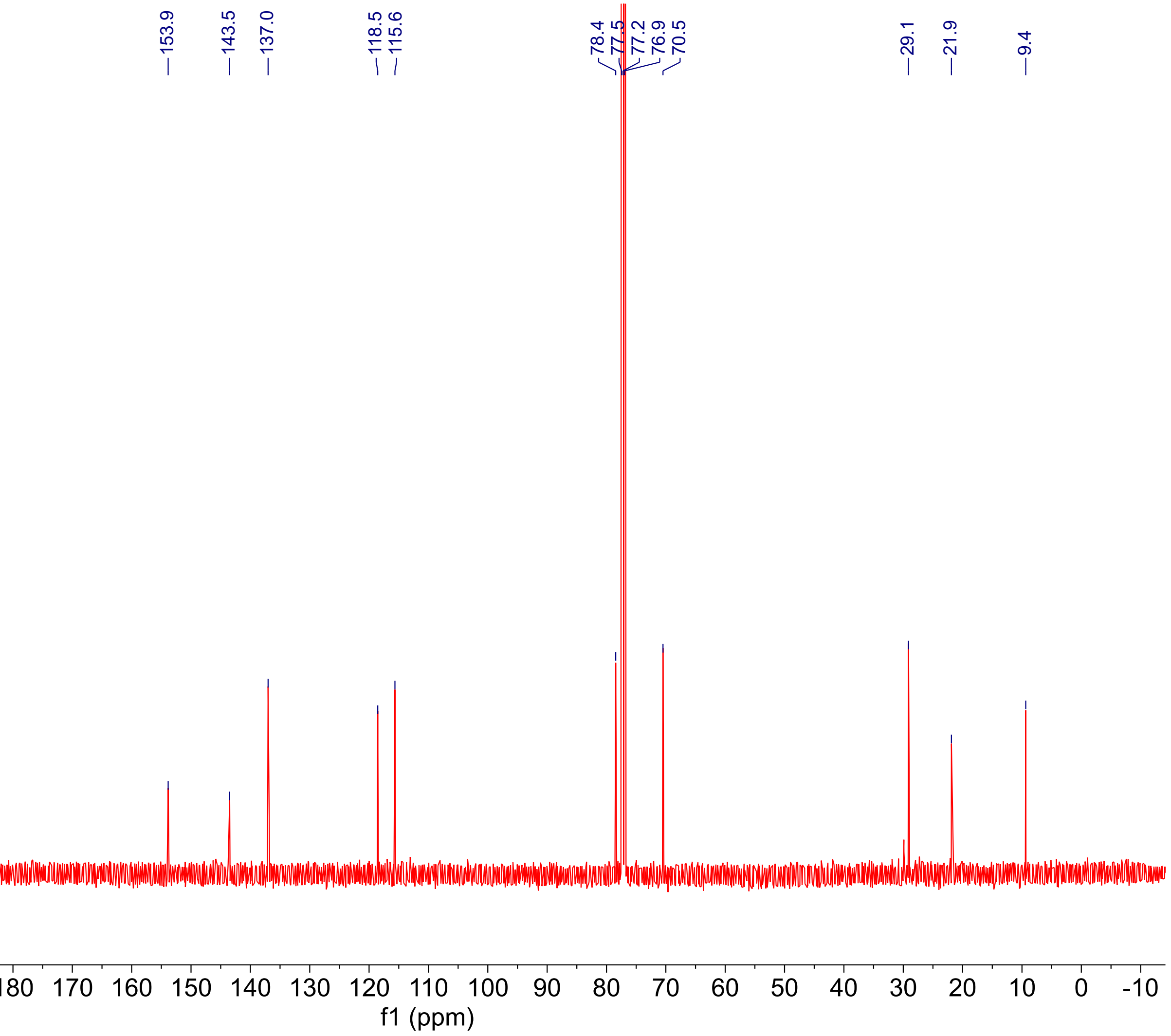

^{11}B NMR of *ortho* Bbg-borylated 3-methylaniline (9) ($CDCl_3$, 126 MHz)

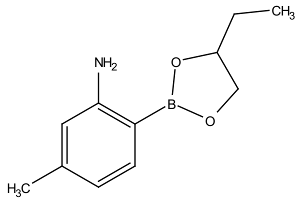

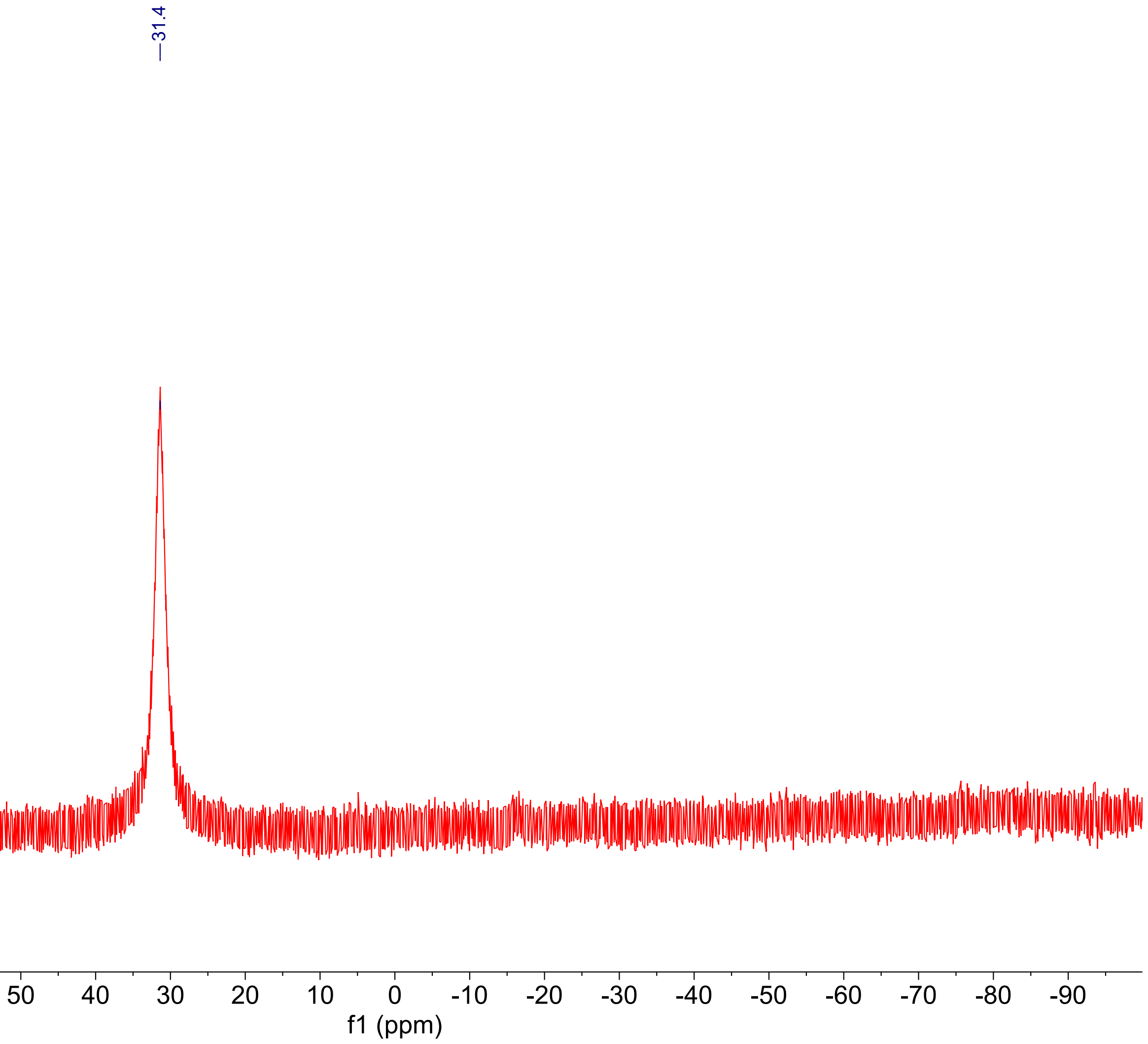

^{1}H NMR spectrum of the reaction mixture of CHB 3-methoxyaniline (10) ($CDCl_3$, 500 MHz)

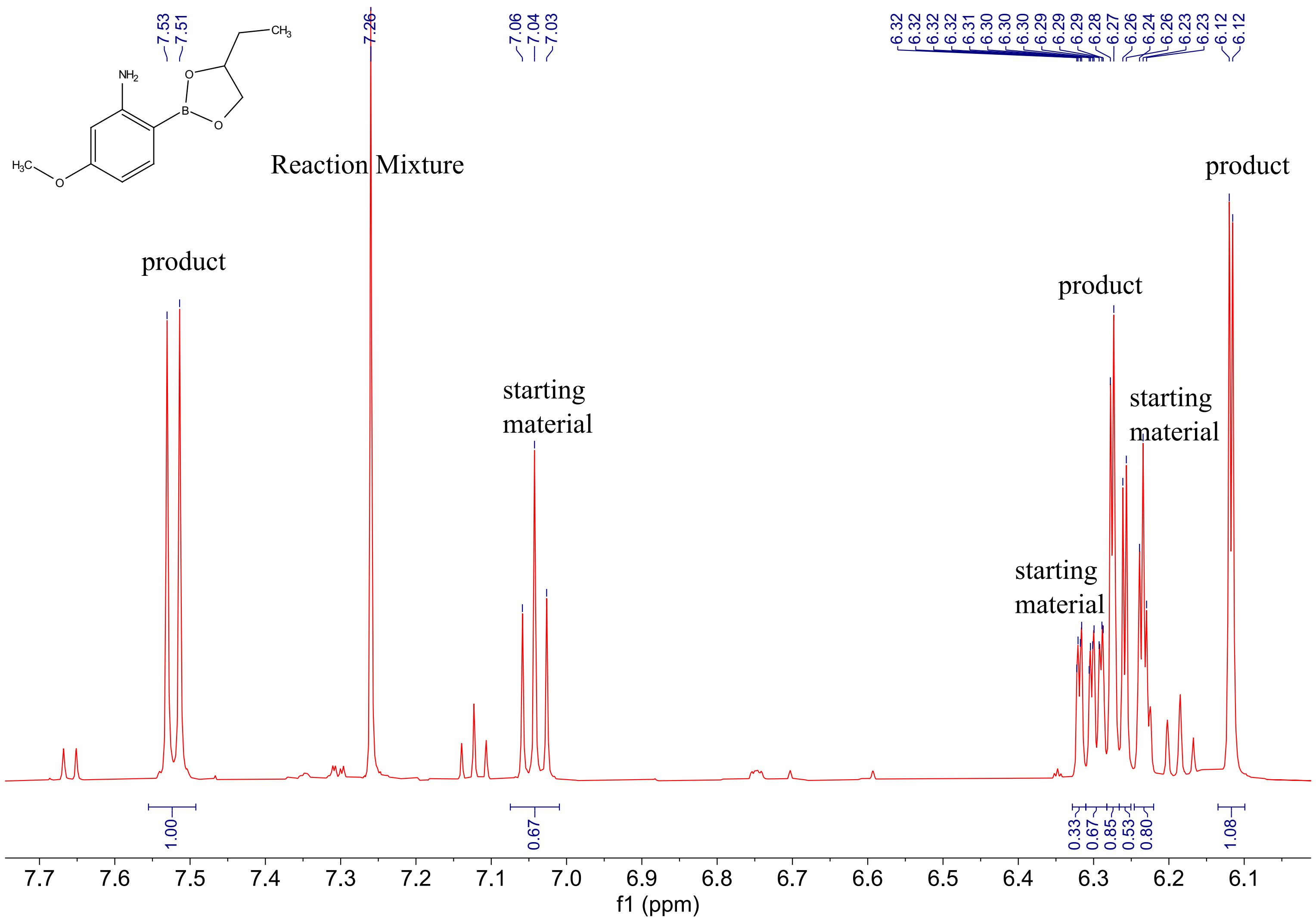

^{1}H NMR of *ortho* Bbg-borylated 3-methoxyaniline (10) ($CDCl_3$, 500 MHz)

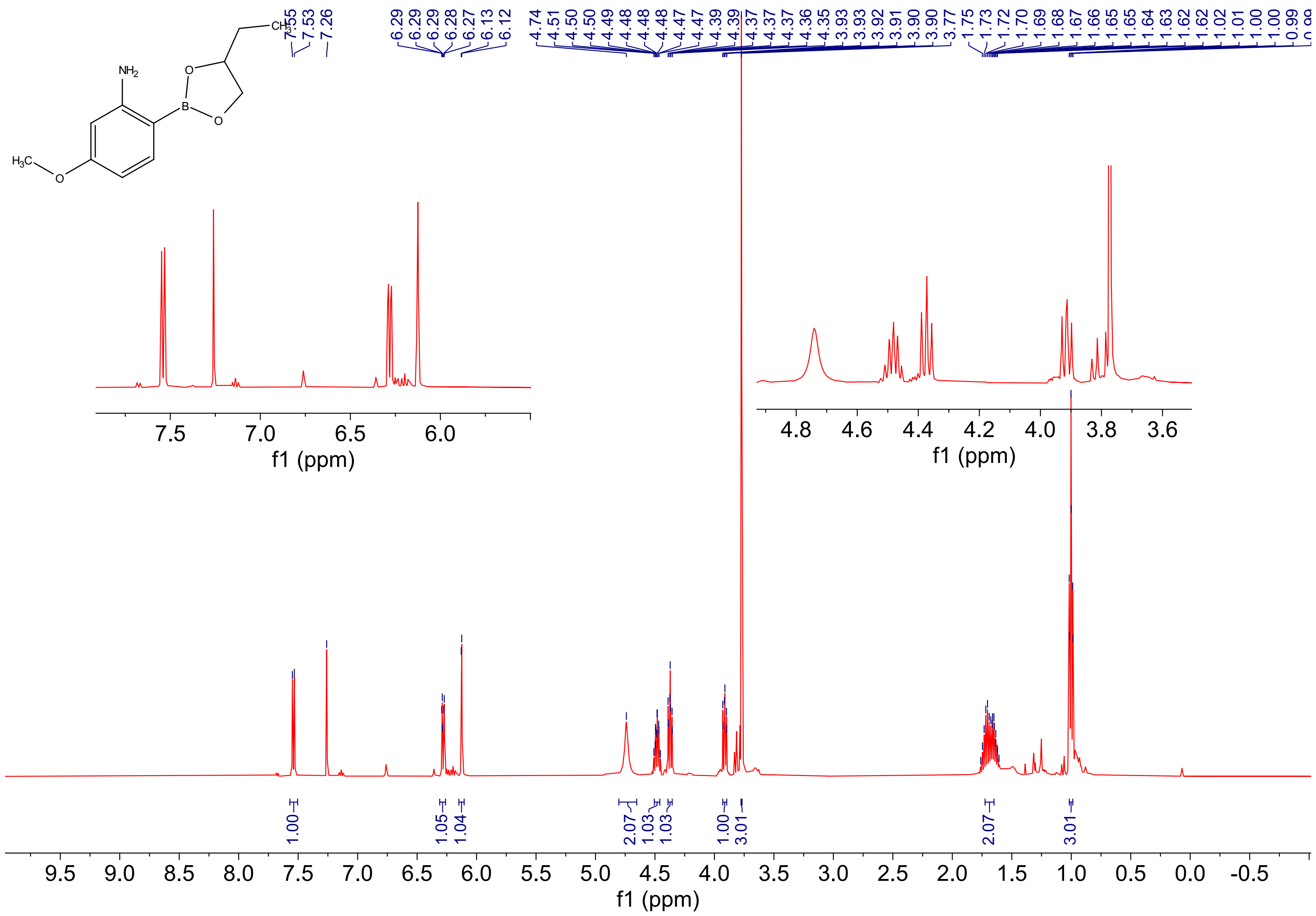

$^{13}C\{^1H\}$ NMR of *ortho* Bbg-borylated 3-methoxyaniline (10) ($CDCl_3$, 160 MHz)

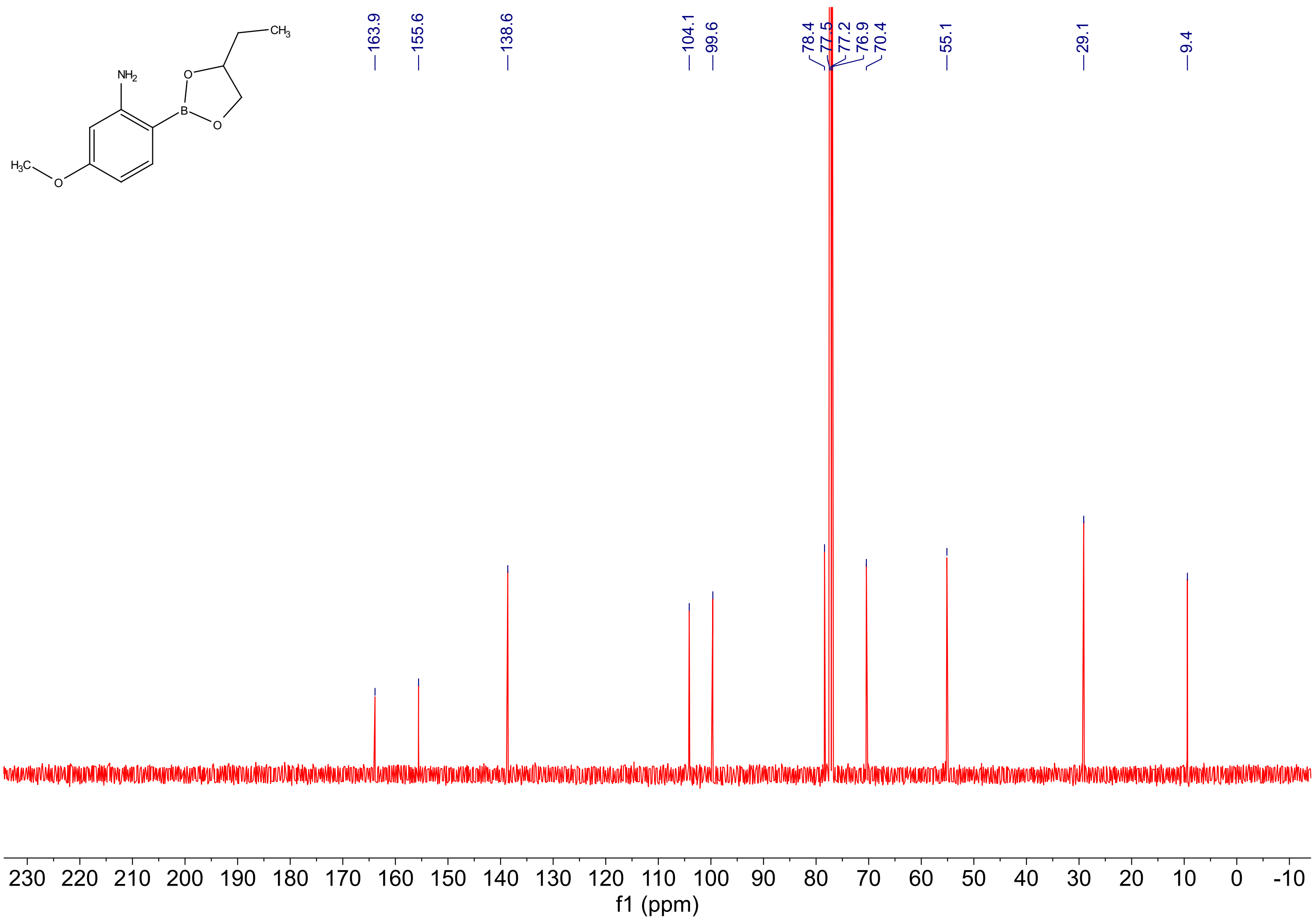

^{11}B NMR of *ortho* Bbg-borylated 3-methoxyaniline (10) ($CDCl_3$, 126 MHz)

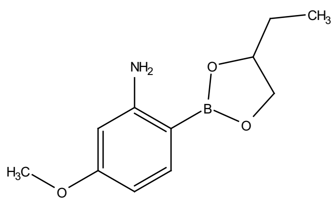

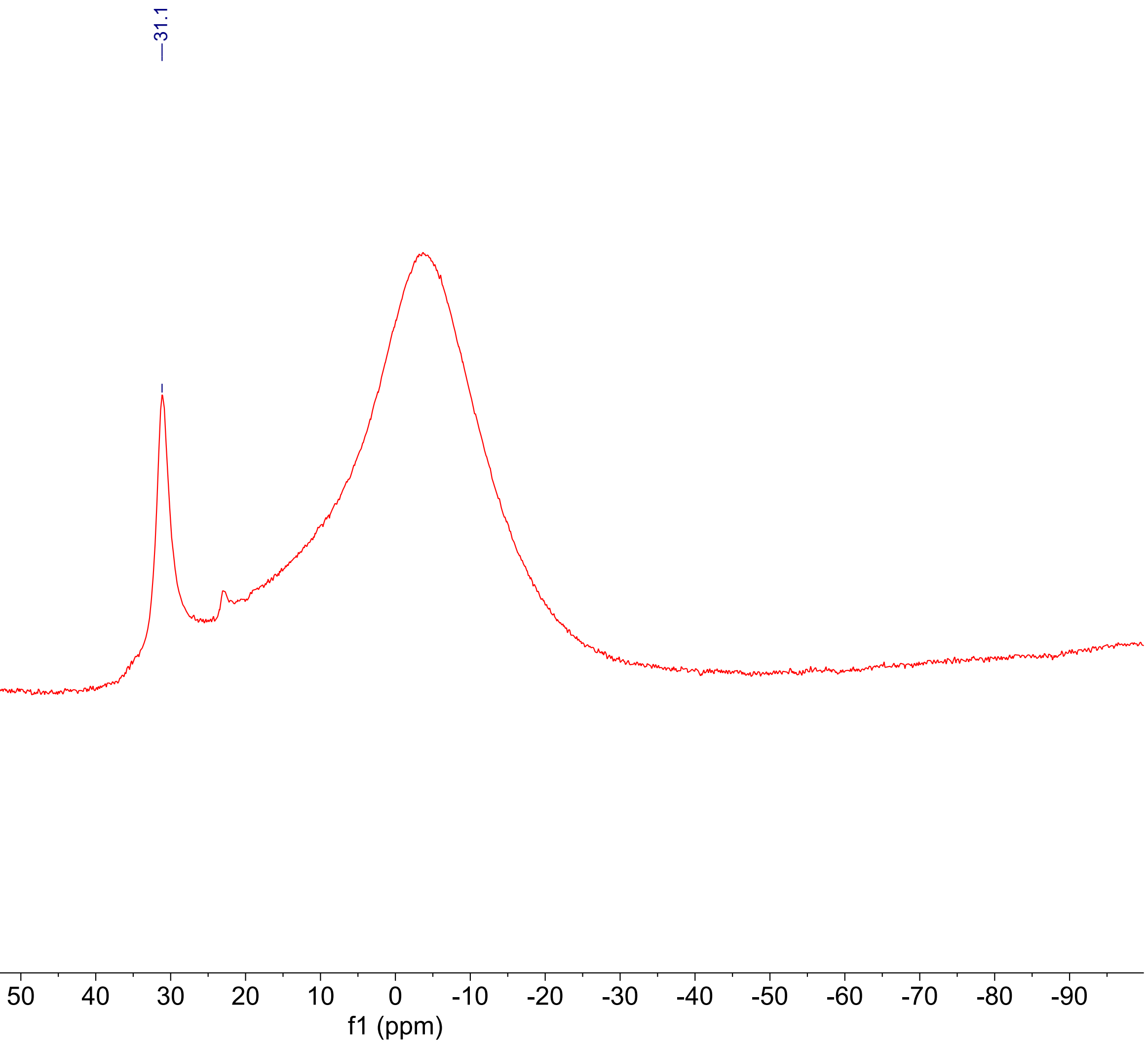

^{1}H NMR spectrum of the reaction mixture of CHB 3-fluoroaniline (11) ($CDCl_3$, 500 MHz)

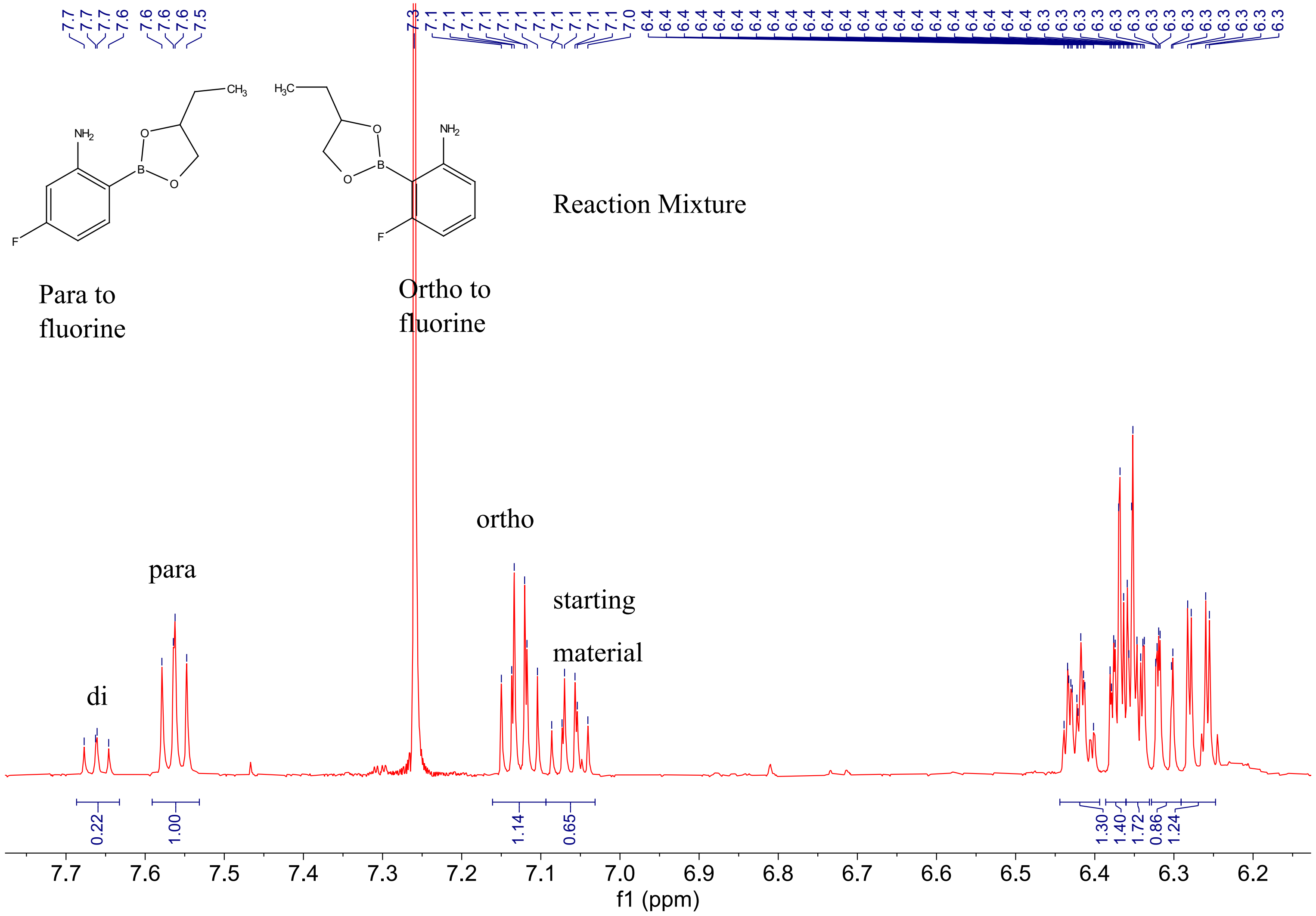

^{1}H NMR of *ortho* Bbg-borylated 3-fluoroaniline (11.1) ($CDCl_3$, 500 MHz) (Isolation done using silica gel)

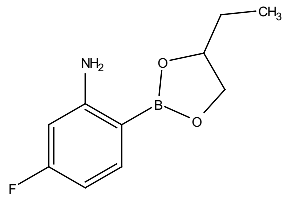

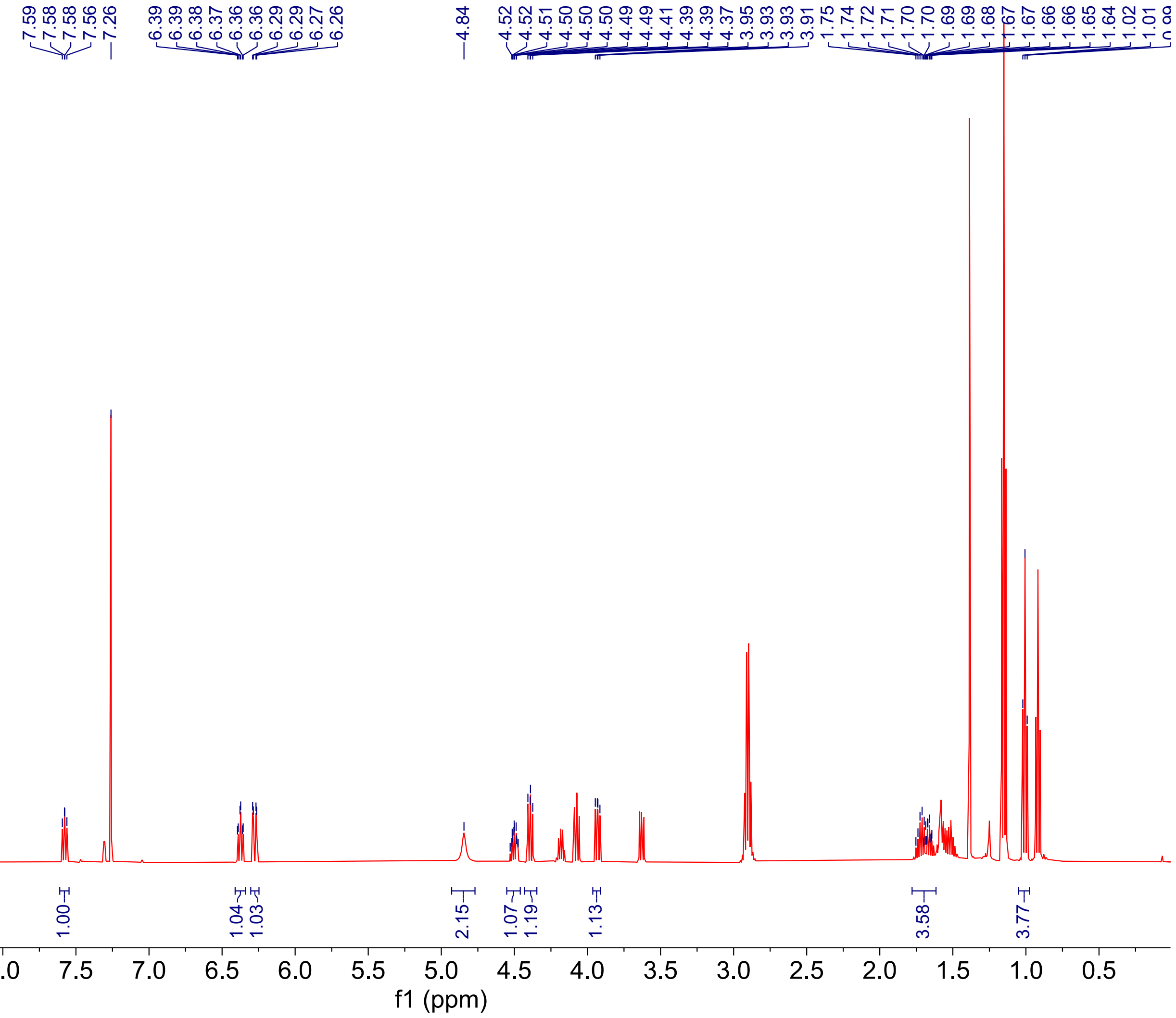

^{1}H NMR of *ortho* Bbg-borylated 3-fluoroaniline (11.1 + 11.2) ($CDCl_3$, 500 MHz) (Isolation done using neutral alumina gel)

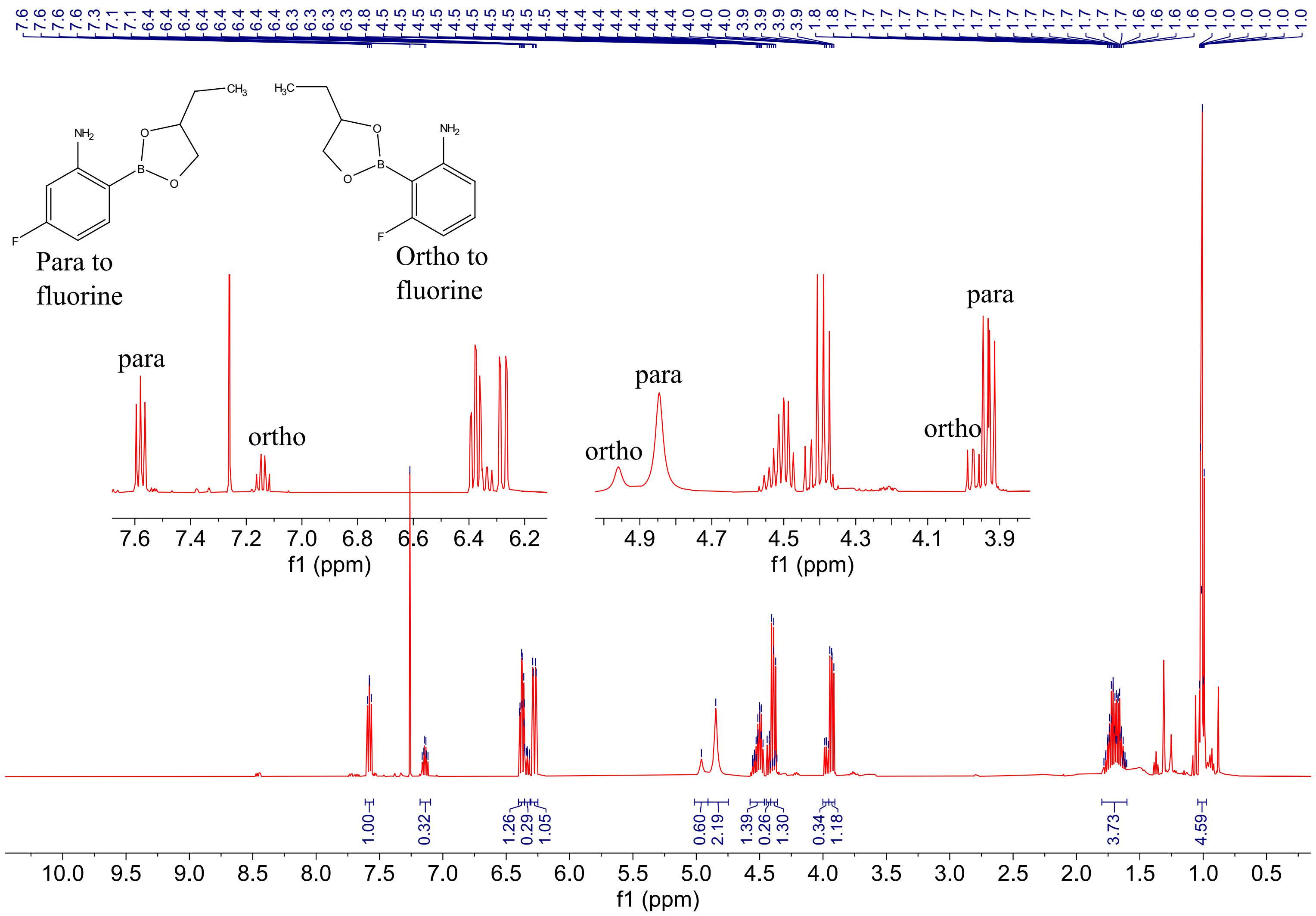

^{1}H NMR of *ortho* Bbg-borylated 3-fluoroaniline (11) ($CDCl_3$, 500 MHz) (Stacked NMR spectrums of reaction mixture, and products isolated by neutral alumina gel and silica gel respectively)

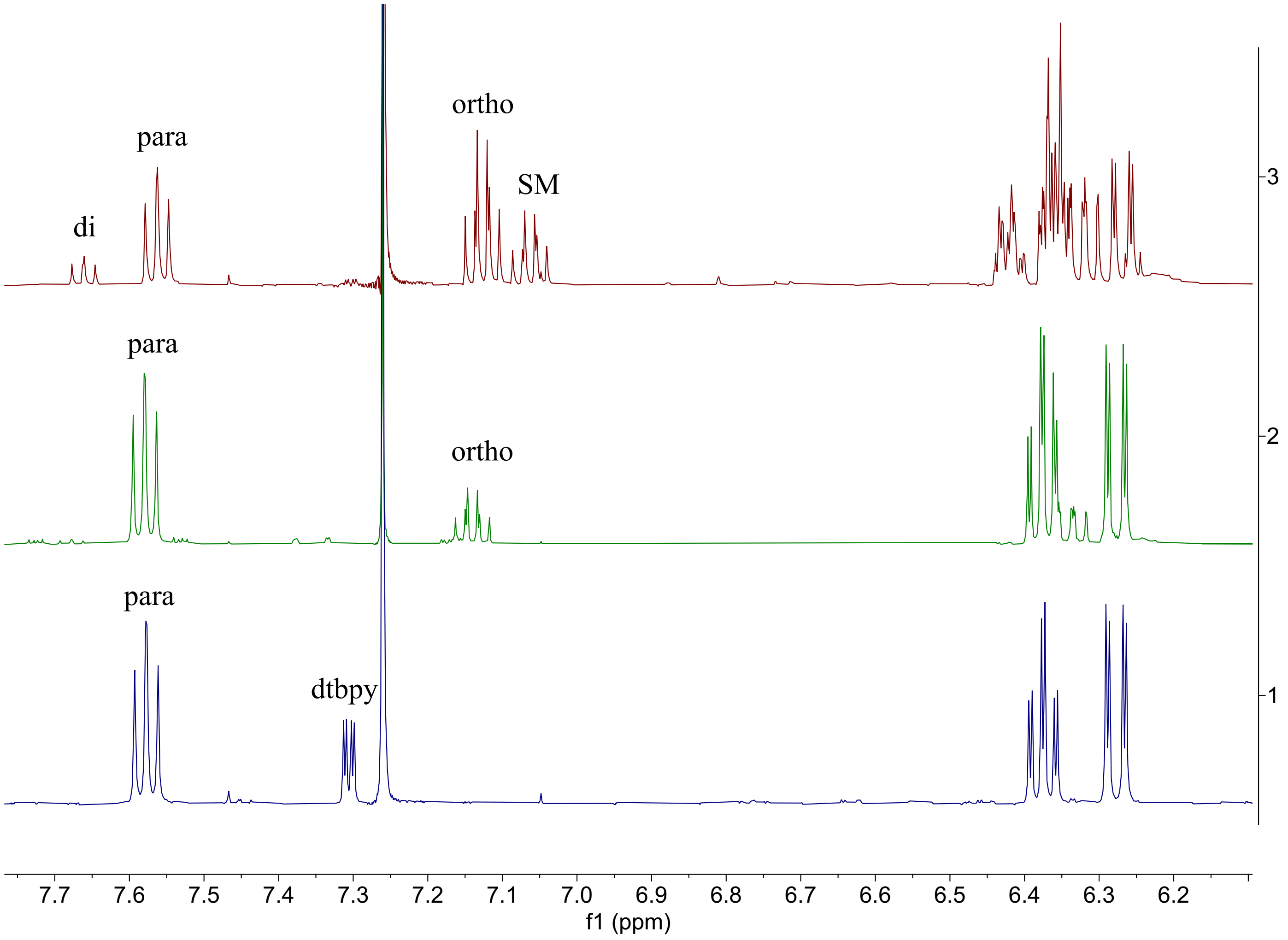

NMR spectrum of crude reaction mixture

NMR spectrum of isolated product by neutral alumina gel

NMR spectrum of isolated product by silica gel

$^{13}C\{^{1}H\}$ NMR of *ortho* Bbg-borylated 3-fluoroaniline (11.1 + 11.2) ($CDCl_3$, 500 MHz) (Isolation done using neutral alumina gel)

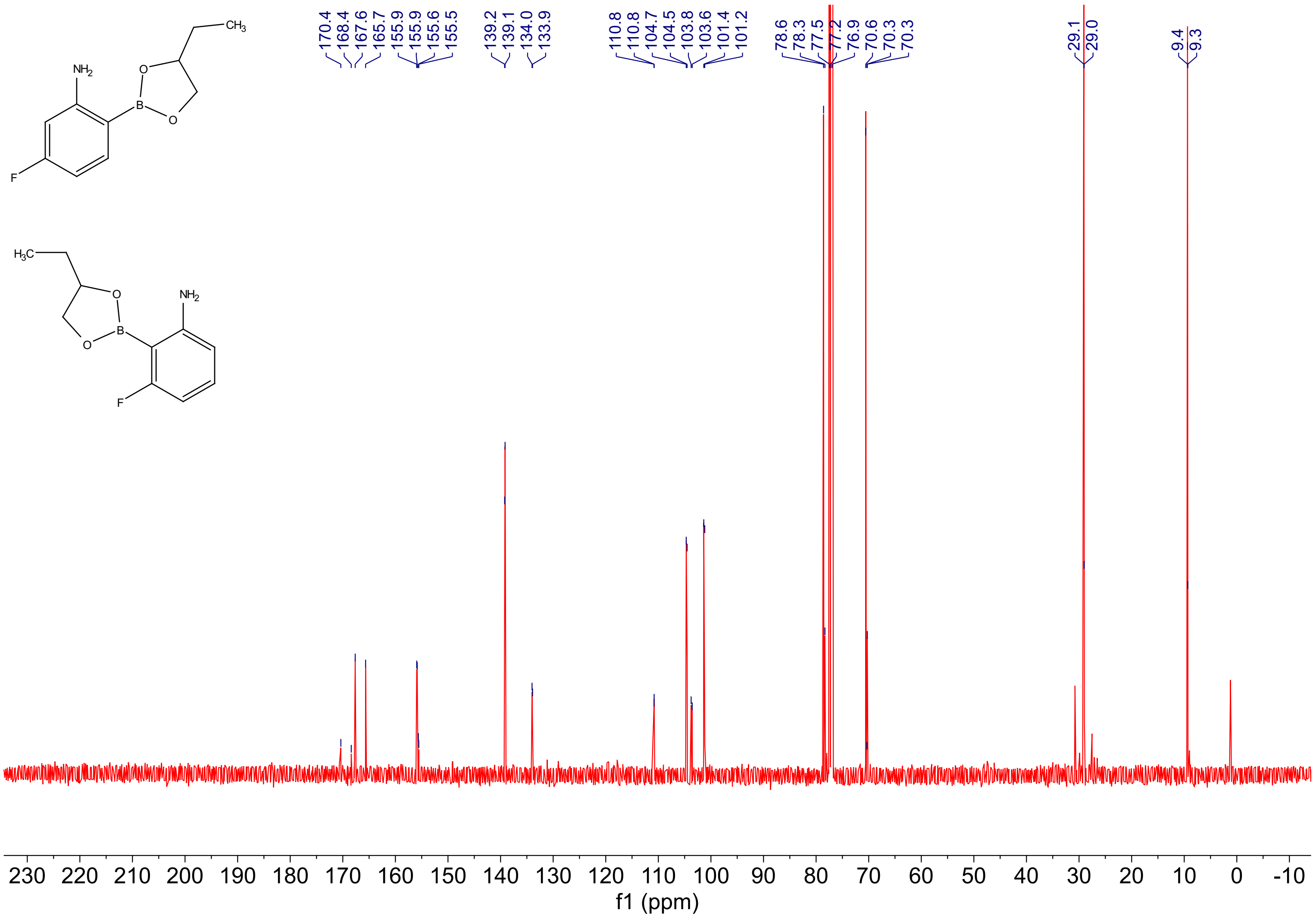

^{11}B NMR of *ortho* Bbg-borylated 3-fluoroaniline (11.1 + 11.2) ($CDCl_3$, 500 MHz) (Isolation done using neutral alumina gel)

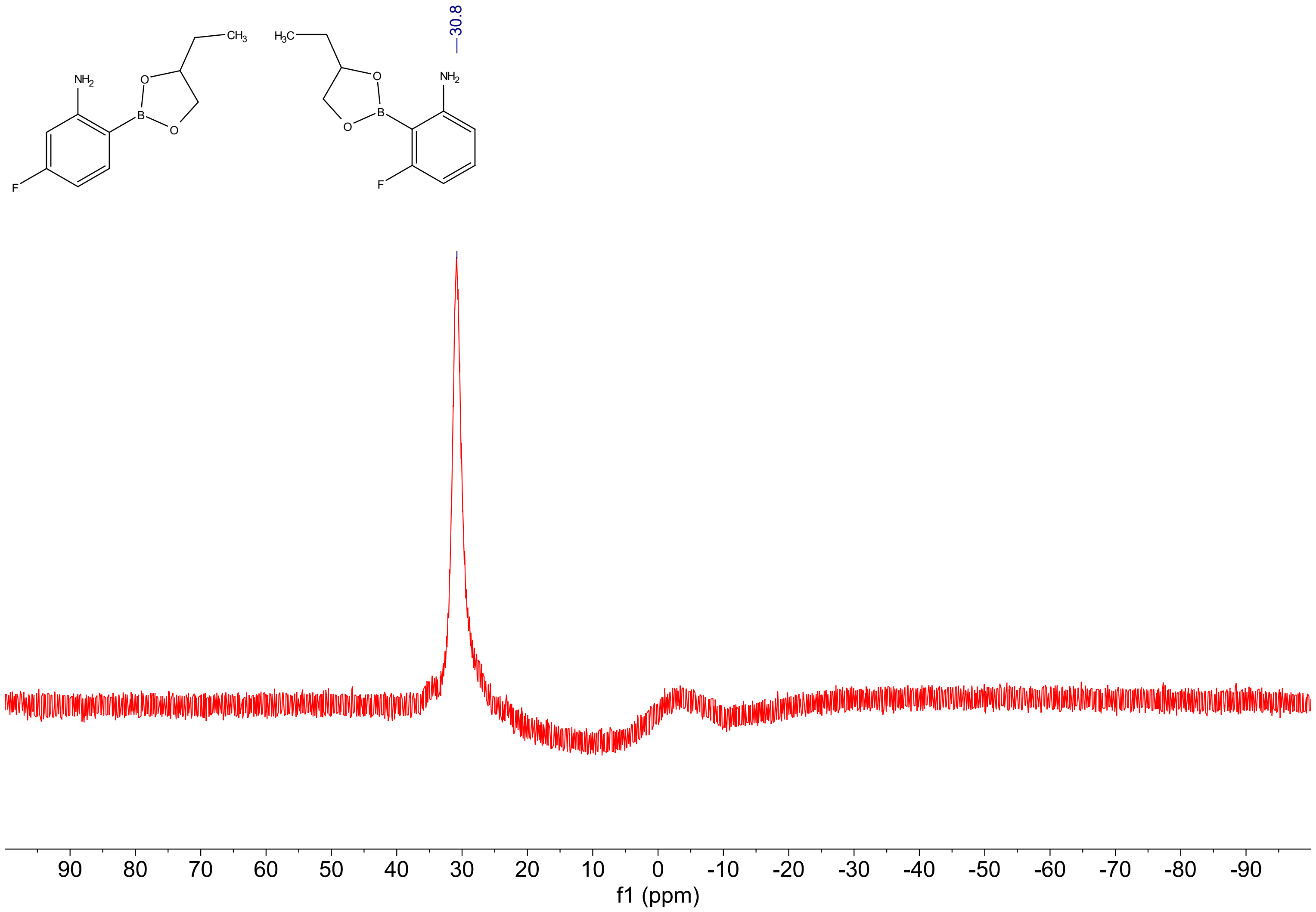

^{19}F NMR of *ortho* Bbg-borylated 3-fluoroaniline (11.1 + 11.2) ($CDCl_3$, 470 MHz) (Isolation done using neutral alumina gel)

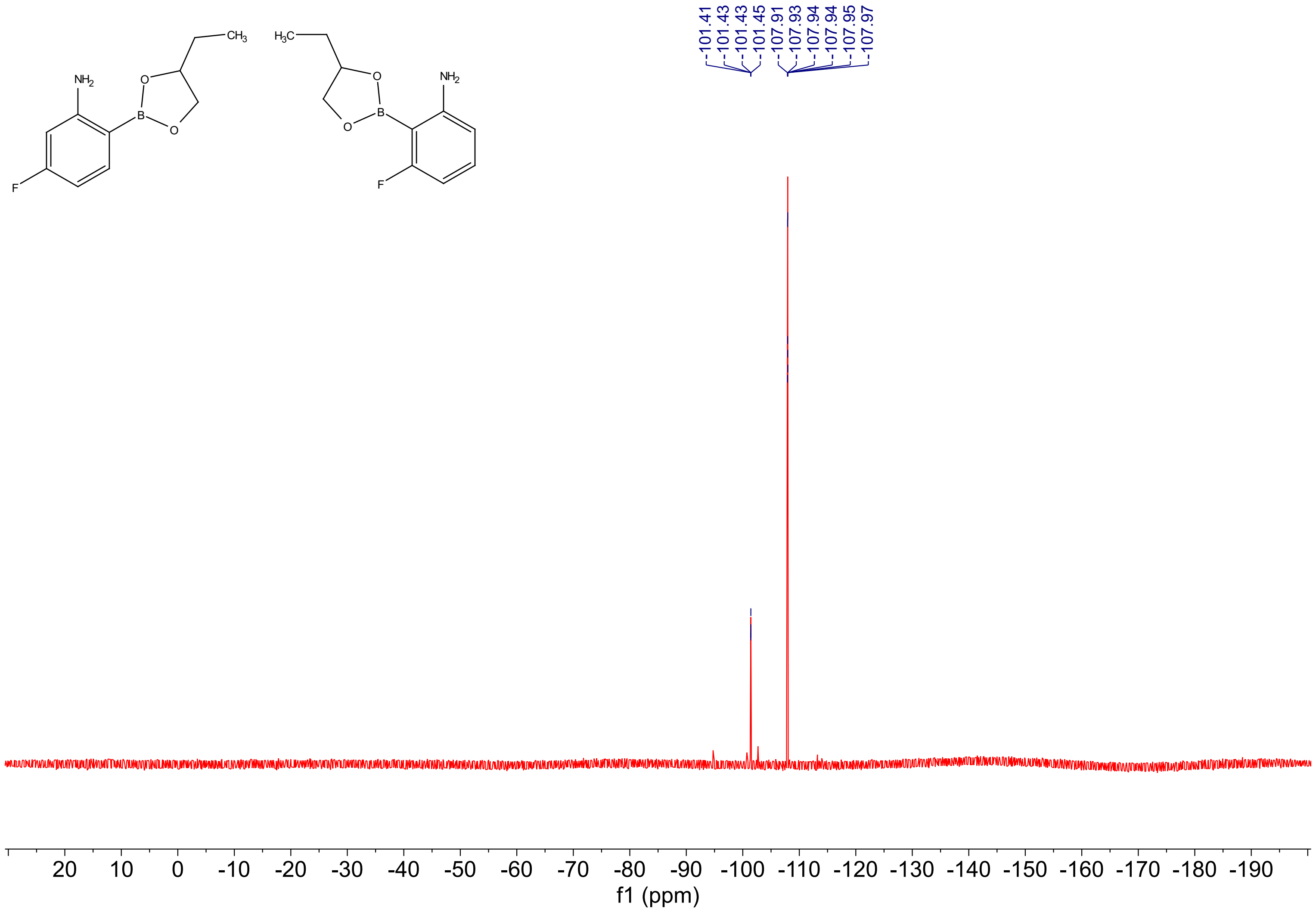

^{1}H NMR spectrum of the reaction mixture of CHB 3-aminobiphenyl (12) ($CDCl_3$, 500 MHz)

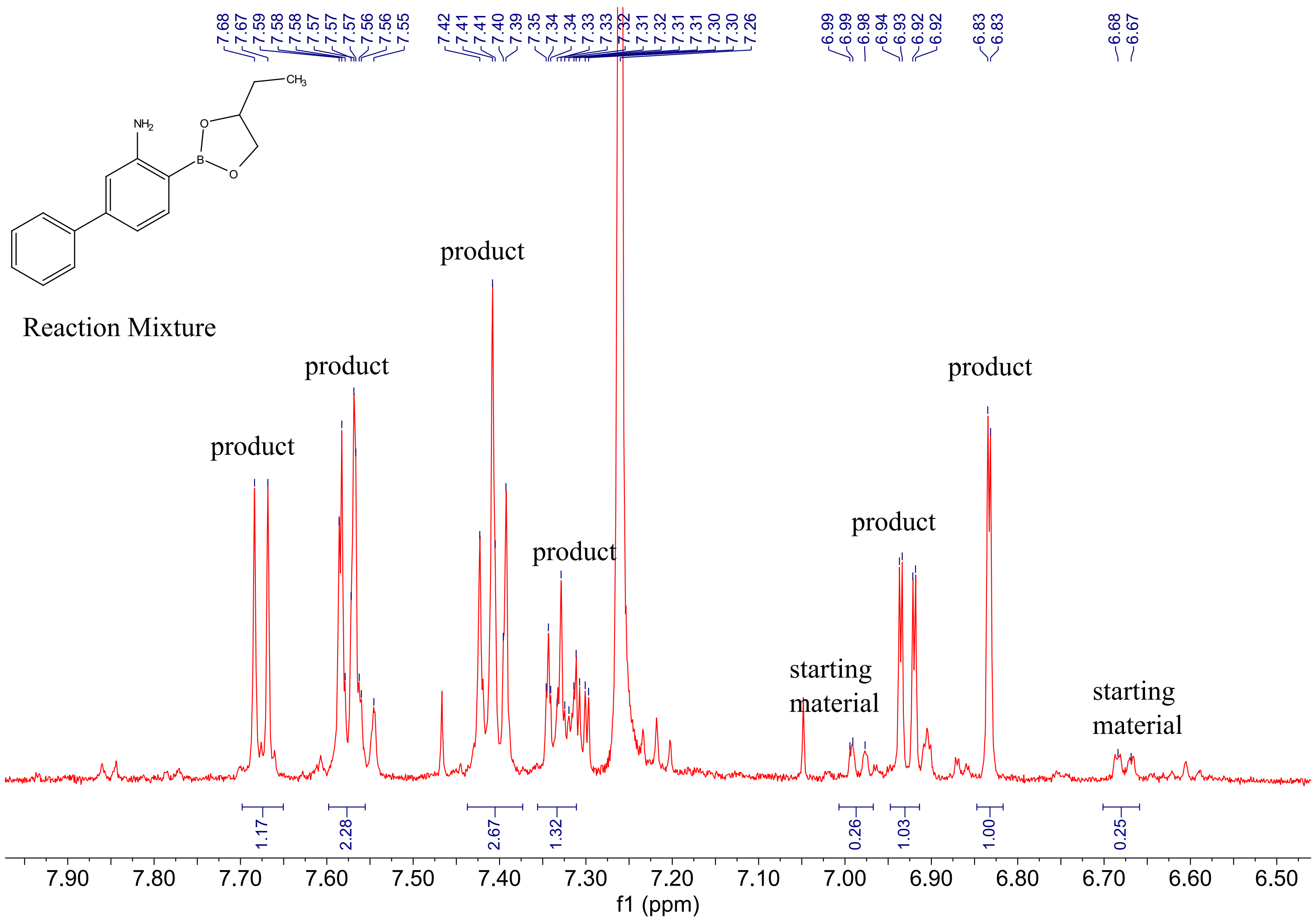

^{1}H NMR of *ortho* Bbg-borylated 3-aminobiphenyl (12) ($CDCl_3$, 500 MHz)

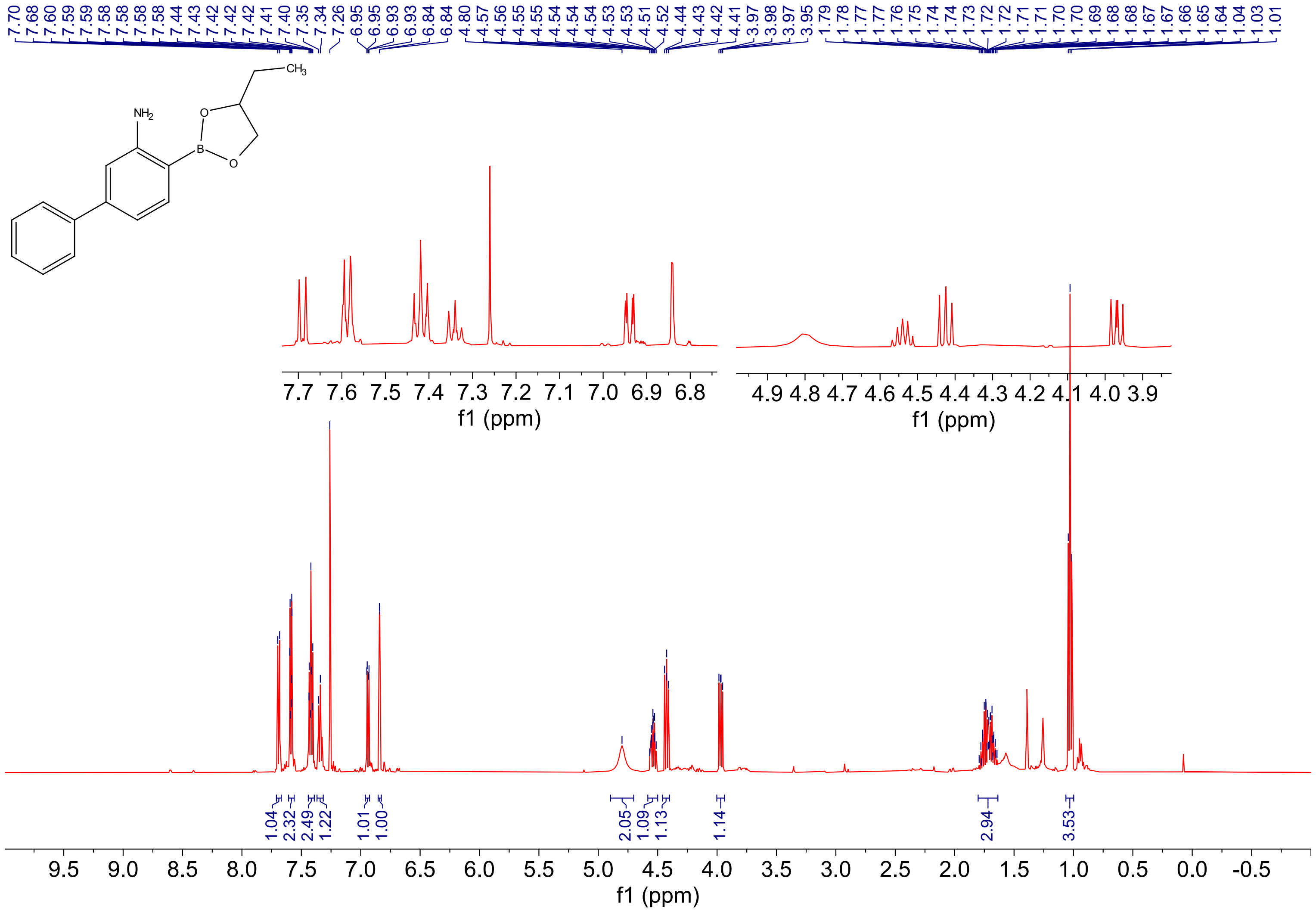

$^{13}C\{^1H\}$ NMR of *ortho* Bbg-borylated 3-aminobiphenyl (12) ($CDCl_3$, 160 MHz)

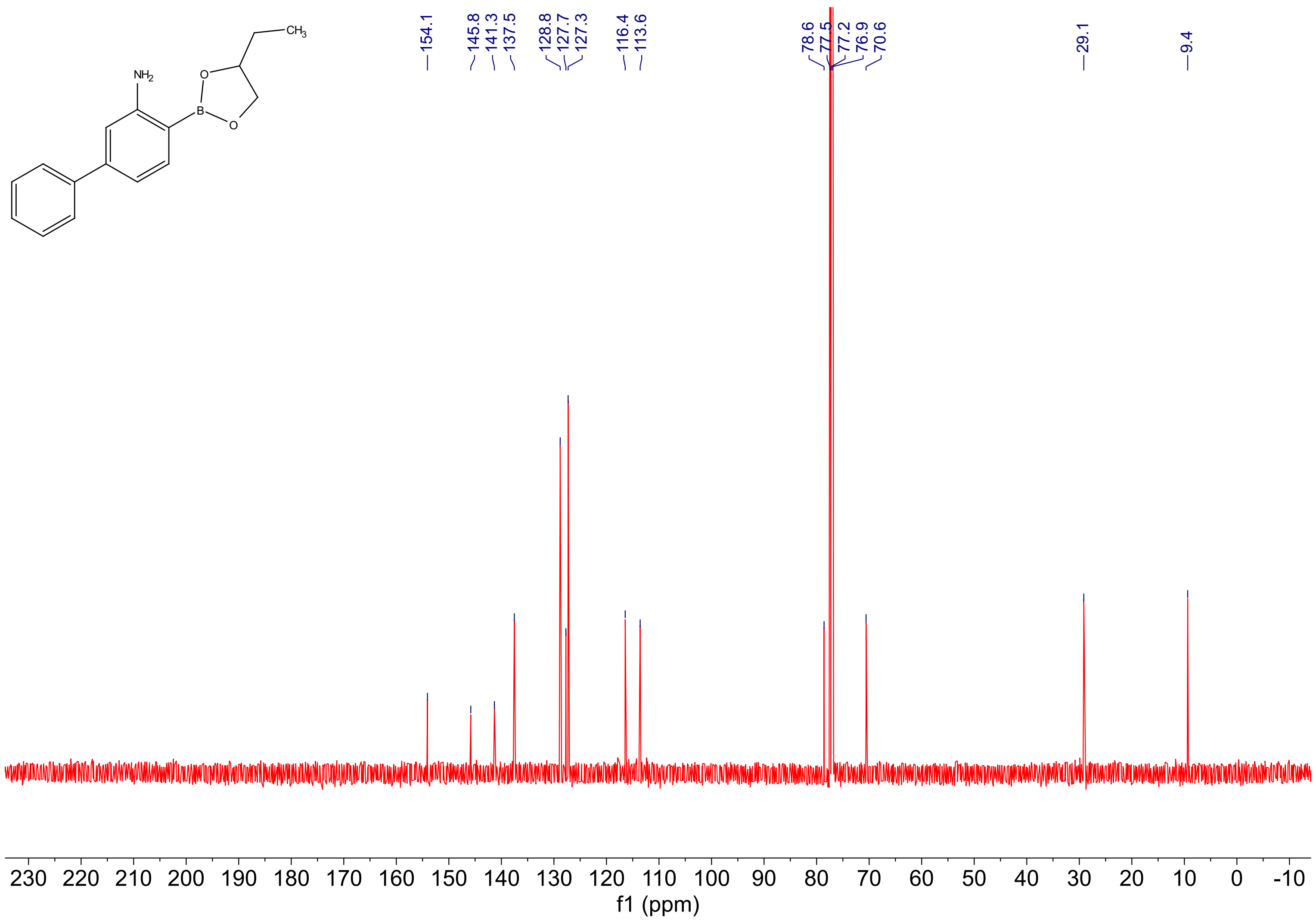

^{11}B NMR of *ortho* Bbg-borylated 3-aminobiphenyl (12) ($CDCl_3$, 126 MHz)

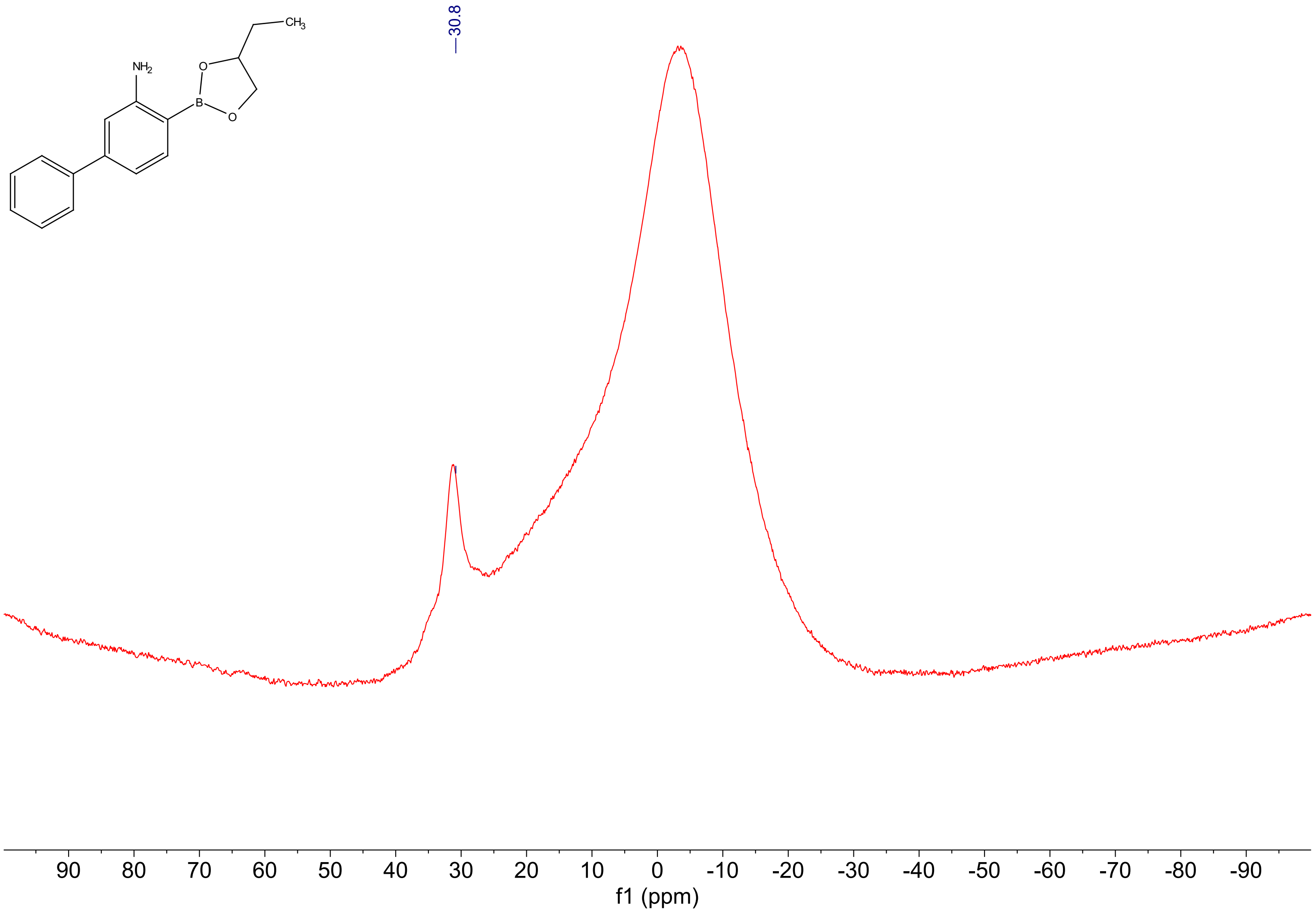

^{1}H NMR spectrum of the reaction mixture of CHB 2-methylaniline (13) ($CDCl_3$, 500 MHz)

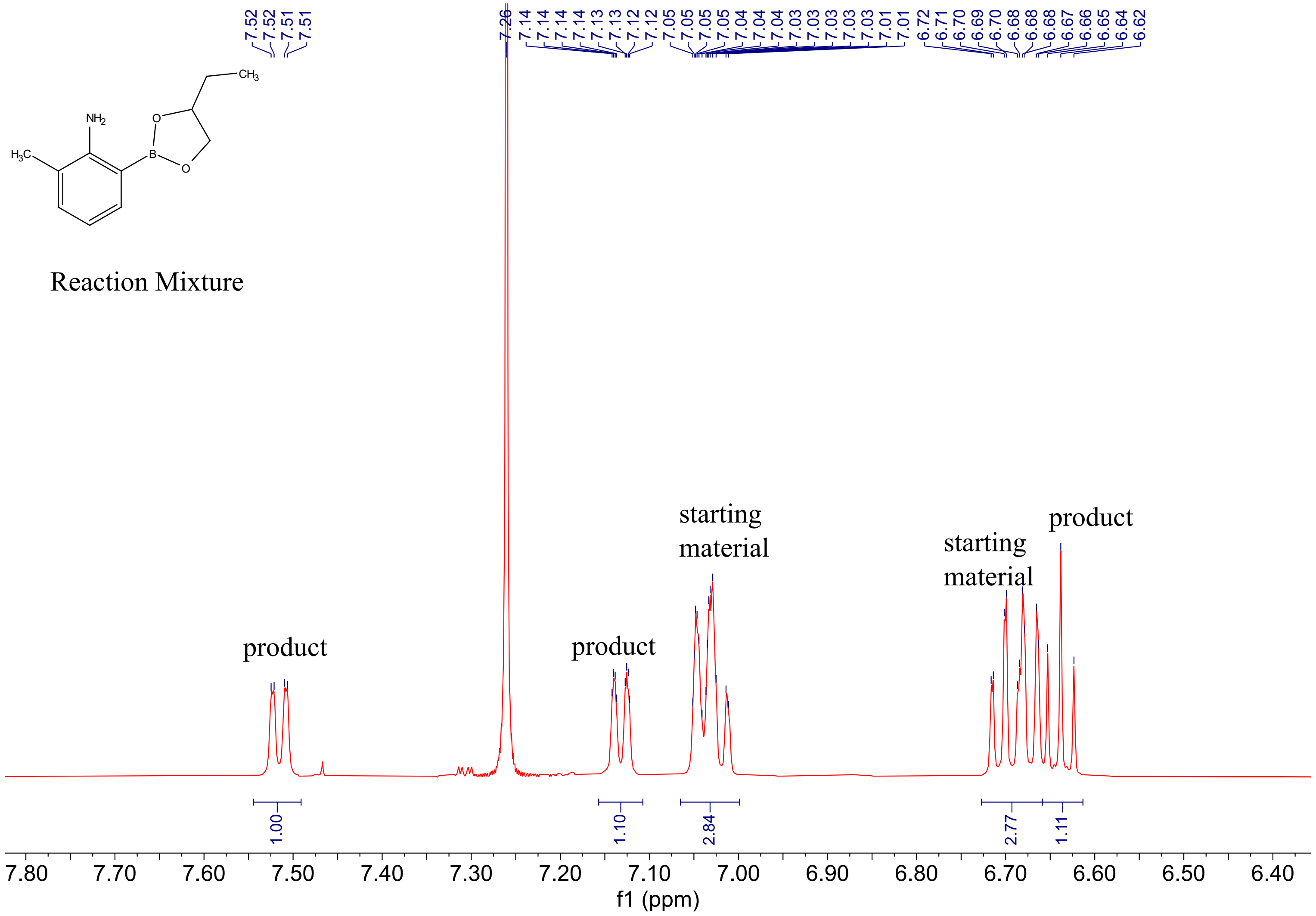

^{1}H NMR of *ortho* Bbg-borylated 2-methylaniline (13) ($CDCl_3$, 500 MHz)

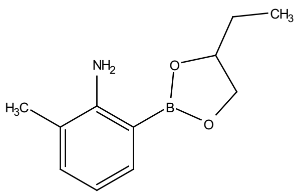

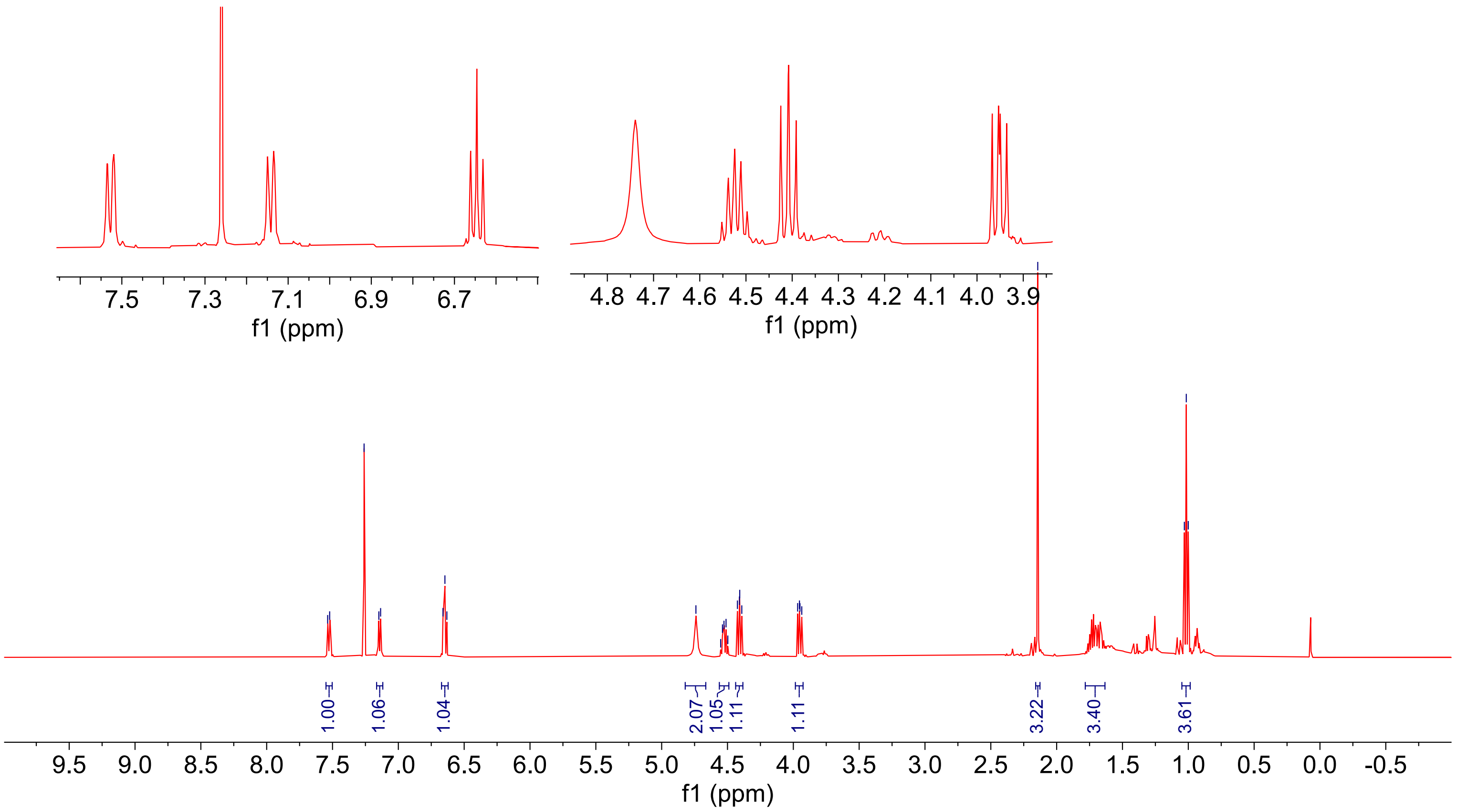

$^{13}C\{^1H\}$ NMR of *ortho* Bbg-borylated 2-methylaniline (13) ($CDCl_3$, 160 MHz)

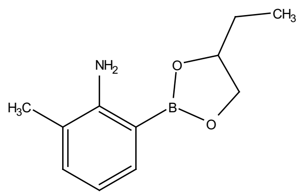

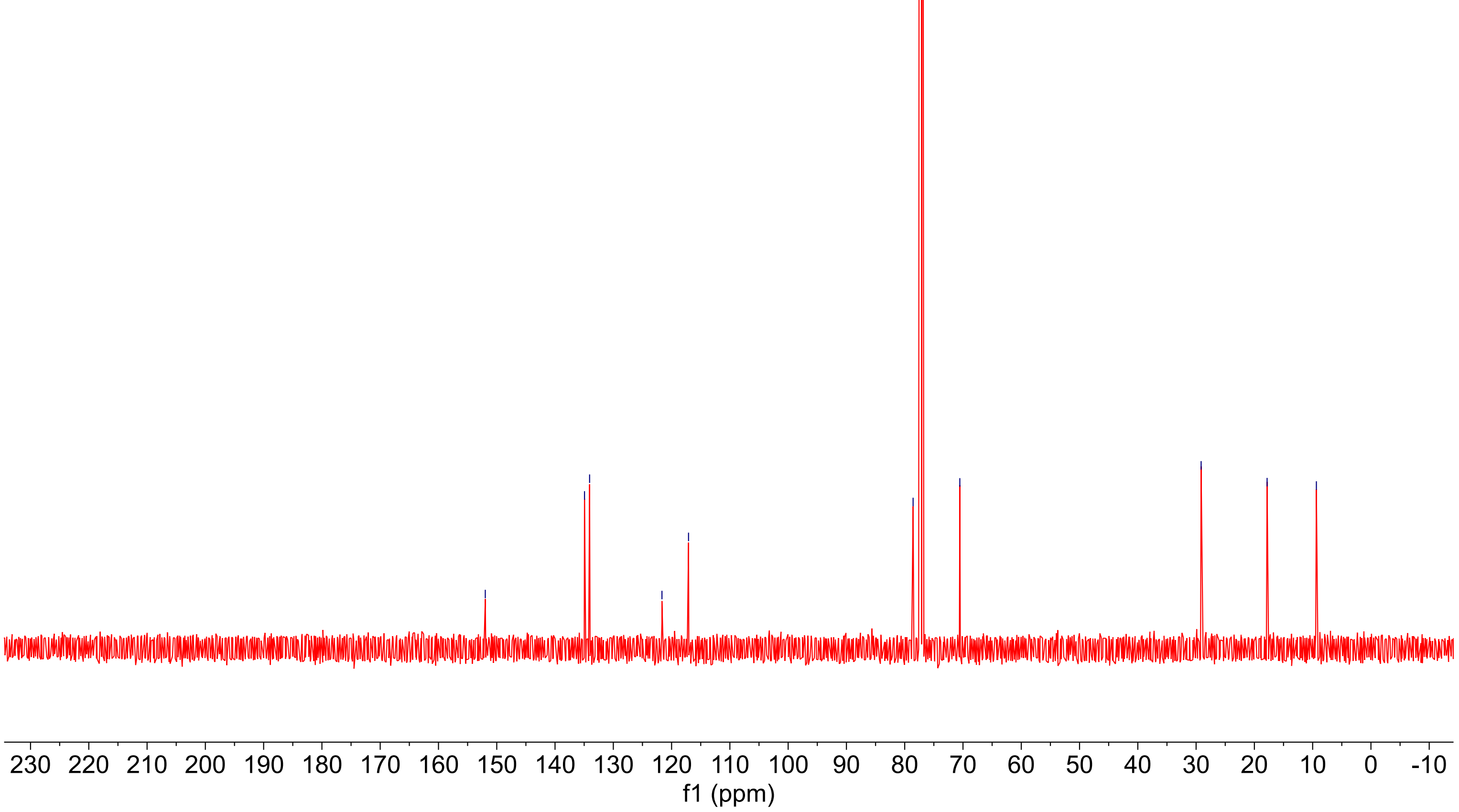

^{11}B NMR of *ortho* Bbg-borylated 2-methylaniline (13) ($CDCl_3$, 126 MHz)

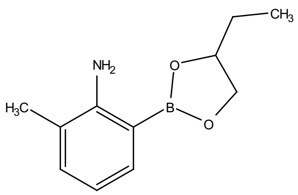

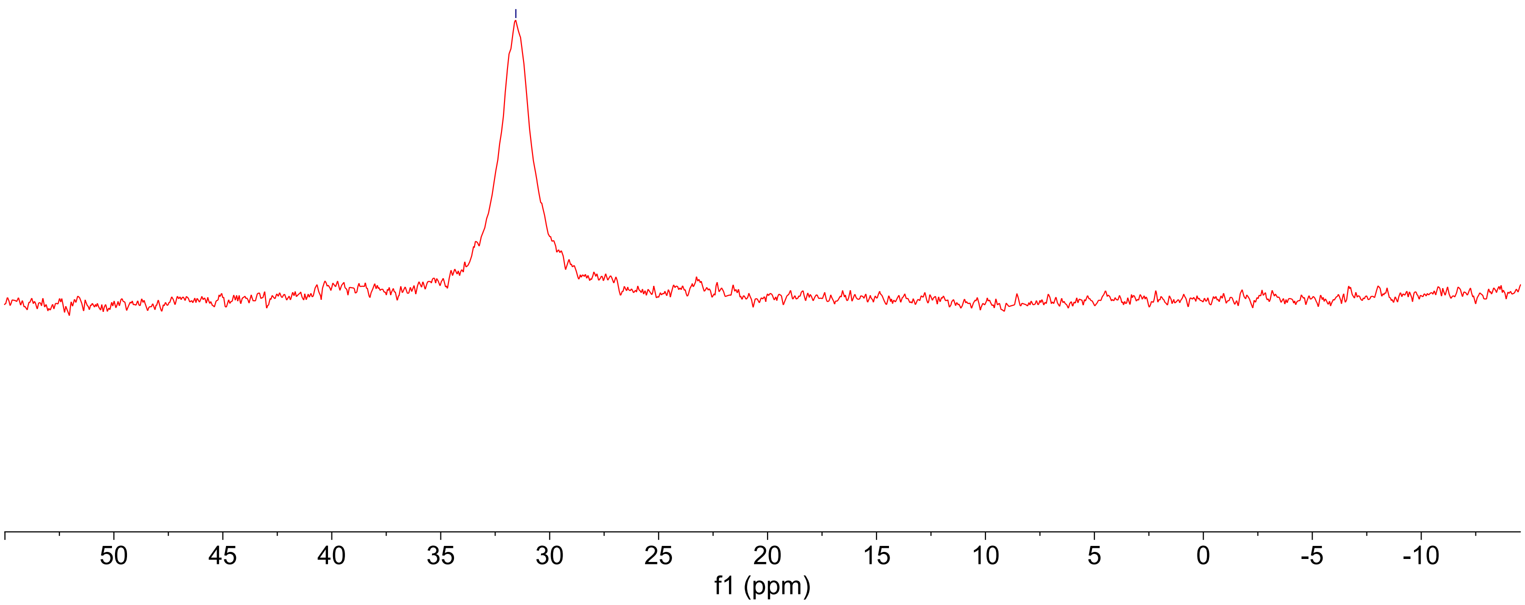

Supplement: Supplementary file 1 — ol4c01495_si_001.pdf [file ol4c01495_si_001.pdf]
